# Measuring the prevalence of 60 health conditions in older Australians in residential aged care with electronic health records: a retrospective dynamic cohort study

**DOI:** 10.1186/s12963-020-00234-z

**Published:** 2020-10-08

**Authors:** Kimberly E. Lind, Magdalena Z. Raban, Lindsey Brett, Mikaela L. Jorgensen, Andrew Georgiou, Johanna I. Westbrook

**Affiliations:** 1 Department of Health Promotion Sciences, Mel & Enid Zuckerman College of Public Health, University of Arizona, 3950 S. Country Club Rd., Suite 330, Tucson, AZ 85714 USA; 2 Centre for Health Systems and Safety Research, Australian Institute of Health Innovation, Macquarie University, Level 6, 75 Talavera Road, Sydney, NSW 2109 Australia; 3 Department of Health Professions, Faculty of Medicine and Health Sciences, Macquarie University, Ground Floor, 75 Talavera Road, Sydney, NSW 2109 Australia

**Keywords:** Health status, Multimorbidity, Multiple chronic conditions, Aged care, Long-term care, Nursing homes, Electronic health record

## Abstract

**Background:**

The number of older Australians using aged care services is increasing, yet there is an absence of reliable data on their health. Multimorbidity in this population has not been well described. A clear picture of the health status of people using aged care is essential for informing health practice and policy to support evidence-based, equitable, high-quality care. Our objective was to describe the health status of older Australians living in residential aged care facilities (RACFs) and develop a model for monitoring health conditions using data from electronic health record systems.

**Methods:**

Using a dynamic retrospective cohort of 9436 RACF residents living in 68 RACFs in New South Wales and the Australian Capital Territory from 2014 to 2017, we developed an algorithm to identify residents’ conditions using aged care funding assessments, medications administered, and clinical notes from their facility electronic health record (EHR). We generated age- and sex-specific prevalence estimates for 60 health conditions. Agreement between conditions recorded in aged care funding assessments and those documented in residents’ EHRs was evaluated using Cohen’s kappa. Cluster analysis was used to describe combinations of health conditions (multimorbidity) occurring among residents.

**Results:**

Using all data sources, 93% of residents had some form of circulatory disease, with hypertension the most common (62%). Most residents (93%) had a mental or behavioural disorder, including dementia (58%) or depression (54%). For most conditions, EHR data identified approximately twice the number of people with the condition compared to aged care funding assessments. Agreement between data sources was highest for multiple sclerosis, Huntington’s disease, and dementia. The cluster analysis identified seven groups with distinct combinations of health conditions and demographic characteristics and found that the most complex cluster represented a group of residents that had on average the longest lengths of stay in residential care.

**Conclusions:**

The prevalence of many health conditions among RACF residents in Australia is underestimated in previous reports. Aged care EHR data have the potential to be used to better understand the complex health needs of this vulnerable population and can help fill the information gaps needed for population health surveillance and quality monitoring.

## Introduction

An estimated 34 to 53% of Australians will enter a residential aged care facility (RACF; called nursing homes or long-term care facilities in other countries) during their lifetime [1]. In 2017, over 184,000 older Australians used residential aged care [2]. These numbers will continue to grow as our population ages, and the complexity and needs of these residents will increase as people stay at home longer and enter RACFs later in life [3, 4]. Older people in RACFs often have multiple chronic conditions, yet accurate information on the prevalence of conditions among this group is lacking. Also, in Australia as in other countries, complexity of older adults has not been adequately examined despite the clear need for research, services, and policy to address the complexity of older adults [5]. The Australian Institute for Health and Welfare (AIHW) has noted that underrepresentation of very old Australians and exclusion of people in permanent residential aged care are limitations of national health surveys [6], and national data on the number of Australians affected by common geriatric diseases—for example, dementia, which is the second leading cause of death in Australia [7, 8]—is limited. These data are critical for monitoring, planning, and improving service and care for older Australians.

One information source on the health status of older Australians living in RACFs is the Aged Care Funding Instrument (ACFI), which is used to determine the funding eligibility for RACF residents based on the assessment of core needs [9]. The ACFI has substantial limitations in terms of its actual purpose (i.e. to determine aged care funding levels) [10] and notable limitations for use in research; chronic conditions are likely underidentified since providers are only required to report the three most impactful conditions. Another source of data used to describe the health status data of older people is hospitalisation records, but these data only capture people who require hospitalisation which tend to be the most severe cases of a given condition.

Many RACFs are now using electronic health records (EHRs) to document residents’ health status, needs, and care administered (including medications). These EHRs are a rich source of clinical and healthcare use data about RACF residents. However, many EHR systems are not yet set up to easily report information such as the prevalence of specific conditions within a facility; thus, RACF EHR data remain underutilised. Leveraging existing RACF EHR data is a novel approach to filling the knowledge gap in the health status of Australia’s RACF population. Furthermore, understanding the burden of disease is vital for allocating funding for healthcare and research, prioritising research areas, and driving improvements in care.

The aims of this study were as follows: first, to provide a current epidemiological snapshot of the health status of a large sample of RACF residents in Australia in terms of condition rates and complexity; second, to develop a new model for using RACF EHR data to identify health conditions; and third, to evaluate agreement between ACFI data and EHR data to understand how maximising the use of EHR data will impact estimated disease rates.

## Methods

In 2017, there were a total 184,074 people living in RACFs Australia-wide and 65,248 in the states of New South Wales (NSW) and the Australian Capital Territory (ACT; Supplemental Table 2). The majority of RACFs in Australia are run by not-for-profit providers (58%), followed by private for-profit providers (33%), and government (9%); 70% of residents are located in major cities [11, 12].

We conducted a retrospective dynamic cohort study using routinely collected EHR data and ACFI assessments from a large not-for-profit aged care provider with 68 facilities across NSW and the ACT. This was a retrospective dynamic cohort as the data were collected before this study was initiated, originally for clinical purposes, and residents entered and left the facilities throughout the study period depending on when they entered or left RAC [13]. We included all residents who had an ACFI assessment and stayed at least 2 weeks in a facility during 1 January 2014 through 28 September 2017. ACFI assessments are conducted for all people entering permanent residential aged cared within 2 months of their admission, and typically before admission. Residents without an ACFI assessment are likely those that are entering facilities temporarily (e.g. for respite care). We excluded residents with stays shorter than 2 weeks because they were often missing data from two of the three main data sources we used. From the EHR, we extracted demographic data including age and sex. Length of stay was calculated by subtracting the residents’ entry date to the facility from the departure date or date at last observation. Health conditions were identified using three data sources: ACFI assessments, “special needs” text field of the residents’ EHR where conditions are recorded (by general practitioners (GPs) or registered nurses), and medications administered. Medications that are not typically used off-label with disease-specific indications and/or disease-dependent subsidisation by the Australian Pharmaceutical Benefits Scheme (e.g. denosumab use as an indication of osteoporosis) were identified by a research pharmacist.

We identified the date that a given condition was first reported within any data source. For chronic conditions (those that are typically long-lasting and take years to develop), we assumed that once a resident had the condition reported in any data source, the condition was present and permanent. For acute conditions, cases can be interpreted as those with a *history* of the acute condition (e.g. history of urinary tract infection). Supplemental Table 1 presents the specific conditions contained within each condition category and the criteria used to identify each condition; a corresponding SAS macro is freely available from our team website [14].

We calculated descriptive statistics for all variables of interest. Prevalence and 95% confidence intervals (CIs) for each condition by age and sex were estimated from a person-level dataset using mixed effects probit regression, with a random intercept for facility to account for clustering. We then applied these age- and sex-specific prevalence estimates to the 2016–2017 Australian RACF population figures from the AIHW [2] (Supplemental Table 2) to estimate the number of cases in Australia for each condition in 2016–2017.

ACFI data are the cleanest (i.e. conditions are standardised), most readily available data on health status for this population, and are used to report health conditions in previous studies. Thus, we compared conditions recorded in the ACFI to those recorded in the EHR data (medication administrations and free text notes). To assess agreement between conditions identified in ACFI compared to conditions identified in the EHR, we calculated Cohen’s kappa for each condition. Following convention, a kappa value of 0.01–0.20 indicated none to slight agreement, 0.21–0.40 fair agreement, 0.41–0.60 moderate agreement, 0.61–0.80 substantial, and 0.81–1.00 almost perfect agreement [15].

Multiple chronic conditions were analysed using latent class analysis (LCA) to identify comorbidity clusters (i.e. groups of residents with similar conditions) using the twenty most common conditions, age at admission, and sex. We used this approach rather than creating counts of the number of conditions for each resident, as the number of conditions does not always reflect complexity and is dependent on the precision of condition categories, which hinders comparisons across studies. Following the approach recommended by Dean and Raftery [16], we ran the analysis with two to ten clusters and then selected the number of clusters based on the Bayesian information criterion (smaller BIC values are considered better and changes in BIC of ≥ 10 are considered meaningful [17]). After identifying the optimal number of clusters, we calculated descriptive statistics for each cluster on demographic characteristics, length of stay, prevalence of specific conditions, and ACFI domain scores (measures of functional and cognitive status) within each cluster.

Analyses used a type I error rate of 0.05 and were conducted using SAS 9.4 (SAS Institute, Cary, NC, USA) and Stata 15 (Stata Corp, College Station, TX, USA).

## Results

### Sample characteristics

A total of 9436 residents were included in the analyses, accounting for 14.5% and 5.1% of the total NSW/ACT and Australian residential aged care populations, respectively. Sample size and exclusions are presented in Supplemental Figure 1. The majority of the residents were female (67.2%), aged 85–94 (50.3%), and were discharged or died during the study period (54.5%). A total of 263 centenarians were included, and the age and sex distribution of our sample was similar to the overall RACF population in Australia (Supplemental Table 3).

### Condition prevalence

Age- and sex-specific prevalence estimates for the top ten conditions ranked by estimated total number of cases in Australia in 2017 are presented in Table 1. Constipation had the greatest number of estimated national cases and highest overall prevalence in our sample (133,302; 76%), followed by hypertension (110,508; 62%), arthritis (106,116; 61%), dementia (104,698; 58%), peptic ulcer disease/gastro-oesophageal reflux disease (PUD/GORD) (104,279; 58%), depression (100,791; 54%), urinary incontinence (89,725; 51%), dyslipidaemia (85,320; 44%), pain (69,687; 39%), and chronic lower respiratory disease (64,113, 35%). Supplemental Table 4 presents age- and sex-specific prevalence estimates for all conditions; estimates are plotted in Supplemental Figure 2. Supplemental Table 5 presents case estimates for all conditions.

**Table 1:** Estimated prevalence and 95% confidence intervals for the ten most common conditions in RACF residents in Australia

Prevalence % (95% CI)		Age								Estimated total cases in Australian RACF residents
		65	70	75	80	85	90	95	100+	
**Constipation**	**F**	0.74 (0.69, 0.79)	0.73 (0.69, 0.76)	0.72 (0.69, 0.75)	0.72 (0.70, 0.75)	0.74 (0.71, 0.76)	0.76 (0.74, 0.78)	0.79 (0.77, 0.81)	0.83 (0.80, 0.85)	133,302
	**M**	0.68 (0.62, 0.73)	0.68 (0.65, 0.72)	0.70 (0.66, 0.73)	0.71 (0.68, 0.74)	0.73 (0.70, 0.75)	0.74 (0.71, 0.77)	0.76 (0.73, 0.80)	0.79 (0.73, 0.84)	
**Hypertension**	**F**	0.40 (0.34, 0.45)	0.50 (0.46, 0.53)	0.57 (0.55, 0.60)	0.63 (0.60, 0.65)	0.66 (0.64, 0.68)	0.67 (0.65, 0.69)	0.66 (0.64, 0.68)	0.63 (0.59, 0.66)	110,508
	**M**	0.43 (0.37, 0.48)	0.50 (0.47, 0.54)	0.56 (0.53, 0.59)	0.60 (0.57, 0.63)	0.62 (0.59, 0.64)	0.62 (0.59, 0.64)	0.59 (0.56, 0.63)	0.55 (0.49, 0.61)	
**Arthritis**	**F**	0.36 (0.31, 0.42)	0.45 (0.41, 0.49)	0.53 (0.50, 0.56)	0.59 (0.56, 0.62)	0.64 (0.61, 0.66)	0.67 (0.65, 0.69)	0.69 (0.67, 0.72)	0.70 (0.66, 0.73)	106,116
	**M**	0.32 (0.27, 0.37)	0.40 (0.36, 0.44)	0.47 (0.43, 0.50)	0.53 (0.50, 0.56)	0.59 (0.56, 0.61)	0.63 (0.60, 0.66)	0.67 (0.63, 0.71)	0.70 (0.64, 0.75)	
**Dementia**	**F**	0.44 (0.37, 0.51)	0.51 (0.44, 0.57)	0.55 (0.50, 0.61)	0.59 (0.53, 0.64)	0.61 (0.56, 0.66)	0.62 (0.57, 0.67)	0.61 (0.56, 0.67)	0.60 (0.54, 0.65)	104,698
	**M**	0.37 (0.30, 0.44)	0.47 (0.41, 0.54)	0.55 (0.49, 0.61)	0.59 (0.54, 0.65)	0.61 (0.55, 0.66)	0.59 (0.54, 0.65)	0.55 (0.49, 0.61)	0.47 (0.40, 0.55)	
**PUD, GORD, and other acid-related conditions**	**F**	0.52 (0.47, 0.57)	0.55 (0.51, 0.59)	0.57 (0.55, 0.60)	0.59 (0.57, 0.61)	0.60 (0.57, 0.62)	0.60 (0.58, 0.62)	0.59 (0.57, 0.61)	0.57 (0.54, 0.61)	104,279
	**M**	0.48 (0.42, 0.53)	0.51 (0.47, 0.55)	0.54 (0.51, 0.57)	0.56 (0.53, 0.59)	0.58 (0.55, 0.60)	0.59 (0.57, 0.62)	0.60 (0.56, 0.64)	0.61 (0.55, 0.67)	
**Depression**	**F**	0.65 (0.59, 0.70)	0.64 (0.59, 0.68)	0.62 (0.59, 0.66)	0.60 (0.57, 0.64)	0.58 (0.54, 0.61)	0.54 (0.50, 0.58)	0.50 (0.46, 0.54)	0.45 (0.41, 0.50)	100,791
	**M**	0.58 (0.52, 0.64)	0.57 (0.52, 0.62)	0.56 (0.52, 0.60)	0.54 (0.50, 0.58)	0.51 (0.47, 0.55)	0.48 (0.44, 0.52)	0.44 (0.39, 0.48)	0.39 (0.33, 0.45)	
**Urinary incontinence**	**F**	0.51 (0.45, 0.58)	0.52 (0.46, 0.57)	0.52 (0.47, 0.57)	0.52 (0.47, 0.57)	0.52 (0.48, 0.57)	0.53 (0.48, 0.57)	0.53 (0.49, 0.58)	0.54 (0.48, 0.59)	89,725
	**M**	0.41 (0.35, 0.47)	0.43 (0.37, 0.48)	0.44 (0.39, 0.48)	0.44 (0.39, 0.49)	0.44 (0.40, 0.49)	0.44 (0.40, 0.49)	0.44 (0.38, 0.49)	0.43 (0.36, 0.50)	
**Dyslipidaemia**	**F**	0.43 (0.37, 0.48)	0.49 (0.45, 0.53)	0.52 (0.49, 0.55)	0.52 (0.50, 0.54)	0.49 (0.47, 0.52)	0.44 (0.42, 0.46)	0.36 (0.34, 0.38)	0.26 (0.23, 0.29)	85,320
	**M**	0.39 (0.33, 0.44)	0.46 (0.42, 0.50)	0.51 (0.48, 0.54)	0.53 (0.50, 0.56)	0.52 (0.49, 0.55)	0.48 (0.45, 0.51)	0.41 (0.37, 0.45)	0.32 (0.26, 0.38)	
**Pain and pain syndromes**	**F**	0.34 (0.28, 0.39)	0.37 (0.33, 0.42)	0.40 (0.36, 0.43)	0.41 (0.38, 0.45)	0.42 (0.38, 0.45)	0.41 (0.38, 0.44)	0.39 (0.36, 0.43)	0.37 (0.33, 0.41)	69,687
	**M**	0.33 (0.27, 0.38)	0.34 (0.29, 0.38)	0.34 (0.31, 0.38)	0.35 (0.31, 0.39)	0.35 (0.32, 0.39)	0.36 (0.32, 0.39)	0.36 (0.31, 0.40)	0.35 (0.29, 0.42)	
**Chronic lower respiratory disease**	**F**	0.41 (0.35, 0.46)	0.39 (0.36, 0.43)	0.38 (0.35, 0.41)	0.37 (0.34, 0.39)	0.35 (0.33, 0.38)	0.34 (0.32, 0.36)	0.33 (0.31, 0.35)	0.32 (0.28, 0.35)	64,113
	**M**	0.34 (0.29, 0.39)	0.34 (0.30, 0.37)	0.34 (0.31, 0.37)	0.35 (0.32, 0.37)	0.36 (0.33, 0.38)	0.37 (0.34, 0.39)	0.38 (0.34, 0.42)	0.40 (0.34, 0.46)	

Model-based estimates of condition prevalence generated using mixed effects probit regression. Prevalence estimates are expressed as proportions. *N* = 9436 people in residential aged care during 2014–2017 from 68 facilities in NSW and ACT. Case estimates were generated by multiplying the age- and sex-specific prevalence estimates by the age and sex distribution of the Australian residential aged care population in 2017 based on GEN aged care data published by the Australian Institute of Health and Welfare. *F* estimates for females, *M* estimates for males, *PUD/GORD* peptic ulcer disease/gastro-oesophageal reflux disease. Detailed information on the specific conditions and criteria used in the condition categories presented in this table can be found in Supplemental Table 1

### Agreement between data sources

Agreement between ACFI and the EHR (resident condition notes and medications administered) varied by condition, but ranged from no agreement to fair agreement for a majority of the conditions. Table 2 presents agreement measures for the ten most common conditions (based on our estimates using all data sources) that appeared in the ACFI, and agreement measures for all conditions are presented in Supplemental Table 6 and Supplemental Figure 3. Two conditions had almost perfect agreement between EHR and ACFI: multiple sclerosis (kappa = 0.81) and Huntington’s disease (kappa = 0.86). Four conditions had substantial agreement between ACFI and EHR (kappa 0.61 to 0.80): diabetes, Parkinson’s disease, dementia, and schizophrenia, paranoid, or psychotic states. Notably, there was no agreement between ACFI and EHR data for having any circulatory disease (kappa = 0.12).

**Table 2:** Agreement between ACFI data and EHR data (*n* = 9436)

ACFI		EHR: resident notes and medications		Kappa (95% CI)	Overall prevalence %
		Without	With		
Hypertension	Without	3631	3185	0.31 (0.29, 0.32)	61.5
	With	315	2305		
Arthritis	Without	3711	1498	0.53 (0.52, 0.55)	60.7
	With	723	3504		
Dementia	Without	3980	249	0.77 (0.76, 0.78)	57.8
	With	840	4367		
Depression	Without	4352	598	0.60 (0.59, 0.62)	53.9
	With	1262	3224		
Urinary incontinence	Without	4602	1173	0.33 (0.31, 0.35)	51.2
	With	1760	1901		
Dyslipidaemia	Without	5271	3665	0.11 (0.10, 0.12)	44.1
	With	53	447		
Pain	Without	5793	1639	0.37 (0.35, 0.39)	38.6
	With	698	1306		
Chronic lower respiratory disease	Without	6145	2196	0.37 (0.35, 0.39)	34.9
	With	64	1031		
Heart disease	Without	6193	1770	0.36 (0.34, 0.38)	34.4
	With	423	1050		
Dry eyes and other eye issues	Without	6414	2771	0.03 (0.02, 0.05)	32.0
	With	122	129		

Overall (i.e. across the entire sample of residents) prevalence is calculated as the sum of those with a condition according to either or both data sources divided by the sample denominator of 9436. *ACFI* Aged Care Funding Instrument, *EHR* electronic health record

### Cluster analysis

**Fig. 1:**
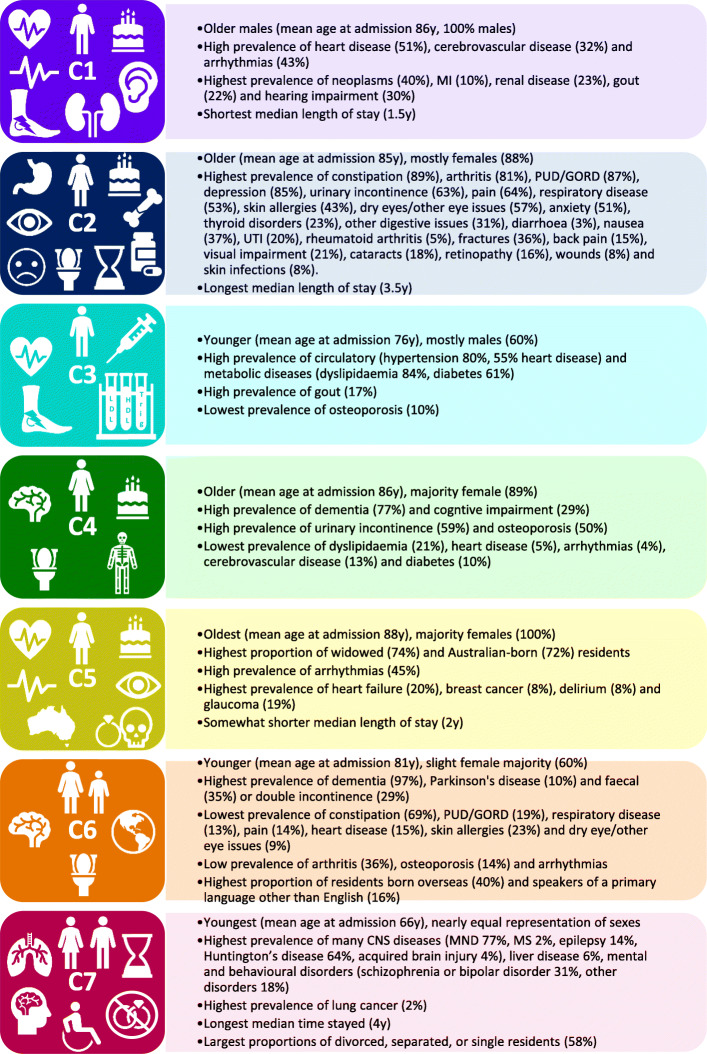
Comorbidity cluster summary. Comorbidity clusters were identified using latent class analysis. More detail on cluster characters is presented in Supplemental Tables 7–9 and Supplemental Figure 3. MND, motor neurone disease; MS, multiple sclerosis; Y, year; MI, myocardial infarction; UTI, urinary tract infection

Comorbidity cluster analysis results are presented in Fig. 1; Supplemental Tables 7, 8, and 9; and Supplemental Figure 4. We identified seven comorbidity clusters. Notably, cluster 2 (C2) was the most complex and had the longest length of stay, and C7 was the youngest cluster and had high rates of central nervous system diseases, liver disease, lung cancer, and mental and behavioural disorders. Dementia prevalence was notably varied across the clusters ranging from 36 to 96%, and there were two distinct dementia clusters C4 and C6, although C6 was younger than C4. There was variation in ACFI domain scores across the clusters (Supplemental Table 9); notably, C6 had high need for assistance with nutrition, C4 had the worst mobility and toileting, and both C6 and C4 needed the most assistance with personal hygiene and continence and had the worst ratings for cognition and wandering.

## Discussion

This study presents a new model for identifying health conditions among the RACF population using existing electronic health record data. We found that RACF residents had many comorbidities, and certain conditions were more likely to be underreported in ACFI data than others. Agreement between the ACFI and EHR data was none to fair for most conditions we examined. However, agreement between the ACFI and EHR was substantial for several conditions that typically require a high level of assistance due to physical disability and/or have complex medication management. Based on these results, we caution policymakers and researchers from drawing strong conclusions about condition prevalence based on ACFI data for most conditions with the exceptions of Huntington’s disease, multiple sclerosis, diabetes, Parkinson’s disease, dementia, and schizophrenia, paranoid, or psychotic states. We also identified seven distinct clusters of multiple chronic conditions and found that the sickest most complex cluster is the residents who have the longest stays (C2). We identified a young cluster (C7) of residents whose conditions are associated with high healthcare and social service needs likely extending prior to RACF admission based on their high prevalence of mental and behavioural conditions and conditions associated with substance use disorders (e.g. liver disease, lung cancer), and also their potential lack of social support (largest proportion of unmarried people). The clusters also varied in terms of functional and cognitive status, reflecting different care needs.

Circulatory diseases affect a large majority of residents—which was expected based on previous studies—however, unexpectedly constipation had the highest prevalence of all the conditions we analysed. Although constipation can be an acute condition, it can also occur chronically, and we found that it was indeed a recurring problem for residents. Many residents identified as having constipation were flagged due to laxative use. We examined laxative use trends in a post hoc analysis and found that among residents who used laxatives, the median number of laxative doses administered per resident during their stay was 684. RACF residents are at increased risk of constipation as a side effect of medications and other conditions, but constipation may be modifiable with ensuring optimal adequate hydration, physical activity, and fibre intake. Unfortunately, there has been a trend of increasing use of supplements paired with decreasing spending on fresh produce in this setting [18], which may be exacerbating the problem of chronic constipation. These data highlight the need for attention to this overlooked issue that can have a substantial impact on the quality of life of residents; the focus needs to shift from treatment of constipation to prevention. Many of the other conditions that were revealed as highly prevalent, such as depression, arthritis, dementia, and chronic lower respiratory disease, could be improved through a holistic approach to management that includes improvements in lifestyle behaviours, diet, physical activity, and social participation in meaningful activities [19] which are currently not funded in Australia.

Compared to previous studies that have used ACFI data alone, our study found generally higher rates of health conditions among aged care residents [20]. This was expected since the ACFI limits the number of conditions that can be reported. Furthermore, ACFI is not regularly updated; thus, new conditions starting after RACF admission are less likely to be recorded in ACFI compared to the EHR. These differences were more pronounced for some conditions. For example, we found higher prevalence rates based on residents’ EHR data for the three most common conditions compared to those identified with ACFI data alone: dementia (58% vs. 48%), depression (54% vs. 23%), and arthritis (61% vs. 14%) [21]. Our results suggest that ACFI data alone may generally underestimate condition prevalence, but the magnitude of underestimation varies by condition. Consistent with prior research, dementia can be reasonably well identified from ACFI data [22], but we suspect that dementia may still be underdiagnosed and underreported in this setting as previous research has shown [23]. This trend in underreporting of conditions is supported by condition prevalence reported in Aged Care Assessment Program (ACAP) data [20] (an assessment conducted before RACF admission—i.e. before the ACFI). ACAP allows up to ten conditions to be reported, and studies that used ACAP [20] data have found generally higher prevalence rates for conditions than those based on ACFI data, but both sources report generally lower rates than our estimates from EHR data [21]. As for survey data, our prevalence estimates for most conditions also tended to be higher; compared to estimates from the Australian Bureau of Statistics Survey of Disability, Ageing and Carers in 2015 for respondents in residential aged care, our prevalence estimates are similar for arthritis, but higher for osteoporosis, anxiety, depression, hypertension, and diabetes [24].

Our study has several strengths. The use of EHR data with notes on conditions, medication administration data, and ACFI assessments exploited all sources of existing relevant data in the aged care setting to provide the most comprehensive identification of conditions possible without primary data collection or time intensive chart reviews. This approach to identifying conditions is practical since it requires no new data collection in the facility. Another strength is that our large sample is demographically similar to the Australian aged care population, and we expect that our estimates are generalisable nationally. Also, our regression approach corrected for potential correlation in facility-level degree of reporting. Finally, our comorbidity cluster analysis provided a much more detailed illustration of co-occurring conditions beyond what simple counts of conditions can provide, and it highlighted the complexity and diversity of needs of the RACF population.

This study is limited by the use of data from a single RAC provider with facilities in NSW and ACT. However, we do not suspect the residents in our sample differ substantially from the average Australian RACF resident since our sample includes a notable proportion of the entire RACF population in the most populous Australian state (14.5% of NSW), and our sample is demographically similar to the Australian RACF population. Our findings have some limitations rooted in the fact that the ACFI and the EHR are designed respectively for funding purposes and for clinical care—not for epidemiological surveillance. There is likely some degree of underreporting of health conditions in the EHR, but the rate of underreporting and how it varies by condition is unknown. We suspect that some conditions may simply be more likely to be underdiagnosed and/or underreported, such as osteoporosis. Our estimates for osteoporosis prevalence were well below the 86% estimate in this setting in the USA [25], and osteoporosis was reported at lower rates than fractures for some clusters, although it is likely that most of these fractures are low-impact osteoporotic fractures. Lower prevalence of osteoporosis also occurred in clusters that were relatively more complex, which supports the hypothesis that more complex residents are less likely to have their osteoporosis documented and treated [26]. The gold standard for identifying conditions would entail examining each resident and reviewing historical external GP records (since the GP is likely to change once a person enters residential care) and hospital records; however, this was not practical or feasible for this large of a sample. We have taken the first steps to fully utilise data collected within aged care systems; linking hospital and historical GP data should be pursued in future studies.

## Conclusions

In summary, older Australians in RACFs are medically complex and prior reports have underestimated the prevalence of conditions in this population. This complexity makes the management of these patients challenging for the GPs and the RACF clinical staff who care for them, and it increases the risk of poor care management at transitions of care when an accurate clinical picture is needed urgently. We caution researchers and policymakers using aged care assessment data and self-reported condition data to be mindful of underreporting of conditions, and encourage others to use EHR data to the fullest extent possible. EHR data have yet to achieve their full potential as a tool for supporting the provision of evidence-based care and for monitoring conditions, outcomes, and quality indicators in the residential aged care setting.

## Supplementary information

**Additional file 1: Supplemental Table 1.** Criteria used to identify conditions in the ACFI assessments, EHR and medication administration records. **Supplemental Table 2.** RACF population in Australia 2016-17. **Supplemental Table 3.** Sample characteristics (*n*=9436). **Supplemental Table 4.** Age and sex-specific estimates of condition prevalence (proportions) with 95% confidence intervals. **Supplemental Table 5.** Estimated number of cases in Australian RACFs in 2016-17 by age and sex. **Supplemental Table 6.** Agreement between ACFI data and EHR data (*n*=9436). **Supplemental Table 7.** Cluster Analysis – Comorbidity Cluster Characteristics. **Supplemental Table 8.** Resident Characteristics by Comorbidity Cluster. **Supplemental Table 9.** ACFI domains: Functional and cognitive ratings by cluster.

**Additional file 2: Supplemental Figure 1.** Sample size and exclusions. **Supplemental Figure 2.** Age and sex specific prevalence estimates, **Supplemental Figure 3.** Kappa estimates for agreement between conditions recorded in the aged care funding instrument (ACFI) and the electronic health record (EHR). **Supplemental Figure 4.** Condition Prevalence by Comorbidity Cluster

## Data Availability

The datasets generated and analysed during the current study are not publicly available due to our memorandum of understanding with the participating aged care provider which explicitly limits disclosure of data. Coding algorithms to identify conditions are freely available for download from our team website at: https://bit.ly/3liyNXp.

## Abbreviations

ACFI: Aged Care Funding Instrument
AIHW: Australian Institute for Health and Welfare
EHR: Electronic health record
GP: General practitioner
LCA: Latent class analysis
RACF: Residential aged care facility
