# Supplementary material for: Measuring the prevalence of 60 health conditions in older Australians in residential aged care with electronic health records: a retrospective dynamic cohort study
Source: Popul Health Metr. 2020 Oct 8;18:25. doi: 10.1186/s12963-020-00234-z (PMC7545887; doi:10.1186/s12963-020-00234-z)
Supplement: Supplementary file 1 — Additional file 1: Supplemental Table 1. Criteria used to identify conditions in the ACFI assessments, EHR and medication administration records. Supplemental Table 2. RACF population in Australia 2016-17. Supplemental Table 3. Sample characteristics (n=9436). Supplemental Table 4. Age and sex-specific estimates of condition prevalence (proportions) with 95% confidence intervals. Supplemental Table 5. Estimated number of cases in Australian RACFs in 2016-17 by age and sex. Supplemental Table 6. Agreement between ACFI data and EHR data (n=9436). Supplemental Table 7. Cluster Analysis – Comorbidity Cluster Characteristics. Supplemental Table 8. Resident Characteristics by Comorbidity Cluster. Supplemental Table 9. ACFI domains: Functional and cognitive ratings by cluster. [file 12963_2020_234_MOESM1_ESM.docx]

Supplemental Table 1. Criteria used to identify conditions in the ACFI assessments, EHR and medication administration records

| **Category** | **Condition** | **ACFI conditions included** | **EHR free text included** | **Medications administered (ATC codes)** |
| --- | --- | --- | --- | --- |
| Circulatory | Any circulatory condition (includes these conditions plus all specific conditions below) | Other heart diseases (pulmonary embolism, acute pericarditis, acute and subacute endocarditis, cardiomyopathy, cardiac arrest, heart failure-unspecified), Other heart diseases e.g. pulmonary embolism, acute pericarditis, acute and subacute endocarditis, cardiomyopathy, cardiac arrest, heart failure, Other diseases of the circulatory system n.o.s. or n.e.c. (includes other peripheral vascular disease, arterial embolism and thrombosis, other disorders of arteries and arterioles, diseases of capillaries, varicose veins, haemorrhoids), Other diseases of the circulatory system, Hypotension (low blood pressure), Other arterial or aortic aneurysms (includes thoracic, unspecified, aneurysm of carotid artery, renal artery, unspecified); Abdominal aortic aneurysm | "CORONARY", "CARDI", "STENTS", "CABG", " CAD", "MVR", "TRICUSPID REGUR", "AORTIC", "CARDIOMYOPATHY", "VENTRICULAR", "PERICARD", "MURMUR", "PULMONARY EMB", "PERIPHERAL VASC", "PVD", "VENOUS THROMB", "VEIN THROMB", "RAYNAUD", "ARTERITIS", "VASCULITIS" | C; except C05, B01AA03, B01AF, B01AC |
|  | Heart disease | "Atherosclerosis", "Acute and chronic ischaemic heart disease", "Heart disease", "Rheumatic heart disease", "Angina" | "HEART DISEASE", "ATHERO", "ANGINA", "RHEMAUTIC HEART", "ISCHAEMIC HEART", "CVD", "CARDIAC DISEASE", "HD" | C01DA, C01DX16 |
|  | Hypertension | Hypertension (high blood pressure) | "HYPERTEN", " HT", "HT", "HIGH B", "HBP", "HYPTENSION" |  |
|  | Heart failure | Congestive heart failure (congestive heart disease) | "CONGESTIVE HEART", "HEART FAILURE", "CARDIAC FAILURE", "CHF", "CCF", "DIASTOLIC DYSFUNCTION", "VENTRICULAR DYSFUNCTION", "CHD" |  |
|  | Abnormal heart rhythms (arrhythmias) |  | "ARRHYTHMIA", "ARRYTHMIA", "TACHYCARDIA", "BRADYCARDIA", "FLUTTER", "FIBRILLATION", "FIBRILATION", " AF", "SVT", "PACEMAKER", "RAPID AF", "PAROXYSMAL AF", "PAF", " AF,", ",AF,", "HEART BLOCK", "BUNDLE BRANCH BLOCK", "LBBB", "AV BLOCK", "SICK SINUS SYNDROME". Note: "NOT FOR PACEMAKER" is removed from the flagged text strings. | C01EB10, C01BD01, C01BA03, C07AB09, C01BC04, C07AA07, C01AA05 |
|  | Myocardial Infarction (MI) | Myocardial infarction (heart attack) | "MYOCARDIAL", " MI ", "MI ", " MI,", ",MI,", " AMI", ",AMI", " MI;", ";MI;", ";AMI", "HEART ATTACK" |  |
|  | Dyslipidaemia | High cholesterol | "CHOLEST", "DYSLIPID", "HYPERCHOL" | C10 |
|  | Stroke & Cerebrovascular diseases (includes TIA) | Stroke (CVA) – cerebrovascular accident unspecified', 'Stroke (CVA)-cerebrovascular accident unspecified', 'Subarachnoid haemorrhage', 'Cerebral infarction', 'Cerebrovascular disease', 'Intracerebral haemorrhage', 'Transient cerebral ischaemic attacks (T.I.A.s)', 'Other intracranial haemorrhage' | "STROKE", "CVA", "INTRACRANIAL", "SUBARACHNOID", "CEREB", "FRONTAL LOBE INFARCT", "CEREBELLAR INFARCT", "CEREBRAL INFARCT", "TIA,", " TIA", "TIA;", ";TIA", ",TIA", ",TIA;" |  |
|  |  |  |  |  |
| Neoplasms |  | "Brain cancer", "Breast cancer", "Colorectal (bowel) cancer", "Head and neck cancer", "Lung cancer", "Prostate cancer", "Skin cancer", "Stomach cancer", "Leukaemia", "Non-Hodgkin’s lymphoma", "Other malignant tumours n.o.s. or n.e.c.", "Other neoplasms (includes benign tumours and tumours of uncertain or unknown behaviour)" | "CANCER", "LEUKAEMIA", "LYMPHOMA", "TUMOUR", "LEUKEMIA", "TUMOR", " CA ", "CA ", " CA,", "CA,", "CARCINOMA", "ADENOCARCINOMA", "METAST", "METS", "MYELOMA", "MENINGIOMA", "ADENOMA", "NEOPLAS", "CLL", "BCC", "NEUROMA". Note that "GROMMETS" must be dropped out after flagging "METS" | L01, L02AE, L02B, D06BB10 |
|  | Breast cancer | Breast cancer | "BREAST CA", "CA BREAST" | L02BA, L02BG, L02AE, L01XE07, L01XC13, |
|  | Prostate cancer | Prostate cancer | "PROSTATE CA", "PROSTATIC CA", "CA PROSTATE" | L02BB, L02AE, L02BX |
|  | Lung cancer | Lung cancer | "LUNG CA", "CA LUNG" | L01XE13, L01XE36, L01XE28, L01XE16, L01XE02, L01XE31, L01XE35, |
|  | Colorectal (bowel) cancer | Colorectal (bowel) cancer | "COLORECTAL CA", "BOWEL CA", "CA BOWEL", "COLON CA", "CA COLO", "RECTAL CA" |  |
|  | Skin cancer | Skin cancer | "SKIN CA", "CA SKIN", "BCC", "BASAL CELL", "SCC", "MELANOMA", "BOWENS DISEASE", "SQUAMOUS CELL" | D06BB10, L01BC02 (topical), L01XD03, L01XE38, L01XE23, L01XE25, L01XE15, L01XC11, L01XX48, L01XX43 |
|  | Blood & lymph cancers | Leukaemia; Non-Hodgkin’s lymphoma | "LEUKAEMIA", "LYMPHOMA", "LEUKEMIA" | L01XE06, L01XE27, L01XX47, L01XE01, L01XE08, L01XE24, L01XE18, L01XC19, L01XC15, L01XC10, L01XX27, L01XX14, |
|  | Metastatic cancer |  | METAST, "METS". Note that "GROMMETS" must be dropped out after flagging "METS" |  |
|  |  |  |  |  |
| Respiratory |  |  |  |  |
|  | Chronic respiratory disease | Chronic lower respiratory diseases (includes emphysema, chronic obstructive airways disease (COAD), asthma); Chronic lower respiratory diseases e.g. emphysema, chronic obstructive airways disease, asthma | "EMPHYSEMA", "CHRONIC OBSTRUCTIVE AIRWAY", "CHRONIC OBSTRUCTIVE PULM", "ASTHMA", "COAD", "COPD", "CHRONIC LOWER RESP", "CHRONIC AIRWAY LIMITATION" | R03A, R03B, R03DA04, R03DA05, R03DX |
|  |  |  |  |  |
| Endocrine | Any endocrine disorder (includes also thyroid and diabetes) | Other endocrine, nutritional and metabolic disorders n.o.s. or n.e.c. (includes hypoparathyroidism, Cushing’s syndrome) | "GLUCOSE", "SIADH", "ADDISIONS" | A10A, A10B, A10X, H03A, H03B. |
|  | Diabetes | Diabetes mellitus - other specified/ unspecified; Diabetes mellitus - other specified/unspecified/unable to be specified; Diabetes mellitus - type 1 (IDDM); Diabetes mellitus - type 2 (NIDDM); Diabetes mellitus type 1; Diabetes mellitus type 2 | "DIAB", "NIDD", "IDDM", "DM", "INSULIN" | A10A, A10B, A10X |
|  | Disorders of the thyroid gland | Disorders of the thyroid gland, Disorders of the thyroid gland (includes iodine-deficiency syndrome, hypothyroidism, hyperthyroidism, thyroiditis) | "THYRO", "GRAVES DIS", "GOIT" | H03A, H03B |
|  |  |  |  |  |
| Diseases of the nervous system | Any disease of the CNS (includes specific conditions below too) | Other diseases of the nervous system n.o.s. or n.e.c. (includes dystonia, migraines, headache syndromes, sleep disorders e.g. sleep apnoea and insomnia, Bell’s palsy, myopathies, peripheral neuropathy, dysautonomia); Other diseases nervous system; Blackouts, fainting, convulsions; Meningitis and encephalitis (excluding ‘viral’); Paralysis – non-traumatic e.g. hemiplegia, paraplegia, quadriplegia, tetraplegia and monoplegia; excludes spinal cord injury code 1699; Paralysis-non-traumatic (includes hemiplegia, paraplegia, quadriplegia, tetraplegia and other paralytic syndromes, e.g. diplegia and monoplegia; excludes spinal cord injury code 1699) | "DYSTONIA", "MIGRA", "PALSY", "L MYOPATH", "MYOPATHIES", "NEUROPATH", "NEPHROPATH", "CEPH" | N04BC07, N04BD02, N04BD01, N04BX02, N04BA, N04BB01, N07XX02, L04AC01, N07XX09, N07XX07, L04AA27, L03AX13, L03AB07, L03AB08, L03AB13, L04AA23, L04AA36, L04AA31. |
|  | Parkinson's disease | Parkinson’s disease; Parkinson’s disease (includes Parkinson’s disease, secondary Parkinsomism) | "PARKIN", " PD" | N04BC07, N04BD02, N04BD01, N04BX02, N04BA, N04BB01 |
|  | Motor neurone disease (including ALS) | Motor neurone disease | "MOTOR NEURO", "LOU GEHRIG", "ALS ", "AMYOTROPHIC LATERAL SCLEROSIS" | N07XX02 |
|  | MS | Multiple sclerosis | "MULTIPLE SCLEROSIS", " MS,", "MS," | L04AC01, N07XX09, N07XX07, L04AA27, L03AX13, L03AB07, L03AB08, L03AB13, L04AA23, L04AA36, L04AA31 |
|  | Epilepsy | Epilepsy | "EPILEP", "SEIZURE", "CONVLUSION" |  |
|  | Huntington’s disease | Huntington’s disease | "HUNTINGTON" |  |
|  | Brain injury (including TBI) |  | "BRAIN INJURY", "TBI" |  |
|  |  |  |  |  |
| Diseases of the digestive system |  |  |  |  |
|  | Other digestive condition (including inflammatory conditions) | Diseases of the intestine (includes stomach/ duodenal ulcer, abdominal hernia (except congenital), enteritis, colitis, vascular disorders of intestine, diverticulitis, irritable bowel syndrome, diarrhoea, constipation); Diseases of the intestine, ulcers, hernias (except congenital), enteritis, colitis, vascular disorders of intestine, diverticulitis, irritable bowel syndrome, diarrhoea, constipation; Other diseases of the digestive system e.g. disease of the oral cavity, salivary glands and jaws, oesophagitis, gastritis and duodenitis, cholecystitis, other diseases of the gallbladder, pancreatitis, coeliac disease; Other diseases of the digestive system n.o.s. or n.e.c. (includes diseases of oral cavity, salivary glands and jaws, oesophagitis, gastritis and duodenitis, cholecystitis, other diseases of gallbladder, pancreatitis, celiac disease) | "HERNIA", "PANCREATITIS", "CROHN", "DIVERTICUL", "COELIAC", "CELIAC", "COLITIS", "GASTRITIS", "GASTROPARESIS", |  |
|  | Disease of the liver | Chronic liver disease, Diseases of the liver e.g. alcoholic liver disease, toxic liver disease, fibrosis and cirrhosis of liver | "LIVER", "HEPATIC", "CIRRHOSIS" |  |
|  | Peptic ulcer disease, Gastro-oesophageal reflux disease, ulcers (gastric, oesophageal, duodenal), and other acid-related digestive conditions (PUD/GORD) |  | "GORD", "REFLUX", "OESOPHAG", "DYSPEPSIA", "ACHALASIA", "PEPTIC", "PUD", "GASTRIC ULCER", "STOMACH ULCER", "DUODENAL ULCER", "UPPER GI BLEED" | A02 |
|  | Diarrhoea | Diarrhea and gastroenteritis of presumed infectious origin, Diarrhoea and gastroenteritis of presumed infectious origin | "DIARR" | A07DA, A07C, |
|  | Constipation |  | "CONSTIP" | A06 |
|  | Nausea, vomiting | Nausea and vomiting | "NAUS", "VOMIT" | A04, A03FA01, N05AB04 |
|  | Faecal Incontinence | Bowel/ faecal incontinence; Incontinence – bowel/ faecal | "CAL INCONT", "BOWEL INCONT", "INCONTINENT F", "INCONTINENCE F", "URINE/FAECAL INCONT", "DUAL INCONT", "DOUBLE INCONT","INCONTINENT URINE/F", "INCONTINENT URINE & F", "DOUBLY INCONT", "URINARY FAECAL INCONT", "URINARY BOWEL INCONT", "INCONTINENT DUAL" |  |
|  | Double incontinence |  | URINE/FAECAL INCONT, "DUAL INCONT", "DOUBLE INCONT","INCONTINENT URINE/F", "INCONTINENT URINE & F", "DOUBLY INCONT", "URINARY FAECAL INCONT", "URINARY BOWEL INCONT", "INCONTINENT DUAL" (plus people who had urinary and faecal incontinence flagged) |  |
|  |  |  |  |  |
| Diseases of the urinary system |  |  |  |  |
|  | Renal system diseases | Kidney and urinary system- renal failure, cystitis | "KIDNEY", "RENAL", "DIALYSIS", "CRF", "CKD", "ARF" |  |
|  | Urinary incontinence | Stress/ urinary incontinence (includes stress, overflow, reflex and urge incontinence); Unspecified urinary incontinence; Incontinence – urinary (stress, overflow etc- do not include unspecified) | "URINE INCONT", "URINARY INCONT", "INCONTINENT URI", "INCONTINENCE URI", "URGE INCONT", "STRESS INCONT" | G04BD, H01BA02 |
|  | Urinary tract infection | Urinary tract infection | " UTI", "URINARY TRACT INFECTION", "CYSTITIS", "UTIRECURRENT", "UTI" |  |
|  |  |  |  |  |
| Mental, behavioural, psychiatric or neurodevelopmental disorders | Any mental/behavioural |  |  | N05A, N06A, N06D |
|  | Other mental/behavioural | Other mental and behavioural disorders e.g. due to alcohol or psychoactive substances (includes alcoholism, Korsakovs psychosis), adult personality and behavioural disorders; Brain disease/ disorders (includes senile degeneration of brain n.e.c., degeneration of nervous system due to alcohol, Schilder’s disease) | "PERSONALITY DISORDER", "KORSAKO", "ETOH", "ETHANOL", "ALCOHOL" |  |
|  | Dementia | Dementia, Alzheimers disease including early onset, late onset, atypical or mixed type or unspecified; Vascular dementia e.g. multi-infarct, subcortical, mixed; Dementia in other diseases, e.g. Picks Disease, Creutzfeldt-Jakob, Huntingtons, Parkinsons, HIV; Other dementias, e.g. Lewy Body, alcoholic dementia, unspecified | "DEMENTIA", "ALZH" |  |
|  | MCI/cog impairment/memory loss | Amnesia (memory disturbance, lack or loss) | "COGNITIVE IMPAIRMENT", "CONGNITIVE IMPAIRMENT", "COGNATIVE IMPAIRMENT", "COGNITIVE DECLINE", "COGNITIVE CHANGES", "COGNITIVE IMPAIREMENT", "COGNITIVE DEFICIT", "REDUCED COGNITION", "COGNITIVE LOSS", "POOR COGNITION", "IMPAIRED COGNITION", "AMNESIA", "MEMORY", "FORGETFUL", "STML", "STM LOSS", "LTML" |  |
|  | Schizophrenia, paranoid or psychotic states | Psychoses e.g. schizophrenia, paranoid states | "SCHIZO", "PARANOI", "PSYCHOS", "PSYCHOT", "ANTIPSYCHOTIC", "WITHOUT PSYCHOTIC" |  |
|  | Delirium | Delirium | "DELIRIUM", "DELERIUM", "DELIRUM" |  |
|  | Depression, mood and affective disorders | Depression, mood and affective disorders, bipolar disorder | "DEPRESS", "MOOD DISORDER", "AFFECTIVE", "EFFECTIVE DISORDER", "BIPOLAR", "BI-POLAR", "BI POLAR", |  |
|  | Anxiety and stress-related disorders | Neurotic, stress related, anxiety, somatoform disorders e.g. post traumatic stress disorder, phobic and anxiety disorders, nervous tension/stress, obsessive-compulsive disorder | "ANXI", "PANIC", "PTSD", "TRAUMATIC STRESS" |  |
|  |  |  |  |  |
| Musculoskeletal and soft tissue | Other musculoskeletal and soft tissue | Other soft tissue/ muscle disorders (includes rheumatism); Other disorders of the musculoskeletal system and connective tissue n.o.s. or n.e.c. (includes osteomyelitis) | "RHEUMATISM", "HIP DYS", "PAGET", "OSTEOM", "ROTATOR CUFF TEAR", "SUPRASPINATUS TEAR", "TENDON TEAR", "BURSITIS", "CYST", "TRIGGER FINGER", "DE QUERVAIN", "FROZEN SHOULDER", "CAPSULITIS", "IMPINGEMENT", "SPUR", "TENDONIT", "CONTRACTURE", "FASCITIS", "FACSCIITIS" |  |
|  | Osteoporosis | Osteoporosis | "OSTEOP", ",OP,", "OP ", ", OP,", "OP," | M05B, G03XC01, H05AA02 |
|  | Arthritis (all types) | Other arthritis and related disorders (e.g. gout, arthrosis, osteoarthritis), Other arthritis and related disorders (includes gout, arthrosis, osteoarthritis), Rheumatoid arthritis | "ARTHR"," OA", ",OA", " RA ", "RA,", ",RA,", " RA,", " RA;", "GOUT", "TOPH", "HYPERURIC" | M04 |
|  | Rheumatoid arthritis | Rheumatoid arthritis | "RHEUMATOID ARTHR", " RA ", "RA,", ",RA,", " RA,", " RA;" |  |
|  | Gout |  | "GOUT", "TOPH", "HYPERURIC" | M04 |
|  | Fracture | Fracture of femur (includes hip (neck of femur); Fracture of the femur (includes hip); Fracture at wrist and hand level; Fracture of lower leg and foot; Fracture of lumbar spine and pelvis (includes lumbar vertebra, sacrum, coccyx, sacrum); Fracture of neck (includes cervical spine and vertebra); Fracture of rib(s), sternum and thoracic spine (includes thoracic spine and vertebra); Fracture of rib(s), sternum and thoracic spine and vertebra; Fracture of shoulder, upper arm and forearm (includes clavicle, scapula, humerus, radius, ulna); | "FRACTUR", "#", "PUBIC RAMI", "FEMUR", "OLECRANON" |  |
|  | Amputation | Amputation of the finger/ thumb/ hand/ arm/ shoulder - traumatic; Amputation of toe/ ankle/ foot/ leg; Amputation of toe/ ankle/ foot/ leg - traumatic | "AMPUTAT", "BKA" |  |
|  | Back & spine conditions | Back problems - dorsopathies (includes scoliosis) | "BACK PROBLEM", "DORSOPATH", "SCOLIOSIS", "KYPHO", "SPONDY", "SPINAL DEGENERATION", "DEGENERATIVE DISC", "DISC PROLAPSE", "BACK INJURY", "CANAL STENOSIS", "CORD STENOSIS", "LBP", "LORDOSIS", "DDD" |  |
| Pain | Pain n.o.s. | Pain | "PAIN", "POLYMYALGIA", "PMR", "RHEUMATICA", "FIBROMYALGIA", "NEURALGIA", "SCIATICA" "HEADACHE" |  |
|  |  |  |  |  |
| Diseases of the visual system |  |  |  |  |
|  | Visual impairment | Poor vision (low vision both eyes, one eye, unspecified visual loss); Poor vision e.g. low vision both eyes, one eye, unspecified visual loss; Blindness (both eyes, one eye, one eye and low vision in other eye); Blindness e.g. both eyes, one eye, one eye and low vision in other eye | "VISUAL IMPAIRMENT", " VISION", "VISION", "EYESIGHT", "BLIND", "DIPLOPIA", "HEMIANOPIA" |  |
|  | Cataracts | Cataracts | "CATARACT", "CATERACT" |  |
|  | Retinopathies |  | "RETINOPATH", "MACULAR DEGEN", "ARMD", "RETINAL DETACH", "RETINAL H", "RETINITIS" |  |
|  | Glaucoma | Glaucoma | "GLAUCOMA", "GLUCOMA", "GLACOMA" | S01E |
|  | Dry eyes and other eye issues | Other diseases of the eye and adnexa n.o.s or n.e.c (includes conjunctivitis) | "DRY EYE" | S01XA20 |
|  |  |  |  |  |
| Diseases of the ear |  |  |  |  |
|  | Hearing impairment | Deafness/ hearing loss | "HEARING", "DEAF" |  |
|  |  |  |  |  |
| Skin | Other skin conditions | Other diseases of the skin and subcutaneous tissue n.o.s. or n.e.c. (includes bedsore, urticaria, erythema, radiation-related disorders, disorders of skin appendages); Rash and other nonspecific skin eruption | " RASH" |  |
|  | Ulcers, sores, wounds |  | "WOUND", "PRESSURE AREA", "LEG ULCER", "ULCER ANKLE", "HEEL ULCER", "SKIN TEAR" |  |
|  | Psoriasis, eczema, skin allergies | Skin allergies (dermatitis and eczema) | "SKIN ALLERGIES", "DERMATITIS", "ECZEMA", "PSORIASIS", "PSORASIS" | D05A, D07A, |
|  | Skin infections | Skin and subcutaneous tissue infections (e.g. impetigo, boil, cellulitis); Skin and subcutaneous tissue infections (includes impetigo, boil, cellulitis) | "CELLULITIS", "TISSUE INFECTION", "SKIN INFECTION" |  |

Conditions criteria were developed by a team composed of a pharmacist, a physiotherapist specializing in geriatric care, and a chronic disease epidemiologist. ACFI conditions are standardized and developed by the Australian government. Text strings were selected by reviewing unique text strings that appeared in the residents’ EHR notes, and then categorising the strings into appropriate categories. Text strings were matched using fuzzy matching in SAS using “like” function (e.g. *where text like ‘%OSTEOP%’;*). Most text strings allow any other text strings to precede or follow the string of interest, except where noted above with spaces or punctuation placed before or after the string (e.g. ‘ OA’ in the table indicates that a space must occur before ‘OA’). ATC codes were matched to medication administration records.

Supplemental Table 2. RACF population in Australia 2017

| **Age group** | **Females** | **Males** | **Total people** |
| --- | --- | --- | --- |
| 0-49 | 253 | 316 | 569 |
| 50-54 | 359 | 401 | 760 |
| 55-59 | 780 | 914 | 1694 |
| 60-64 | 1554 | 1860 | 3414 |
| 65-69 | 3152 | 3522 | 6674 |
| 70-74 | 6033 | 5698 | 11731 |
| 75-79 | 11078 | 8062 | 19140 |
| 80-84 | 20549 | 11162 | 31711 |
| 85-89 | 33431 | 14304 | 47735 |
| 90-94 | 31861 | 10463 | 42324 |
| 95-99 | 12713 | 3128 | 15841 |
| 100+ | 2138 | 343 | 2481 |
| Total | **123901** | **60173** | **184074** |
| Total in NSW |  |  | **62891** |
| Total in ACT |  |  | **2357** |

NSW is New South Wales. ACT is the Australian Capital Territory.

Data are from Aged Care GEN data 2017 which are publicly available at: https://gen-agedcaredata.gov.au/Resources/Access-data

Supplemental Table 3. Sample characteristics (n=9436)

|  | n | % | Australian RACF population | |
| --- | --- | --- | --- | --- |
| Age |  |  |  | |
| < 60 | 43 | 0.46% | 1.64% | |
| 60-64 | 86 | 0.91% | 1.85% | |
| 65-69 | 279 | 2.96% | 3.63% | |
| 70-74 | 576 | 6.10% | 6.37% | |
| 75-79 | 838 | 8.88% | 10.40% | |
| 80-84 | 1445 | 15.31% | 17.23% | |
| 85-89 | 2295 | 24.32% | 25.93% | |
| 90-94 | 2452 | 25.99% | 22.99% | |
| 95-99 | 1159 | 12.28% | 8.61% | |
| 100+ | 263 | 2.79% | 1.35% | |
| Females | 6340 | 67.19% | 67.31% | |
| Males | 3096 | 32.81% | 32.69% | |
| Died during follow up period | 4498 | 47.67% |  | |
| Discharged alive | 646 | 6.85% |  | |
| Facility remoteness |  |  |  | |
| Major city | 6777 | 71.82% |  | |
| Inner regional | 2453 | 26.00% |  | |
| Outer regional | 206 | 2.18% |  | |
|  |  |  |  | |
|  | Mean (SD) | Median (IQR) |  | |
| Length of Stay |  |  |  | |
| Among residents discharged | 3.36 (3.06) | 2.57 (0.93-4.97) | |  |
| Among residents with right censored follow up | 3.75 (3.54) | 2.73 (1.25-5.20) | |  |

RACF stays are right censored (i.e. end point not observed) for residents who were still in the facility as of 28 September 2017 when the study period ended. Age is age in last year of observation, which varies depending on when/if the resident was discharged from the facility. Percentages of residents in Australia are provided for comparison and based on the data in Supplemental Table 2.

Supplemental Table 4. Age and sex-specific estimates of condition prevalence (proportions) with 95% confidence intervals

|  |  | Age | | | | | | | | | |
| --- | --- | --- | --- | --- | --- | --- | --- | --- | --- | --- | --- |
| Circulatory Disease |  | 60 | 65 | 70 | 75 | 80 | 85 | 90 | 95 | 100 | 105 |
| Any Circulatory disease | F | 0.72 (0.64, 0.79) | 0.81 (0.76, 0.85) | 0.87 (0.84, 0.89) | 0.90 (0.89, 0.92) | 0.93 (0.91, 0.94) | 0.94 (0.93, 0.95) | 0.95 (0.94, 0.95) | 0.95 (0.94, 0.96) | 0.94 (0.93, 0.96) | 0.93 (0.91, 0.96) |
|  | M | 0.77 (0.69, 0.84) | 0.83 (0.79, 0.87) | 0.88 (0.86, 0.90) | 0.91 (0.90, 0.93) | 0.93 (0.92, 0.95) | 0.95 (0.94, 0.96) | 0.96 (0.95, 0.97) | 0.96 (0.95, 0.97) | 0.96 (0.94, 0.98) | 0.96 (0.93, 0.99) |
|  |  |  |  |  |  |  |  |  |  |  |  |
| Heart disease | F | 0.07 (0.04, 0.11) | 0.12 (0.08, 0.15) | 0.17 (0.14, 0.20) | 0.22 (0.20, 0.25) | 0.27 (0.25, 0.30) | 0.32 (0.30, 0.34) | 0.35 (0.33, 0.36) | 0.36 (0.34, 0.38) | 0.36 (0.33, 0.40) | 0.35 (0.30, 0.41) |
|  | M | 0.17 (0.10, 0.23) | 0.22 (0.17, 0.27) | 0.28 (0.24, 0.31) | 0.33 (0.30, 0.36) | 0.38 (0.35, 0.40) | 0.42 (0.39, 0.44) | 0.45 (0.42, 0.48) | 0.47 (0.44, 0.51) | 0.49 (0.42, 0.55) | 0.49 (0.39, 0.60) |
|  |  |  |  |  |  |  |  |  |  |  |  |
| Hypertension | F | 0.29 (0.21, 0.36) | 0.40 (0.34, 0.45) | 0.50 (0.46, 0.53) | 0.57 (0.55, 0.60) | 0.63 (0.60, 0.65) | 0.66 (0.64, 0.68) | 0.67 (0.65, 0.69) | 0.66 (0.64, 0.68) | 0.63 (0.59, 0.66) | 0.57 (0.52, 0.63) |
|  | M | 0.33 (0.25, 0.41) | 0.43 (0.37, 0.48) | 0.50 (0.47, 0.54) | 0.56 (0.53, 0.59) | 0.60 (0.57, 0.63) | 0.62 (0.59, 0.64) | 0.62 (0.59, 0.64) | 0.59 (0.56, 0.63) | 0.55 (0.49, 0.61) | 0.49 (0.38, 0.59) |
|  |  |  |  |  |  |  |  |  |  |  |  |
| Heart failure | F | 0.03 (0.01, 0.05) | 0.04 (0.02, 0.06) | 0.06 (0.04, 0.07) | 0.07 (0.06, 0.09) | 0.09 (0.08, 0.11) | 0.11 (0.10, 0.13) | 0.13 (0.12, 0.15) | 0.15 (0.14, 0.17) | 0.17 (0.14, 0.20) | 0.18 (0.14, 0.23) |
|  | M | 0.04 (0.01, 0.07) | 0.05 (0.03, 0.08) | 0.07 (0.05, 0.09) | 0.09 (0.07, 0.10) | 0.11 (0.09, 0.12) | 0.13 (0.11, 0.14) | 0.15 (0.13, 0.17) | 0.17 (0.14, 0.20) | 0.19 (0.13, 0.24) | 0.21 (0.11, 0.30) |
|  |  |  |  |  |  |  |  |  |  |  |  |
| Arrhythmias | F | 0.05 (0.02, 0.08) | 0.09 (0.06, 0.12) | 0.13 (0.10, 0.16) | 0.17 (0.15, 0.20) | 0.22 (0.20, 0.24) | 0.25 (0.23, 0.27) | 0.27 (0.26, 0.29) | 0.29 (0.27, 0.31) | 0.29 (0.25, 0.32) | 0.27 (0.22, 0.32) |
|  | M | 0.09 (0.04, 0.13) | 0.13 (0.10, 0.17) | 0.19 (0.16, 0.22) | 0.24 (0.21, 0.26) | 0.28 (0.26, 0.31) | 0.32 (0.29, 0.35) | 0.35 (0.32, 0.37) | 0.36 (0.32, 0.40) | 0.36 (0.30, 0.43) | 0.35 (0.25, 0.46) |
|  |  |  |  |  |  |  |  |  |  |  |  |
| Myocardial Infarction (MI) | F | 0.01 (0.00, 0.03) | 0.02 (0.00, 0.03) | 0.02 (0.01, 0.04) | 0.03 (0.02, 0.04) | 0.04 (0.03, 0.05) | 0.04 (0.04, 0.05) | 0.05 (0.04, 0.05) | 0.05 (0.04, 0.06) | 0.05 (0.03, 0.06) | 0.04 (0.02, 0.06) |
|  | M | 0.04 (0.01, 0.08) | 0.06 (0.03, 0.08) | 0.07 (0.05, 0.08) | 0.07 (0.06, 0.09) | 0.08 (0.07, 0.09) | 0.08 (0.07, 0.10) | 0.08 (0.07, 0.10) | 0.08 (0.06, 0.10) | 0.07 (0.04, 0.11) | 0.07 (0.02, 0.11) |
|  |  |  |  |  |  |  |  |  |  |  |  |
| Dyslipidaemia | F | 0.35 (0.27, 0.42) | 0.43 (0.37, 0.48) | 0.49 (0.45, 0.53) | 0.52 (0.49, 0.55) | 0.52 (0.50, 0.54) | 0.49 (0.47, 0.52) | 0.44 (0.42, 0.46) | 0.36 (0.34, 0.38) | 0.26 (0.23, 0.29) | 0.16 (0.12, 0.20) |
|  | M | 0.29 (0.21, 0.37) | 0.39 (0.33, 0.44) | 0.46 (0.42, 0.50) | 0.51 (0.48, 0.54) | 0.53 (0.50, 0.56) | 0.52 (0.49, 0.55) | 0.48 (0.45, 0.51) | 0.41 (0.37, 0.45) | 0.32 (0.26, 0.38) | 0.21 (0.14, 0.29) |
|  |  |  |  |  |  |  |  |  |  |  |  |
| Cerebrovascular disease (including stroke) | F | 0.17 (0.11, 0.22) | 0.19 (0.15, 0.23) | 0.21 (0.18, 0.24) | 0.23 (0.21, 0.25) | 0.24 (0.22, 0.26) | 0.24 (0.22, 0.26) | 0.24 (0.22, 0.26) | 0.23 (0.21, 0.25) | 0.22 (0.19, 0.24) | 0.19 (0.15, 0.24) |
|  | M | 0.29 (0.22, 0.37) | 0.31 (0.26, 0.36) | 0.33 (0.29, 0.36) | 0.33 (0.30, 0.36) | 0.33 (0.30, 0.35) | 0.32 (0.29, 0.34) | 0.30 (0.27, 0.32) | 0.27 (0.24, 0.30) | 0.24 (0.19, 0.29) | 0.20 (0.13, 0.27) |
|  |  |  |  |  |  |  |  |  |  |  |  |
|  |  | Age | | | | | | | | | |
| Neoplasms |  | 60 | 65 | 70 | 75 | 80 | 85 | 90 | 95 | 100 | 105 |
| Any neoplasm | F | 0.16 (0.11, 0.22) | 0.18 (0.14, 0.22) | 0.19 (0.16, 0.22) | 0.20 (0.18, 0.22) | 0.21 (0.20, 0.23) | 0.22 (0.21, 0.24) | 0.23 (0.22, 0.24) | 0.23 (0.22, 0.25) | 0.24 (0.21, 0.27) | 0.24 (0.19, 0.28) |
|  | M | 0.09 (0.05, 0.14) | 0.14 (0.10, 0.18) | 0.20 (0.17, 0.23) | 0.25 (0.23, 0.28) | 0.30 (0.28, 0.32) | 0.33 (0.31, 0.36) | 0.35 (0.33, 0.38) | 0.36 (0.32, 0.39) | 0.35 (0.28, 0.41) | 0.32 (0.22, 0.42) |
|  |  |  |  |  |  |  |  |  |  |  |  |
| Lung cancer | F | 0.04 (0.01, 0.07) | 0.03 (0.01, 0.05) | 0.02 (0.01, 0.03) | 0.02 (0.01, 0.03) | 0.01 (0.01, 0.02) | 0.01 (0.01, 0.01) | 0.01 (0.00, 0.01) | 0.00 (0.00, 0.01) | 0.00 (0.00, 0.01) | 0.00 (0.00, 0.00) |
|  | M | 0.03 (0.00, 0.05) | 0.02 (0.01, 0.04) | 0.02 (0.01, 0.03) | 0.02 (0.01, 0.03) | 0.02 (0.01, 0.02) | 0.01 (0.01, 0.02) | 0.01 (0.01, 0.02) | 0.01 (0.00, 0.02) | 0.01 (0.00, 0.02) | 0.01 (0.00, 0.02) |
|  |  |  |  |  |  |  |  |  |  |  |  |
| Colorectal cancer | F | 0.01 (0.00, 0.03) | 0.02 (0.00, 0.03) | 0.02 (0.01, 0.03) | 0.02 (0.02, 0.03) | 0.03 (0.02, 0.03) | 0.03 (0.03, 0.04) | 0.04 (0.03, 0.04) | 0.04 (0.03, 0.05) | 0.04 (0.03, 0.06) | 0.05 (0.02, 0.07) |
|  | M | 0.01 (0.00, 0.02) | 0.01 (0.00, 0.02) | 0.02 (0.01, 0.03) | 0.03 (0.02, 0.04) | 0.04 (0.03, 0.05) | 0.05 (0.04, 0.06) | 0.05 (0.04, 0.06) | 0.05 (0.03, 0.06) | 0.04 (0.01, 0.07) | 0.03 (0.00, 0.06) |
|  |  |  |  |  |  |  |  |  |  |  |  |
| Skin cancer | F | 0.02 (0.00, 0.04) | 0.03 (0.01, 0.04) | 0.04 (0.02, 0.05) | 0.05 (0.03, 0.06) | 0.06 (0.05, 0.07) | 0.07 (0.06, 0.08) | 0.09 (0.08, 0.10) | 0.10 (0.09, 0.12) | 0.12 (0.10, 0.14) | 0.14 (0.09, 0.18) |
|  | M | 0.04 (0.01, 0.07) | 0.05 (0.03, 0.07) | 0.06 (0.04, 0.08) | 0.08 (0.06, 0.10) | 0.10 (0.08, 0.12) | 0.12 (0.10, 0.14) | 0.15 (0.13, 0.17) | 0.18 (0.14, 0.21) | 0.21 (0.15, 0.26) | 0.24 (0.14, 0.34) |
|  |  |  |  |  |  |  |  |  |  |  |  |
| Metastatic cancer | F | 0.03 (0.00, 0.06) | 0.03 (0.01, 0.04) | 0.02 (0.01, 0.03) | 0.02 (0.01, 0.03) | 0.01 (0.01, 0.02) | 0.01 (0.01, 0.01) | 0.01 (0.00, 0.01) | 0.00 (0.00, 0.01) | 0.00 (0.00, 0.00) | 0.00 (0.00, 0.00) |
|  | M | 0.03 (0.00, 0.06) | 0.03 (0.01, 0.05) | 0.03 (0.02, 0.05) | 0.03 (0.02, 0.04) | 0.03 (0.02, 0.04) | 0.03 (0.02, 0.04) | 0.03 (0.02, 0.03) | 0.02 (0.01, 0.03) | 0.02 (0.00, 0.03) | 0.01 (0.00, 0.03) |
|  |  |  |  |  |  |  |  |  |  |  |  |
| Breast cancer* | F | 0.02 (0.00, 0.06) | 0.05 (0.01, 0.09) | 0.07 (0.04, 0.10) | 0.07 (0.04, 0.09) | 0.05 (0.04, 0.07) | 0.07 (0.05, 0.08) | 0.07 (0.05, 0.08) | 0.06 (0.04, 0.08) | 0.06 (0.03, 0.10) |  |
|  |  |  |  |  |  |  |  |  |  |  |  |
| Prostate cancer* | M |  | 0.04 (0.01, 0.07) | 0.05 (0.02, 0.07) | 0.12 (0.09, 0.15) | 0.12 (0.09, 0.15) | 0.12 (0.09, 0.14) | 0.13 (0.10, 0.15) | 0.09 (0.05, 0.13) | 0.11 (0.00, 0.22) |  |
|  |  |  |  |  |  |  |  |  |  |  |  |
| Leukemia or Lymphoma* | F |  |  | 0.00 (0.00, 0.01) | 0.02 (0.01, 0.04) | 0.02 (0.01, 0.02) | 0.01 (0.01, 0.02) | 0.02 (0.01, 0.02) | 0.01 (0.00, 0.02) | 0.01 (0.00, 0.02) |  |
|  | M | 0.03 (0.00, 0.08) | 0.01 (0.00, 0.02) | 0.03 (0.01, 0.05) | 0.02 (0.00, 0.03) | 0.02 (0.01, 0.04) | 0.02 (0.01, 0.03) | 0.02 (0.01, 0.03) | 0.02 (0.00, 0.04) | 0.04 (0.00, 0.10) |  |
|  |  |  |  |  |  |  |  |  |  |  |  |
|  |  |  |  |  |  |  |  |  |  |  |  |
|  |  | Age | | | | | | | | | |
| Respiratory |  | 60 | 65 | 70 | 75 | 80 | 85 | 90 | 95 | 100 | 105 |
| Chronic lower respiratory disease | F | 0.42 (0.34, 0.50) | 0.41 (0.35, 0.46) | 0.39 (0.36, 0.43) | 0.38 (0.35, 0.41) | 0.37 (0.34, 0.39) | 0.35 (0.33, 0.38) | 0.34 (0.32, 0.36) | 0.33 (0.31, 0.35) | 0.32 (0.28, 0.35) | 0.30 (0.25, 0.35) |
|  | M | 0.34 (0.27, 0.42) | 0.34 (0.29, 0.39) | 0.34 (0.30, 0.37) | 0.34 (0.31, 0.37) | 0.35 (0.32, 0.37) | 0.36 (0.33, 0.38) | 0.37 (0.34, 0.39) | 0.38 (0.34, 0.42) | 0.40 (0.34, 0.46) | 0.42 (0.32, 0.51) |
|  |  |  |  |  |  |  |  |  |  |  |  |
|  |  | Age | | | | | | | | | |
| Endocrine |  | 60 | 65 | 70 | 75 | 80 | 85 | 90 | 95 | 100 | 105 |
| Any endocrine disorder | F | 0.40 (0.32, 0.47) | 0.43 (0.38, 0.48) | 0.45 (0.41, 0.48) | 0.45 (0.43, 0.48) | 0.44 (0.42, 0.47) | 0.43 (0.41, 0.44) | 0.40 (0.38, 0.41) | 0.35 (0.33, 0.37) | 0.30 (0.27, 0.33) | 0.25 (0.20, 0.29) |
|  | M | 0.29 (0.21, 0.36) | 0.34 (0.29, 0.40) | 0.38 (0.35, 0.42) | 0.40 (0.37, 0.43) | 0.40 (0.37, 0.42) | 0.37 (0.35, 0.40) | 0.33 (0.31, 0.35) | 0.27 (0.24, 0.30) | 0.20 (0.15, 0.25) | 0.13 (0.07, 0.19) |
|  |  |  |  |  |  |  |  |  |  |  |  |
| Diabetes | F | 0.30 (0.23, 0.37) | 0.32 (0.27, 0.37) | 0.32 (0.29, 0.36) | 0.32 (0.29, 0.34) | 0.30 (0.28, 0.32) | 0.27 (0.25, 0.29) | 0.23 (0.21, 0.24) | 0.18 (0.17, 0.20) | 0.13 (0.11, 0.16) | 0.09 (0.06, 0.12) |
|  | M | 0.24 (0.17, 0.31) | 0.30 (0.24, 0.35) | 0.33 (0.30, 0.37) | 0.35 (0.32, 0.38) | 0.34 (0.31, 0.36) | 0.30 (0.28, 0.33) | 0.25 (0.23, 0.27) | 0.19 (0.16, 0.21) | 0.12 (0.08, 0.15) | 0.06 (0.03, 0.10) |
|  |  |  |  |  |  |  |  |  |  |  |  |
| Disorders of the thyroid gland | F | 0.15 (0.09, 0.20) | 0.17 (0.13, 0.21) | 0.18 (0.16, 0.21) | 0.20 (0.18, 0.22) | 0.21 (0.19, 0.22) | 0.21 (0.19, 0.22) | 0.21 (0.19, 0.22) | 0.20 (0.18, 0.21) | 0.19 (0.16, 0.21) | 0.17 (0.13, 0.21) |
|  | M | 0.06 (0.02, 0.10) | 0.07 (0.04, 0.10) | 0.07 (0.06, 0.09) | 0.08 (0.06, 0.09) | 0.08 (0.07, 0.10) | 0.09 (0.08, 0.10) | 0.09 (0.08, 0.10) | 0.09 (0.07, 0.12) | 0.10 (0.06, 0.13) | 0.10 (0.04, 0.16) |
|  |  |  |  |  |  |  |  |  |  |  |  |
|  |  | Age | | | | | | | | | |
| Nervous system |  | 60 | 65 | 70 | 75 | 80 | 85 | 90 | 95 | 100 | 105 |
| Any disease of the nervous system | F | 0.42 (0.34, 0.50) | 0.37 (0.32, 0.42) | 0.32 (0.28, 0.35) | 0.27 (0.24, 0.29) | 0.22 (0.20, 0.24) | 0.18 (0.16, 0.19) | 0.14 (0.12, 0.15) | 0.10 (0.09, 0.12) | 0.08 (0.06, 0.09) | 0.05 (0.03, 0.07) |
|  | M | 0.53 (0.45, 0.62) | 0.48 (0.42, 0.53) | 0.42 (0.38, 0.45) | 0.36 (0.33, 0.39) | 0.30 (0.27, 0.32) | 0.24 (0.22, 0.27) | 0.19 (0.17, 0.21) | 0.15 (0.12, 0.17) | 0.11 (0.07, 0.14) | 0.08 (0.04, 0.12) |
|  |  |  |  |  |  |  |  |  |  |  |  |
| Parkinson's disease | F | 0.05 (0.01, 0.08) | 0.06 (0.04, 0.09) | 0.08 (0.06, 0.10) | 0.08 (0.07, 0.10) | 0.08 (0.07, 0.09) | 0.07 (0.06, 0.08) | 0.06 (0.05, 0.07) | 0.04 (0.03, 0.05) | 0.02 (0.02, 0.03) | 0.01 (0.00, 0.02) |
|  | M | 0.04 (0.01, 0.07) | 0.08 (0.05, 0.11) | 0.12 (0.09, 0.15) | 0.15 (0.13, 0.17) | 0.15 (0.13, 0.18) | 0.14 (0.12, 0.16) | 0.10 (0.09, 0.12) | 0.06 (0.04, 0.08) | 0.03 (0.01, 0.04) | 0.01 (0.00, 0.02) |
|  |  |  |  |  |  |  |  |  |  |  |  |
| Multiple sclerosis | F | 0.03 (0.00, 0.06) | 0.02 (0.01, 0.04) | 0.02 (0.01, 0.03) | 0.01 (0.00, 0.02) | 0.01 (0.00, 0.01) | 0.00 (0.00, 0.00) | 0.00 (0.00, 0.00) | 0.00 (0.00, 0.00) | 0.00 (0.00, 0.00) | 0.00 (0.00, 0.00) |
|  | M | 0.05 (0.01, 0.08) | 0.02 (0.00, 0.04) | 0.01 (0.00, 0.02) | 0.00 (0.00, 0.01) | 0.00 (0.00, 0.00) | 0.00 (0.00, 0.00) | 0.00 (0.00, 0.00) | 0.00 (0.00, 0.00) | 0.00 (0.00, 0.00) | 0.00 (0.00, 0.00) |
|  |  |  |  |  |  |  |  |  |  |  |  |
| Epilepsy | F | 0.19 (0.12, 0.26) | 0.14 (0.11, 0.18) | 0.11 (0.08, 0.13) | 0.08 (0.06, 0.09) | 0.05 (0.05, 0.06) | 0.04 (0.03, 0.04) | 0.03 (0.02, 0.03) | 0.02 (0.01, 0.02) | 0.01 (0.01, 0.02) | 0.01 (0.00, 0.01) |
|  | M | 0.19 (0.12, 0.25) | 0.16 (0.12, 0.20) | 0.13 (0.10, 0.15) | 0.10 (0.08, 0.11) | 0.07 (0.06, 0.08) | 0.05 (0.04, 0.06) | 0.03 (0.02, 0.04) | 0.02 (0.01, 0.02) | 0.01 (0.00, 0.02) | 0.00 (0.00, 0.01) |
|  |  |  |  |  |  |  |  |  |  |  |  |
| Brain injury | F | 0.03 (0.00, 0.06) | 0.02 (0.01, 0.03) | 0.01 (0.01, 0.02) | 0.01 (0.00, 0.01) | 0.01 (0.00, 0.01) | 0.00 (0.00, 0.00) | 0.00 (0.00, 0.00) | 0.00 (0.00, 0.00) | 0.00 (0.00, 0.00) | 0.00 (0.00, 0.00) |
|  | M | 0.12 (0.07, 0.18) | 0.07 (0.04, 0.10) | 0.04 (0.02, 0.05) | 0.02 (0.01, 0.03) | 0.01 (0.00, 0.01) | 0.00 (0.00, 0.01) | 0.00 (0.00, 0.00) | 0.00 (0.00, 0.00) | 0.00 (0.00, 0.00) | 0.00 (0.00, 0.00) |
|  |  |  |  |  |  |  |  |  |  |  |  |
| Motor Neurone Disease* | F |  |  | 0.01 (0.00, 0.02) | 0.00 (0.00, 0.01) | 0.00 (0.00, 0.00) | 0.00 (0.00, 0.00) | 0.00 (0.00, 0.00) | 0.00 (0.00, 0.00) |  |  |
|  | M |  | 0.01 (0.00, 0.03) | 0.01 (0.00, 0.02) |  |  | 0.00 (0.00, 0.01) |  |  |  |  |
|  |  |  |  |  |  |  |  |  |  |  |  |
|  |  | 45 | 50 | 55 | 60 | 65 | 70 | 75 | 80 | 85 | 90 |
| Huntington's disease | F | 0.01 (-0.05, 0.07) | 0.01 (-0.04, 0.06) | 0.01 (-0.02, 0.05) | 0.01 (-0.01, 0.03) | 0.01 (0.00, 0.02) | 0.01 (0.00, 0.01) | 0.00 (0.00, 0.01) | 0.00 (0.00, 0.00) | 0.00 (0.00, 0.00) | 0.00 (0.00, 0.00) |
|  | M | 0.10 (-0.09, 0.28) | 0.06 (-0.03, 0.16) | 0.04 (-0.01, 0.08) | 0.02 (0.00, 0.04) | 0.01 (0.00, 0.02) | 0.01 (0.00, 0.01) | 0.00 (0.00, 0.01) | 0.00 (0.00, 0.00) | 0.00 (0.00, 0.00) | 0.00 (0.00, 0.00) |
|  |  |  |  |  |  |  |  |  |  |  |  |
|  |  | Age | | | | | | | | | |
| Digestive |  | 60 | 65 | 70 | 75 | 80 | 85 | 90 | 95 | 100 | 105 |
| Other digestive (including inflammatory) conditions | F | 0.13 (0.08, 0.19) | 0.16 (0.12, 0.20) | 0.19 (0.15, 0.22) | 0.21 (0.18, 0.23) | 0.22 (0.20, 0.25) | 0.23 (0.21, 0.25) | 0.23 (0.21, 0.25) | 0.23 (0.20, 0.25) | 0.21 (0.18, 0.24) | 0.19 (0.15, 0.24) |
|  | M | 0.14 (0.08, 0.19) | 0.15 (0.11, 0.19) | 0.16 (0.13, 0.19) | 0.18 (0.15, 0.20) | 0.20 (0.17, 0.22) | 0.22 (0.19, 0.24) | 0.24 (0.22, 0.27) | 0.27 (0.23, 0.31) | 0.30 (0.24, 0.36) | 0.33 (0.23, 0.43) |
|  |  |  |  |  |  |  |  |  |  |  |  |
| Liver disease | F | 0.09 (0.04, 0.14) | 0.06 (0.04, 0.09) | 0.05 (0.03, 0.06) | 0.03 (0.02, 0.04) | 0.02 (0.02, 0.03) | 0.02 (0.01, 0.02) | 0.01 (0.01, 0.02) | 0.01 (0.01, 0.01) | 0.01 (0.00, 0.01) | 0.01 (0.00, 0.01) |
|  | M | 0.14 (0.08, 0.19) | 0.09 (0.07, 0.12) | 0.07 (0.05, 0.08) | 0.05 (0.03, 0.06) | 0.03 (0.02, 0.04) | 0.02 (0.02, 0.03) | 0.02 (0.01, 0.02) | 0.01 (0.00, 0.02) | 0.01 (0.00, 0.02) | 0.01 (0.00, 0.02) |
|  |  |  |  |  |  |  |  |  |  |  |  |
| PUD, GORD and other acid related conditions | F | 0.48 (0.40, 0.56) | 0.52 (0.47, 0.57) | 0.55 (0.51, 0.59) | 0.57 (0.55, 0.60) | 0.59 (0.57, 0.61) | 0.60 (0.57, 0.62) | 0.60 (0.58, 0.62) | 0.59 (0.57, 0.61) | 0.57 (0.54, 0.61) | 0.55 (0.49, 0.60) |
|  | M | 0.44 (0.36, 0.52) | 0.48 (0.42, 0.53) | 0.51 (0.47, 0.55) | 0.54 (0.51, 0.57) | 0.56 (0.53, 0.59) | 0.58 (0.55, 0.60) | 0.59 (0.57, 0.62) | 0.60 (0.56, 0.64) | 0.61 (0.55, 0.67) | 0.61 (0.51, 0.70) |
|  |  |  |  |  |  |  |  |  |  |  |  |
| Diarrhoea | F | 0.01 (0.00, 0.02) | 0.01 (0.00, 0.02) | 0.01 (0.00, 0.02) | 0.02 (0.01, 0.02) | 0.02 (0.01, 0.03) | 0.02 (0.01, 0.03) | 0.02 (0.01, 0.02) | 0.02 (0.01, 0.02) | 0.01 (0.00, 0.02) | 0.01 (0.00, 0.02) |
|  | M | 0.01 (0.00, 0.03) | 0.01 (0.00, 0.02) | 0.01 (0.00, 0.02) | 0.01 (0.01, 0.02) | 0.01 (0.01, 0.02) | 0.01 (0.01, 0.02) | 0.02 (0.01, 0.02) | 0.02 (0.01, 0.03) | 0.03 (0.01, 0.05) | 0.04 (-0.01, 0.08) |
|  |  |  |  |  |  |  |  |  |  |  |  |
| Constipation | F | 0.76 (0.69, 0.83) | 0.74 (0.69, 0.79) | 0.73 (0.69, 0.76) | 0.72 (0.69, 0.75) | 0.72 (0.70, 0.75) | 0.74 (0.71, 0.76) | 0.76 (0.74, 0.78) | 0.79 (0.77, 0.81) | 0.83 (0.80, 0.85) | 0.86 (0.83, 0.90) |
|  | M | 0.67 (0.59, 0.75) | 0.68 (0.62, 0.73) | 0.68 (0.65, 0.72) | 0.70 (0.66, 0.73) | 0.71 (0.68, 0.74) | 0.73 (0.70, 0.75) | 0.74 (0.71, 0.77) | 0.76 (0.73, 0.80) | 0.79 (0.73, 0.84) | 0.81 (0.74, 0.88) |
|  |  |  |  |  |  |  |  |  |  |  |  |
| Nausea or vomiting | F | 0.22 (0.15, 0.28) | 0.21 (0.17, 0.26) | 0.21 (0.17, 0.24) | 0.21 (0.18, 0.23) | 0.21 (0.18, 0.23) | 0.21 (0.19, 0.23) | 0.22 (0.19, 0.24) | 0.22 (0.20, 0.25) | 0.23 (0.20, 0.26) | 0.24 (0.19, 0.29) |
|  | M | 0.20 (0.14, 0.26) | 0.16 (0.12, 0.20) | 0.14 (0.11, 0.16) | 0.12 (0.10, 0.15) | 0.12 (0.10, 0.14) | 0.13 (0.11, 0.15) | 0.14 (0.12, 0.16) | 0.16 (0.13, 0.19) | 0.20 (0.15, 0.26) | 0.26 (0.17, 0.35) |
|  |  |  |  |  |  |  |  |  |  |  |  |
| Faecal incontinence | F | 0.24 (0.17, 0.32) | 0.24 (0.18, 0.30) | 0.23 (0.19, 0.28) | 0.23 (0.19, 0.27) | 0.22 (0.18, 0.26) | 0.22 (0.18, 0.26) | 0.21 (0.17, 0.25) | 0.20 (0.16, 0.24) | 0.20 (0.15, 0.24) | 0.19 (0.14, 0.24) |
|  | M | 0.22 (0.15, 0.29) | 0.23 (0.18, 0.29) | 0.23 (0.19, 0.28) | 0.24 (0.19, 0.28) | 0.23 (0.19, 0.27) | 0.22 (0.18, 0.27) | 0.21 (0.17, 0.25) | 0.20 (0.16, 0.24) | 0.18 (0.13, 0.23) | 0.16 (0.10, 0.23) |
|  |  |  |  |  |  |  |  |  |  |  |  |
| Double incontinence | F | 0.19 (0.13, 0.26) | 0.19 (0.14, 0.24) | 0.18 (0.14, 0.22) | 0.18 (0.14, 0.21) | 0.17 (0.13, 0.21) | 0.16 (0.13, 0.20) | 0.16 (0.13, 0.19) | 0.16 (0.12, 0.19) | 0.15 (0.11, 0.19) | 0.15 (0.10, 0.19) |
|  | M | 0.15 (0.09, 0.21) | 0.16 (0.11, 0.21) | 0.17 (0.13, 0.21) | 0.17 (0.13, 0.21) | 0.17 (0.13, 0.20) | 0.16 (0.12, 0.19) | 0.15 (0.11, 0.18) | 0.13 (0.10, 0.17) | 0.11 (0.07, 0.16) | 0.10 (0.04, 0.15) |
|  |  |  |  |  |  |  |  |  |  |  |  |
|  |  | Age | | | | | | | | | |
| Diseases of the urinary system |  | 60 | 65 | 70 | 75 | 80 | 85 | 90 | 95 | 100 | 105 |
| Urinary incontinence | F | 0.51 (0.43, 0.60) | 0.51 (0.45, 0.58) | 0.52 (0.46, 0.57) | 0.52 (0.47, 0.57) | 0.52 (0.47, 0.57) | 0.52 (0.48, 0.57) | 0.53 (0.48, 0.57) | 0.53 (0.49, 0.58) | 0.54 (0.48, 0.59) | 0.54 (0.48, 0.61) |
|  | M | 0.39 (0.31, 0.48) | 0.41 (0.35, 0.47) | 0.43 (0.37, 0.48) | 0.44 (0.39, 0.48) | 0.44 (0.39, 0.49) | 0.44 (0.40, 0.49) | 0.44 (0.40, 0.49) | 0.44 (0.38, 0.49) | 0.43 (0.36, 0.50) | 0.41 (0.32, 0.51) |
|  |  |  |  |  |  |  |  |  |  |  |  |
| Renal disease | F | 0.10 (0.06, 0.15) | 0.12 (0.08, 0.15) | 0.12 (0.10, 0.15) | 0.13 (0.11, 0.15) | 0.13 (0.12, 0.15) | 0.14 (0.12, 0.15) | 0.13 (0.12, 0.14) | 0.13 (0.12, 0.14) | 0.12 (0.10, 0.14) | 0.11 (0.08, 0.15) |
|  | M | 0.06 (0.02, 0.10) | 0.09 (0.06, 0.12) | 0.13 (0.10, 0.15) | 0.16 (0.14, 0.18) | 0.18 (0.16, 0.20) | 0.20 (0.18, 0.22) | 0.21 (0.19, 0.23) | 0.21 (0.18, 0.24) | 0.20 (0.15, 0.25) | 0.18 (0.10, 0.25) |
|  |  |  |  |  |  |  |  |  |  |  |  |
| UTI | F | 0.09 (0.05, 0.13) | 0.11 (0.08, 0.14) | 0.13 (0.10, 0.15) | 0.15 (0.13, 0.17) | 0.16 (0.14, 0.18) | 0.18 (0.16, 0.20) | 0.19 (0.17, 0.21) | 0.20 (0.18, 0.22) | 0.21 (0.18, 0.24) | 0.21 (0.17, 0.26) |
|  | M | 0.04 (0.01, 0.07) | 0.05 (0.03, 0.08) | 0.07 (0.05, 0.08) | 0.07 (0.06, 0.09) | 0.08 (0.06, 0.10) | 0.08 (0.07, 0.10) | 0.08 (0.07, 0.10) | 0.07 (0.05, 0.10) | 0.07 (0.04, 0.10) | 0.06 (0.01, 0.10) |
|  |  |  |  |  |  |  |  |  |  |  |  |
|  |  | Age | | | | | | | | | |
| Mental & behavioural disorders |  | 60 | 65 | 70 | 75 | 80 | 85 | 90 | 95 | 100 | 105 |
| Any mental or behavioural disorder | F | 0.93 (0.89, 0.97) | 0.94 (0.92, 0.97) | 0.95 (0.93, 0.96) | 0.95 (0.93, 0.96) | 0.95 (0.93, 0.96) | 0.94 (0.93, 0.96) | 0.93 (0.92, 0.95) | 0.92 (0.90, 0.94) | 0.90 (0.87, 0.92) | 0.86 (0.82, 0.91) |
|  | M | 0.90 (0.86, 0.95) | 0.92 (0.89, 0.95) | 0.93 (0.91, 0.95) | 0.93 (0.91, 0.95) | 0.93 (0.91, 0.95) | 0.92 (0.90, 0.94) | 0.90 (0.88, 0.92) | 0.88 (0.85, 0.91) | 0.83 (0.78, 0.88) | 0.77 (0.68, 0.86) |
|  |  |  |  |  |  |  |  |  |  |  |  |
| Other mental or behavioural disorder | F | 0.13 (0.07, 0.19) | 0.11 (0.08, 0.14) | 0.08 (0.06, 0.11) | 0.06 (0.05, 0.08) | 0.04 (0.03, 0.05) | 0.03 (0.02, 0.03) | 0.02 (0.01, 0.02) | 0.01 (0.00, 0.01) | 0.00 (0.00, 0.01) | 0.00 (0.00, 0.00) |
|  | M | 0.32 (0.24, 0.40) | 0.28 (0.23, 0.33) | 0.23 (0.20, 0.26) | 0.18 (0.15, 0.20) | 0.12 (0.10, 0.14) | 0.08 (0.06, 0.09) | 0.04 (0.03, 0.05) | 0.02 (0.01, 0.03) | 0.01 (0.00, 0.01) | 0.00 (0.00, 0.00) |
|  |  |  |  |  |  |  |  |  |  |  |  |
| Dementia | F | 0.37 (0.29, 0.45) | 0.44 (0.37, 0.51) | 0.51 (0.44, 0.57) | 0.55 (0.50, 0.61) | 0.59 (0.53, 0.64) | 0.61 (0.56, 0.66) | 0.62 (0.57, 0.67) | 0.61 (0.56, 0.67) | 0.60 (0.54, 0.65) | 0.56 (0.49, 0.63) |
|  | M | 0.25 (0.17, 0.33) | 0.37 (0.30, 0.44) | 0.47 (0.41, 0.54) | 0.55 (0.49, 0.61) | 0.59 (0.54, 0.65) | 0.61 (0.55, 0.66) | 0.59 (0.54, 0.65) | 0.55 (0.49, 0.61) | 0.47 (0.40, 0.55) | 0.37 (0.27, 0.47) |
|  |  |  |  |  |  |  |  |  |  |  |  |
| Cognitive impairment, memory loss | F | 0.18 (0.12, 0.24) | 0.20 (0.16, 0.25) | 0.22 (0.18, 0.26) | 0.24 (0.20, 0.27) | 0.25 (0.22, 0.28) | 0.26 (0.23, 0.29) | 0.27 (0.24, 0.30) | 0.28 (0.24, 0.31) | 0.28 (0.24, 0.32) | 0.28 (0.22, 0.33) |
|  | M | 0.28 (0.21, 0.36) | 0.28 (0.22, 0.33) | 0.27 (0.23, 0.31) | 0.27 (0.24, 0.31) | 0.27 (0.24, 0.31) | 0.28 (0.25, 0.31) | 0.29 (0.26, 0.32) | 0.30 (0.26, 0.35) | 0.32 (0.26, 0.38) | 0.34 (0.25, 0.43) |
|  |  |  |  |  |  |  |  |  |  |  |  |
| Schizophrenia, paranoid or psychotic states | F | 0.30 (0.22, 0.39) | 0.27 (0.22, 0.33) | 0.24 (0.20, 0.28) | 0.20 (0.16, 0.23) | 0.16 (0.13, 0.18) | 0.12 (0.10, 0.14) | 0.09 (0.07, 0.10) | 0.06 (0.04, 0.07) | 0.04 (0.02, 0.05) | 0.02 (0.01, 0.03) |
|  | M | 0.17 (0.10, 0.23) | 0.16 (0.12, 0.21) | 0.15 (0.12, 0.19) | 0.13 (0.11, 0.16) | 0.11 (0.09, 0.13) | 0.08 (0.06, 0.10) | 0.06 (0.04, 0.07) | 0.04 (0.02, 0.05) | 0.02 (0.01, 0.03) | 0.01 (0.00, 0.02) |
|  |  |  |  |  |  |  |  |  |  |  |  |
| Delirium | F | 0.01 (0.00, 0.02) | 0.01 (0.00, 0.03) | 0.03 (0.01, 0.04) | 0.05 (0.04, 0.06) | 0.05 (0.04, 0.06) | 0.06 (0.05, 0.07) | 0.07 (0.06, 0.08) | 0.07 (0.05, 0.08) | 0.06 (0.04, 0.07) | 0.05 (0.02, 0.07) |
|  | M | 0.01 (0.00, 0.02) | 0.02 (0.00, 0.03) | 0.03 (0.02, 0.04) | 0.05 (0.03, 0.06) | 0.06 (0.05, 0.07) | 0.07 (0.05, 0.08) | 0.07 (0.06, 0.08) | 0.06 (0.04, 0.08) | 0.05 (0.02, 0.08) | 0.03 (0.00, 0.07) |
|  |  |  |  |  |  |  |  |  |  |  |  |
| Depression | F | 0.65 (0.57, 0.73) | 0.65 (0.59, 0.70) | 0.64 (0.59, 0.68) | 0.62 (0.59, 0.66) | 0.60 (0.57, 0.64) | 0.58 (0.54, 0.61) | 0.54 (0.50, 0.58) | 0.50 (0.46, 0.54) | 0.45 (0.41, 0.50) | 0.40 (0.34, 0.46) |
|  | M | 0.57 (0.49, 0.66) | 0.58 (0.52, 0.64) | 0.57 (0.52, 0.62) | 0.56 (0.52, 0.60) | 0.54 (0.50, 0.58) | 0.51 (0.47, 0.55) | 0.48 (0.44, 0.52) | 0.44 (0.39, 0.48) | 0.39 (0.33, 0.45) | 0.34 (0.25, 0.42) |
|  |  |  |  |  |  |  |  |  |  |  |  |
| Anxiety & stress related disorders | F | 0.34 (0.26, 0.42) | 0.34 (0.29, 0.40) | 0.34 (0.30, 0.38) | 0.33 (0.30, 0.37) | 0.32 (0.29, 0.35) | 0.30 (0.27, 0.33) | 0.28 (0.25, 0.31) | 0.26 (0.23, 0.29) | 0.23 (0.19, 0.26) | 0.20 (0.16, 0.25) |
|  | M | 0.28 (0.21, 0.36) | 0.25 (0.20, 0.30) | 0.23 (0.19, 0.27) | 0.21 (0.18, 0.24) | 0.19 (0.17, 0.22) | 0.18 (0.15, 0.21) | 0.17 (0.14, 0.19) | 0.16 (0.13, 0.19) | 0.15 (0.11, 0.20) | 0.15 (0.08, 0.21) |
|  |  |  |  |  |  |  |  |  |  |  |  |
|  |  | Age | | | | | | | | | |
| Musculoskeletal and soft tissue |  | 60 | 65 | 70 | 75 | 80 | 85 | 90 | 95 | 100 | 105 |
| Other musculoskeletal or soft tissue disorder | F | 0.03 (0.01, 0.06) | 0.04 (0.02, 0.06) | 0.05 (0.03, 0.06) | 0.06 (0.04, 0.07) | 0.06 (0.05, 0.07) | 0.07 (0.06, 0.08) | 0.07 (0.06, 0.08) | 0.07 (0.06, 0.08) | 0.07 (0.05, 0.08) | 0.06 (0.04, 0.09) |
|  | M | 0.05 (0.01, 0.08) | 0.05 (0.03, 0.08) | 0.06 (0.04, 0.07) | 0.06 (0.05, 0.07) | 0.06 (0.05, 0.08) | 0.06 (0.05, 0.08) | 0.06 (0.05, 0.07) | 0.06 (0.04, 0.08) | 0.06 (0.03, 0.09) | 0.05 (0.01, 0.10) |
|  |  |  |  |  |  |  |  |  |  |  |  |
| Osteoporosis | F | 0.15 (0.10, 0.20) | 0.21 (0.16, 0.25) | 0.27 (0.23, 0.30) | 0.33 (0.30, 0.35) | 0.38 (0.35, 0.40) | 0.42 (0.40, 0.44) | 0.45 (0.43, 0.48) | 0.48 (0.45, 0.50) | 0.49 (0.45, 0.52) | 0.48 (0.43, 0.54) |
|  | M | 0.08 (0.04, 0.13) | 0.10 (0.07, 0.13) | 0.12 (0.09, 0.14) | 0.14 (0.12, 0.16) | 0.16 (0.14, 0.18) | 0.18 (0.16, 0.20) | 0.20 (0.17, 0.22) | 0.21 (0.18, 0.24) | 0.23 (0.17, 0.29) | 0.25 (0.15, 0.34) |
|  |  |  |  |  |  |  |  |  |  |  |  |
| Arthritis | F | 0.27 (0.20, 0.34) | 0.36 (0.31, 0.42) | 0.45 (0.41, 0.49) | 0.53 (0.50, 0.56) | 0.59 (0.56, 0.62) | 0.64 (0.61, 0.66) | 0.67 (0.65, 0.69) | 0.69 (0.67, 0.72) | 0.70 (0.66, 0.73) | 0.69 (0.64, 0.75) |
|  | M | 0.25 (0.17, 0.32) | 0.32 (0.27, 0.37) | 0.40 (0.36, 0.44) | 0.47 (0.43, 0.50) | 0.53 (0.50, 0.56) | 0.59 (0.56, 0.61) | 0.63 (0.60, 0.66) | 0.67 (0.63, 0.71) | 0.70 (0.64, 0.75) | 0.72 (0.63, 0.80) |
|  |  |  |  |  |  |  |  |  |  |  |  |
| Rheumatoid arthritis | F | 0.02 (0.00, 0.04) | 0.03 (0.01, 0.05) | 0.04 (0.02, 0.05) | 0.04 (0.03, 0.05) | 0.04 (0.04, 0.05) | 0.04 (0.03, 0.05) | 0.04 (0.03, 0.04) | 0.03 (0.02, 0.03) | 0.02 (0.01, 0.03) | 0.01 (0.00, 0.02) |
|  | M | 0.00 (0.00, 0.01) | 0.01 (0.00, 0.02) | 0.01 (0.00, 0.02) | 0.02 (0.01, 0.03) | 0.02 (0.02, 0.03) | 0.02 (0.02, 0.03) | 0.02 (0.02, 0.03) | 0.02 (0.01, 0.03) | 0.01 (0.00, 0.03) | 0.01 (-0.01, 0.02) |
|  |  |  |  |  |  |  |  |  |  |  |  |
| Gout | F | 0.02 (0.00, 0.04) | 0.03 (0.01, 0.05) | 0.05 (0.03, 0.07) | 0.07 (0.05, 0.08) | 0.08 (0.07, 0.09) | 0.09 (0.08, 0.10) | 0.09 (0.08, 0.10) | 0.08 (0.07, 0.09) | 0.07 (0.05, 0.09) | 0.05 (0.03, 0.08) |
|  | M | 0.07 (0.03, 0.11) | 0.09 (0.06, 0.13) | 0.12 (0.10, 0.15) | 0.15 (0.13, 0.17) | 0.17 (0.15, 0.19) | 0.19 (0.17, 0.21) | 0.20 (0.19, 0.22) | 0.21 (0.18, 0.24) | 0.21 (0.16, 0.26) | 0.20 (0.12, 0.29) |
|  |  |  |  |  |  |  |  |  |  |  |  |
| Fracture | F | 0.16 (0.10, 0.22) | 0.18 (0.14, 0.22) | 0.20 (0.17, 0.23) | 0.23 (0.20, 0.25) | 0.25 (0.23, 0.28) | 0.28 (0.26, 0.31) | 0.31 (0.29, 0.34) | 0.35 (0.32, 0.37) | 0.38 (0.35, 0.42) | 0.42 (0.36, 0.48) |
|  | M | 0.16 (0.10, 0.22) | 0.16 (0.12, 0.20) | 0.16 (0.13, 0.19) | 0.17 (0.14, 0.19) | 0.17 (0.15, 0.19) | 0.19 (0.16, 0.21) | 0.20 (0.18, 0.22) | 0.22 (0.19, 0.25) | 0.25 (0.20, 0.30) | 0.28 (0.19, 0.37) |
|  |  |  |  |  |  |  |  |  |  |  |  |
| Amputation (any) | F | 0.04 (0.01, 0.07) | 0.02 (0.01, 0.04) | 0.02 (0.01, 0.03) | 0.01 (0.01, 0.02) | 0.01 (0.01, 0.02) | 0.01 (0.01, 0.01) | 0.01 (0.01, 0.01) | 0.01 (0.01, 0.01) | 0.01 (0.00, 0.02) | 0.01 (0.00, 0.02) |
|  | M | 0.04 (0.01, 0.07) | 0.04 (0.02, 0.06) | 0.04 (0.02, 0.05) | 0.03 (0.02, 0.04) | 0.03 (0.02, 0.04) | 0.03 (0.02, 0.03) | 0.02 (0.01, 0.03) | 0.02 (0.01, 0.03) | 0.01 (0.00, 0.02) | 0.01 (0.00, 0.02) |
|  |  |  |  |  |  |  |  |  |  |  |  |
| Back pain | F | 0.04 (0.01, 0.06) | 0.05 (0.03, 0.07) | 0.07 (0.05, 0.09) | 0.08 (0.07, 0.10) | 0.10 (0.08, 0.11) | 0.10 (0.09, 0.12) | 0.11 (0.10, 0.12) | 0.11 (0.09, 0.12) | 0.10 (0.08, 0.12) | 0.09 (0.06, 0.12) |
|  | M | 0.05 (0.02, 0.08) | 0.06 (0.03, 0.08) | 0.06 (0.04, 0.08) | 0.07 (0.05, 0.08) | 0.07 (0.06, 0.08) | 0.08 (0.07, 0.09) | 0.08 (0.07, 0.10) | 0.09 (0.07, 0.11) | 0.10 (0.06, 0.14) | 0.11 (0.05, 0.18) |
|  |  |  |  |  |  |  |  |  |  |  |  |
|  |  | Age | | | | | | | | | |
| Pain |  | 60 | 65 | 70 | 75 | 80 | 85 | 90 | 95 | 100 | 105 |
| Pain & pain syndromes | F | 0.30 (0.22, 0.37) | 0.34 (0.28, 0.39) | 0.37 (0.33, 0.42) | 0.40 (0.36, 0.43) | 0.41 (0.38, 0.45) | 0.42 (0.38, 0.45) | 0.41 (0.38, 0.44) | 0.39 (0.36, 0.43) | 0.37 (0.33, 0.41) | 0.33 (0.28, 0.39) |
|  | M | 0.31 (0.24, 0.39) | 0.33 (0.27, 0.38) | 0.34 (0.29, 0.38) | 0.34 (0.31, 0.38) | 0.35 (0.31, 0.39) | 0.35 (0.32, 0.39) | 0.36 (0.32, 0.39) | 0.36 (0.31, 0.40) | 0.35 (0.29, 0.42) | 0.35 (0.26, 0.44) |
|  |  |  |  |  |  |  |  |  |  |  |  |
|  |  | Age | | | | | | | | | |
| Visual |  | 60 | 65 | 70 | 75 | 80 | 85 | 90 | 95 | 100 | 105 |
| Visual impairment | F | 0.16 (0.10, 0.21) | 0.12 (0.09, 0.16) | 0.11 (0.09, 0.13) | 0.10 (0.09, 0.12) | 0.11 (0.10, 0.13) | 0.14 (0.12, 0.15) | 0.18 (0.16, 0.19) | 0.25 (0.23, 0.27) | 0.36 (0.32, 0.39) | 0.50 (0.44, 0.56) |
|  | M | 0.12 (0.07, 0.17) | 0.11 (0.08, 0.14) | 0.10 (0.08, 0.12) | 0.11 (0.09, 0.12) | 0.12 (0.10, 0.14) | 0.14 (0.12, 0.16) | 0.18 (0.16, 0.19) | 0.23 (0.20, 0.26) | 0.30 (0.24, 0.36) | 0.40 (0.30, 0.50) |
|  |  |  |  |  |  |  |  |  |  |  |  |
| Cataracts | F | 0.03 (0.01, 0.06) | 0.05 (0.03, 0.08) | 0.08 (0.06, 0.10) | 0.11 (0.09, 0.13) | 0.14 (0.12, 0.15) | 0.16 (0.14, 0.17) | 0.17 (0.15, 0.19) | 0.17 (0.16, 0.19) | 0.17 (0.14, 0.20) | 0.15 (0.11, 0.20) |
|  | M | 0.02 (0.00, 0.03) | 0.03 (0.01, 0.05) | 0.05 (0.04, 0.07) | 0.08 (0.06, 0.09) | 0.10 (0.08, 0.12) | 0.12 (0.10, 0.14) | 0.13 (0.11, 0.15) | 0.13 (0.10, 0.16) | 0.12 (0.08, 0.17) | 0.10 (0.04, 0.17) |
|  |  |  |  |  |  |  |  |  |  |  |  |
| Retinopathy | F | 0.02 (0.00, 0.04) | 0.03 (0.01, 0.04) | 0.04 (0.02, 0.05) | 0.05 (0.04, 0.06) | 0.06 (0.05, 0.07) | 0.09 (0.08, 0.10) | 0.12 (0.11, 0.13) | 0.18 (0.16, 0.19) | 0.25 (0.22, 0.28) | 0.35 (0.29, 0.41) |
|  | M | 0.02 (0.00, 0.03) | 0.02 (0.01, 0.03) | 0.02 (0.01, 0.03) | 0.03 (0.02, 0.03) | 0.04 (0.03, 0.05) | 0.06 (0.05, 0.07) | 0.09 (0.08, 0.11) | 0.16 (0.13, 0.18) | 0.25 (0.19, 0.32) | 0.39 (0.27, 0.52) |
|  |  |  |  |  |  |  |  |  |  |  |  |
| Dry eyes and other eye issues | F | 0.20 (0.14, 0.26) | 0.21 (0.16, 0.25) | 0.22 (0.19, 0.25) | 0.24 (0.22, 0.27) | 0.27 (0.25, 0.30) | 0.31 (0.29, 0.34) | 0.36 (0.34, 0.39) | 0.42 (0.40, 0.45) | 0.50 (0.46, 0.53) | 0.58 (0.52, 0.63) |
|  | M | 0.16 (0.10, 0.21) | 0.16 (0.12, 0.20) | 0.17 (0.14, 0.19) | 0.18 (0.16, 0.21) | 0.21 (0.19, 0.23) | 0.25 (0.23, 0.27) | 0.30 (0.28, 0.33) | 0.37 (0.33, 0.41) | 0.46 (0.39, 0.52) | 0.56 (0.46, 0.65) |
|  |  |  |  |  |  |  |  |  |  |  |  |
| Glaucoma* | F | 0.11 (0.02, 0.19) | 0.05 (0.01, 0.08) | 0.09 (0.05, 0.12) | 0.10 (0.08, 0.13) | 0.12 (0.09, 0.14) | 0.15 (0.13, 0.17) | 0.20 (0.18, 0.21) | 0.21 (0.18, 0.23) | 0.26 (0.20, 0.32) |  |
|  | M | 0 | 0.06 (0.02, 0.10) | 0.06 (0.04, 0.09) | 0.09 (0.06, 0.12) | 0.10 (0.08, 0.13) | 0.17 (0.14, 0.19) | 0.21 (0.18, 0.24) | 0.21 (0.16, 0.26) | 0.14 (0.01, 0.27) |  |
|  |  |  |  |  |  |  |  |  |  |  |  |
|  |  | Age | | | | | | | | | |
| Ear |  | 60 | 65 | 70 | 75 | 80 | 85 | 90 | 95 | 100 | 105 |
| Hearing impairment | F | 0.06 (0.03, 0.09) | 0.07 (0.04, 0.09) | 0.08 (0.06, 0.10) | 0.10 (0.09, 0.12) | 0.14 (0.12, 0.15) | 0.18 (0.17, 0.20) | 0.25 (0.24, 0.27) | 0.35 (0.33, 0.37) | 0.47 (0.43, 0.50) | 0.60 (0.54, 0.66) |
|  | M | 0.05 (0.02, 0.08) | 0.07 (0.04, 0.09) | 0.09 (0.07, 0.11) | 0.13 (0.11, 0.15) | 0.17 (0.15, 0.20) | 0.24 (0.22, 0.26) | 0.32 (0.30, 0.35) | 0.42 (0.38, 0.46) | 0.53 (0.47, 0.60) | 0.65 (0.55, 0.75) |
|  |  |  |  |  |  |  |  |  |  |  |  |
|  |  | Age | | | | | | | | | |
| Skin |  | 60 | 65 | 70 | 75 | 80 | 85 | 90 | 95 | 100 | 105 |
| Other skin condition | F | 0.03 (0.01, 0.06) | 0.03 (0.01, 0.04) | 0.03 (0.01, 0.04) | 0.02 (0.02, 0.03) | 0.03 (0.02, 0.03) | 0.03 (0.02, 0.04) | 0.03 (0.02, 0.04) | 0.04 (0.03, 0.05) | 0.05 (0.03, 0.07) | 0.06 (0.03, 0.09) |
|  | M | 0.03 (0.00, 0.05) | 0.03 (0.01, 0.04) | 0.03 (0.02, 0.04) | 0.03 (0.02, 0.04) | 0.03 (0.02, 0.04) | 0.03 (0.02, 0.04) | 0.04 (0.02, 0.05) | 0.04 (0.02, 0.05) | 0.04 (0.02, 0.07) | 0.05 (0.00, 0.09) |
|  |  |  |  |  |  |  |  |  |  |  |  |
| Wound | F | 0.03 (0.01, 0.06) | 0.03 (0.01, 0.05) | 0.03 (0.02, 0.05) | 0.04 (0.03, 0.05) | 0.04 (0.03, 0.05) | 0.05 (0.04, 0.06) | 0.06 (0.05, 0.07) | 0.07 (0.06, 0.08) | 0.09 (0.07, 0.11) | 0.12 (0.08, 0.16) |
|  | M | 0.04 (0.01, 0.08) | 0.04 (0.02, 0.06) | 0.04 (0.03, 0.06) | 0.04 (0.03, 0.06) | 0.05 (0.03, 0.06) | 0.05 (0.04, 0.06) | 0.05 (0.04, 0.06) | 0.05 (0.04, 0.07) | 0.06 (0.03, 0.08) | 0.06 (0.01, 0.11) |
|  |  |  |  |  |  |  |  |  |  |  |  |
| Skin allergies | F | 0.34 (0.27, 0.42) | 0.33 (0.28, 0.38) | 0.32 (0.28, 0.36) | 0.31 (0.29, 0.34) | 0.31 (0.29, 0.33) | 0.31 (0.29, 0.33) | 0.31 (0.29, 0.33) | 0.32 (0.29, 0.34) | 0.33 (0.29, 0.36) | 0.34 (0.28, 0.39) |
|  | M | 0.32 (0.25, 0.40) | 0.33 (0.28, 0.38) | 0.34 (0.30, 0.37) | 0.34 (0.31, 0.37) | 0.34 (0.31, 0.36) | 0.33 (0.31, 0.36) | 0.32 (0.30, 0.35) | 0.31 (0.28, 0.35) | 0.30 (0.24, 0.36) | 0.28 (0.20, 0.37) |
|  |  |  |  |  |  |  |  |  |  |  |  |
| Skin infection | F | 0.05 (0.02, 0.08) | 0.05 (0.02, 0.07) | 0.04 (0.03, 0.06) | 0.04 (0.03, 0.05) | 0.04 (0.03, 0.05) | 0.05 (0.04, 0.05) | 0.05 (0.04, 0.06) | 0.06 (0.05, 0.07) | 0.07 (0.05, 0.09) | 0.08 (0.05, 0.11) |
|  | M | 0.05 (0.02, 0.09) | 0.05 (0.03, 0.08) | 0.05 (0.04, 0.07) | 0.05 (0.04, 0.06) | 0.05 (0.04, 0.06) | 0.05 (0.04, 0.06) | 0.05 (0.04, 0.06) | 0.05 (0.04, 0.07) | 0.06 (0.03, 0.08) | 0.06 (0.01, 0.10) |

Model-based estimates of condition prevalence generated using mixed effects probit regression. Prevalence estimates are expressed as proportions. N=9436 people in residential aged care during 2014-2017 from 68 facilities in NSW and ACT. F = estimates for females, M = estimates for males.

Supplemental Table 5. Estimated number of cases in Australian RACFs in 2016-17 by age and sex

|  |  | Age | | | | | | | | |  |
| --- | --- | --- | --- | --- | --- | --- | --- | --- | --- | --- | --- |
| Circulatory Disease |  | 60 | 65 | 70 | 75 | 80 | 85 | 90 | 95 | 100+ | Total |
| Any Type | F | 1114 | 2539 | 5220 | 10004 | 19031 | 31404 | 30130 | 12034 | 2014 | 113,489 |
|  | M | 1427 | 2936 | 5016 | 7354 | 10417 | 13544 | 9996 | 3003 | 330 | 54,023 |
|  |  |  |  |  |  |  |  |  |  |  | **167,512** |
| Heart disease | F | 112 | 370 | 1028 | 2490 | 5650 | 10572 | 11014 | 4597 | 777 | 36,611 |
|  | M | 311 | 780 | 1575 | 2654 | 4207 | 5975 | 4711 | 1483 | 168 | 21,865 |
|  |  |  |  |  |  |  |  |  |  |  | **58,476** |
| Hypertension | F | 444 | 1253 | 2993 | 6353 | 12889 | 22013 | 21307 | 8375 | 1342 | 76,969 |
|  | M | 616 | 1499 | 2874 | 4538 | 6701 | 8833 | 6435 | 1854 | 189 | 33,539 |
|  |  |  |  |  |  |  |  |  |  |  | **110,508** |
| Heart failure | F | 43 | 127 | 337 | 816 | 1914 | 3786 | 4242 | 1931 | 360 | 13,557 |
|  | M | 69 | 180 | 383 | 690 | 1178 | 1808 | 1544 | 527 | 65 | 6,444 |
|  |  |  |  |  |  |  |  |  |  |  | **20,001** |
| Arrhythmias | F | 80 | 274 | 781 | 1930 | 4441 | 8382 | 8758 | 3645 | 610 | 28,900 |
|  | M | 165 | 474 | 1059 | 1909 | 3157 | 4584 | 3634 | 1132 | 125 | 16,239 |
|  |  |  |  |  |  |  |  |  |  |  | **45,139** |
| Myocardial Infarction (MI) | F | 18 | 55 | 148 | 353 | 794 | 1471 | 1508 | 612 | 99 | 5,058 |
|  | M | 83 | 194 | 371 | 592 | 888 | 1185 | 869 | 251 | 26 | 4,460 |
|  |  |  |  |  |  |  |  |  |  |  | **9,518** |
| Dyslipidaemia | F | 537 | 1354 | 2945 | 5744 | 10694 | 16509 | 13985 | 4554 | 555 | 56,877 |
|  | M | 534 | 1359 | 2637 | 4124 | 5924 | 7443 | 5026 | 1287 | 109 | 28,443 |
|  |  |  |  |  |  |  |  |  |  |  | **85,320** |
| Cerebrovascular disease (including stroke) | F | 259 | 603 | 1283 | 2533 | 4907 | 8108 | 7639 | 2931 | 460 | 28,723 |
|  | M | 544 | 1104 | 1861 | 2672 | 3659 | 4520 | 3100 | 843 | 81 | 18,383 |
|  |  |  |  |  |  |  |  |  |  |  | **47,105** |
|  |  | Age | | | | | | | | |  |
| Neoplasms |  | 60 | 65 | 70 | 75 | 80 | 85 | 90 | 95 | 100+ | Total |
| Any neoplasm | F | 252 | 558 | 1150 | 2248 | 4392 | 7447 | 7323 | 2987 | 509 | 26,866 |
|  | M | 174 | 510 | 1143 | 2048 | 3344 | 4767 | 3691 | 1117 | 119 | 16,913 |
|  |  |  |  |  |  |  |  |  |  |  | **43,780** |
| Lung cancer | F | 57 | 95 | 144 | 204 | 280 | 326 | 213 | 56 | 6 | 1,381 |
|  | M | 53 | 88 | 124 | 152 | 181 | 199 | 123 | 31 | 3 | 954 |
|  |  |  |  |  |  |  |  |  |  |  | **2,335** |
| Colorectal cancer | F | 23 | 55 | 123 | 263 | 563 | 1048 | 1132 | 507 | 95 | 3,808 |
|  | M | 11 | 43 | 124 | 266 | 483 | 719 | 546 | 152 | 14 | 2,357 |
|  |  |  |  |  |  |  |  |  |  |  | **6,165** |
| Skin cancer | F | 28 | 81 | 212 | 512 | 1211 | 2444 | 2815 | 1326 | 258 | 8,886 |
|  | M | 71 | 175 | 362 | 644 | 1107 | 1738 | 1538 | 549 | 71 | 6,256 |
|  |  |  |  |  |  |  |  |  |  |  | **15,142** |
| Metastatic cancer | F | 46 | 83 | 136 | 202 | 288 | 338 | 218 | 54 | 5 | 1,371 |
|  | M | 56 | 112 | 185 | 259 | 344 | 409 | 268 | 69 | 6 | 1,710 |
|  |  |  |  |  |  |  |  |  |  |  | **3,081** |
| Breast cancer* | F | 33 | 168 | 405 | 755 | 1108 | 2195 | 2100 | 761 | 136 | 7,662 |
|  |  |  |  |  |  |  |  |  |  |  |  |
| Prostate cancer* | M | 0 | 143 | 277 | 947 | 1344 | 1663 | 1342 | 279 | 37 | 6,032 |
|  |  |  |  |  |  |  |  |  |  |  |  |
| Leukemia or Lymphoma* | F | 0 | 0 | 44 | 478 | 657 | 877 | 1004 | 237 | 36 | 3,333 |
|  | M | 48 | 48 | 359 | 290 | 538 | 486 | 401 | 134 | 25 | 2,376 |
|  |  |  |  |  |  |  |  |  |  |  | **5,709** |
|  |  | Age | | | | | | | | |  |
| Respiratory |  | 60 | 65 | 70 | 75 | 80 | 85 | 90 | 95 | 100+ |  |
| Chronic lower respiratory disease | F | 652 | 1279 | 2367 | 4198 | 7516 | 11796 | 10838 | 4166 | 675 | 43,487 |
|  | M | 634 | 1191 | 1929 | 2753 | 3874 | 5083 | 3834 | 1190 | 136 | 20,626 |
|  |  |  |  |  |  |  |  |  |  |  | **64,113** |
|  |  | Age | | | | | | | | |  |
| Endocrine |  | 60 | 65 | 70 | 75 | 80 | 85 | 90 | 95 | 100+ |  |
| Any endocrine disorder | F | 619 | 1348 | 2686 | 4992 | 9124 | 14220 | 12588 | 4500 | 649 | 50,727 |
|  | M | 533 | 1207 | 2173 | 3221 | 4425 | 5324 | 3434 | 838 | 68 | 21,222 |
|  |  |  |  |  |  |  |  |  |  |  | **71,949** |
| Diabetes | F | 468 | 1006 | 1957 | 3514 | 6118 | 8929 | 7246 | 2312 | 288 | 31,837 |
|  | M | 447 | 1042 | 1897 | 2798 | 3762 | 4347 | 2625 | 579 | 40 | 17,537 |
|  |  |  |  |  |  |  |  |  |  |  | **49,374** |
| Disorders of the thyroid gland | F | 228 | 528 | 1114 | 2191 | 4231 | 6979 | 6569 | 2521 | 397 | 24,758 |
|  | M | 119 | 243 | 424 | 639 | 935 | 1257 | 957 | 296 | 33 | 4,903 |
|  |  |  |  |  |  |  |  |  |  |  | **29,661** |
|  |  | Age | | | | | | | | |  |
| Nervous system |  | 60 | 65 | 70 | 75 | 80 | 85 | 90 | 95 | 100+ |  |
| Any disease of the nervous system | F | 654 | 1166 | 1925 | 2983 | 4556 | 5937 | 4398 | 1319 | 161 | 23,098 |
|  | M | 993 | 1677 | 2374 | 2875 | 3325 | 3464 | 1999 | 456 | 37 | 17,200 |
|  |  |  |  |  |  |  |  |  |  |  | **40,298** |
| Parkinson's disease | F | 72 | 200 | 463 | 926 | 1688 | 2436 | 1842 | 513 | 52 | 8,191 |
|  | M | 75 | 279 | 682 | 1194 | 1727 | 1968 | 1070 | 189 | 9 | 7,193 |
|  |  |  |  |  |  |  |  |  |  |  | **15,383** |
| Multiple sclerosis | F | 45 | 72 | 98 | 111 | 108 | 76 | 25 | 3 | 0 | 537 |
|  | M | 84 | 81 | 58 | 30 | 12 | 4 | 0 | 0 | 0 | 270 |
|  |  |  |  |  |  |  |  |  |  |  | **806** |
| Epilepsy | F | 295 | 451 | 636 | 845 | 1116 | 1274 | 841 | 230 | 26 | 5,714 |
|  | M | 351 | 557 | 723 | 779 | 775 | 666 | 302 | 51 | 3 | 4,207 |
|  |  |  |  |  |  |  |  |  |  |  | **9,921** |
| Brain injury | F | 45 | 63 | 80 | 93 | 105 | 99 | 52 | 11 | 1 | 548 |
|  | M | 230 | 249 | 215 | 152 | 99 | 57 | 18 | 2 | 0 | 1,021 |
|  |  |  |  |  |  |  |  |  |  |  | **1,569** |
| Motor Neurone Disease* | F | 0 | 0 | 131 | 48 | 47 | 44 | 104 | 26 | 0 | 400 |
|  | M | 0 | 95 | 108 | 0 | 0 | 112 | 0 | 0 | 0 | 315 |
|  |  |  |  |  |  |  |  |  |  |  | **715** |
|  |  | Age | | | | | | | | |  |
|  |  | 45 | 50 | 55 | 60 | 65 | 70 | 75 | 80 | 85 |  |
| Huntington's disease | F | 3 | 4 | 9 | 16 | 25 | 32 | 32 | 27 | 15 | 164 |
|  | M | 30 | 25 | 34 | 41 | 43 | 38 | 28 | 19 | 12 | 271 |
|  |  |  |  |  |  |  |  |  |  |  | **435** |
|  |  | Age | | | | | | | | |  |
| Digestive |  | 60 | 65 | 70 | 75 | 80 | 85 | 90 | 95 | 100+ |  |
| Other digestive (including inflammatory) condition | F | 207 | 509 | 1126 | 2298 | 4568 | 7707 | 7382 | 2869 | 455 | 27,121 |
|  | M | 253 | 523 | 928 | 1445 | 2208 | 3128 | 2533 | 839 | 102 | 11,960 |
|  |  |  |  |  |  |  |  |  |  |  | **39,082** |
| Liver disease | F | 139 | 200 | 272 | 356 | 474 | 560 | 394 | 118 | 15 | 2,529 |
|  | M | 253 | 334 | 373 | 364 | 349 | 313 | 163 | 36 | 3 | 2,187 |
|  |  |  |  |  |  |  |  |  |  |  | **4,716** |
| PUD, GORD and other acid related conditions | F | 746 | 1637 | 3321 | 6354 | 12099 | 19932 | 18988 | 7476 | 1224 | 71,776 |
|  | M | 824 | 1685 | 2905 | 4326 | 6240 | 8255 | 6182 | 1878 | 208 | 32,503 |
|  |  |  |  |  |  |  |  |  |  |  | **104,279** |
| Diarrhoea | F | 9 | 30 | 82 | 190 | 401 | 670 | 593 | 198 | 25 | 2,199 |
|  | M | 26 | 45 | 69 | 98 | 143 | 205 | 176 | 65 | 9 | 836 |
|  |  |  |  |  |  |  |  |  |  |  | **3,035** |
| Constipation | F | 1187 | 2333 | 4375 | 7973 | 14876 | 24661 | 24220 | 10045 | 1764 | 91,434 |
|  | M | 1251 | 2385 | 3903 | 5608 | 7914 | 10371 | 7778 | 2389 | 269 | 41,868 |
|  |  |  |  |  |  |  |  |  |  |  | **133,302** |
| Nausea or vomiting | F | 335 | 664 | 1254 | 2292 | 4269 | 7037 | 6854 | 2819 | 493 | 26,017 |
|  | M | 371 | 568 | 783 | 1005 | 1348 | 1796 | 1459 | 515 | 70 | 7,915 |
|  |  |  |  |  |  |  |  |  |  |  | **33,932** |
| Faecal incontinence | F | 380 | 755 | 1412 | 2531 | 4572 | 7231 | 6685 | 2582 | 419 | 26,567 |
|  | M | 414 | 813 | 1338 | 1896 | 2584 | 3206 | 2230 | 622 | 62 | 13,166 |
|  |  |  |  |  |  |  |  |  |  |  | **39,733** |
| Double incontinence | F | 302 | 591 | 1093 | 1941 | 3486 | 5499 | 5089 | 1975 | 323 | 20,301 |
|  | M | 283 | 572 | 958 | 1367 | 1857 | 2271 | 1539 | 413 | 39 | 9,300 |
|  |  |  |  |  |  |  |  |  |  |  | **29,601** |
|  |  | Age | | | | | | | | |  |
| Diseases of the urinary system |  | 60 | 65 | 70 | 75 | 80 | 85 | 90 | 95 | 100+ |  |
| Urinary incontinence | F | 796 | 1618 | 3109 | 5735 | 10695 | 17509 | 16805 | 6758 | 1146 | 64,170 |
|  | M | 734 | 1451 | 2427 | 3514 | 4933 | 6353 | 4630 | 1367 | 147 | 25,554 |
|  |  |  |  |  |  |  |  |  |  |  | **89,725** |
| Renal disease | F | 163 | 364 | 748 | 1442 | 2753 | 4515 | 4251 | 1642 | 262 | 16,140 |
|  | M | 114 | 325 | 714 | 1265 | 2045 | 2885 | 2202 | 653 | 67 | 10,271 |
|  |  |  |  |  |  |  |  |  |  |  | **26,411** |
| UTI | F | 140 | 344 | 775 | 1630 | 3381 | 6020 | 6154 | 2585 | 450 | 21,478 |
|  | M | 79 | 191 | 371 | 597 | 891 | 1173 | 840 | 234 | 23 | 4,400 |
|  |  |  |  |  |  |  |  |  |  |  | **25,878** |
|  |  | Age | | | | | | | | |  |
| Mental & behavioural disorders |  | 60 | 65 | 70 | 75 | 80 | 85 | 90 | 95 | 100+ |  |
| Any mental or behavioural disorder | F | 1449 | 2969 | 5712 | 10506 | 19456 | 31484 | 29712 | 11668 | 1915 | 114,872 |
|  | M | 1681 | 3242 | 5294 | 7512 | 10369 | 13159 | 9450 | 2739 | 286 | 53,733 |
|  |  |  |  |  |  |  |  |  |  |  | **168,605** |
| Other mental or behavioural disorder | F | 209 | 346 | 512 | 688 | 876 | 907 | 505 | 107 | 8 | 4,159 |
|  | M | 591 | 981 | 1307 | 1412 | 1358 | 1074 | 419 | 56 | 2 | 7,200 |
|  |  |  |  |  |  |  |  |  |  |  | **11,359** |
| Dementia | F | 576 | 1398 | 3049 | 6136 | 12094 | 20384 | 19686 | 7796 | 1273 | 72,392 |
|  | M | 470 | 1308 | 2703 | 4428 | 6623 | 8693 | 6204 | 1715 | 162 | 32,307 |
|  |  |  |  |  |  |  |  |  |  |  | **104,698** |
| Cognitive impairment, memory loss | F | 284 | 639 | 1335 | 2632 | 5167 | 8777 | 8621 | 3502 | 592 | 31,549 |
|  | M | 525 | 970 | 1549 | 2189 | 3063 | 4013 | 3034 | 948 | 110 | 16,401 |
|  |  |  |  |  |  |  |  |  |  |  | **47,949** |
| Schizophrenia, paranoid or psychotic states | F | 473 | 858 | 1423 | 2182 | 3237 | 4004 | 2734 | 730 | 76 | 15,715 |
|  | M | 309 | 576 | 868 | 1077 | 1223 | 1189 | 601 | 111 | 7 | 5,962 |
|  |  |  |  |  |  |  |  |  |  |  | **21,677** |
| Delirium | F | 10 | 46 | 157 | 586 | 1087 | 2103 | 2151 | 834 | 123 | 7,098 |
|  | M | 14 | 60 | 177 | 378 | 679 | 989 | 726 | 193 | 16 | 3,232 |
|  |  |  |  |  |  |  |  |  |  |  | **10,331** |
| Depression | F | 1007 | 2038 | 3854 | 6918 | 12405 | 19258 | 17251 | 6355 | 966 | 70,053 |
|  | M | 1068 | 2030 | 3256 | 4510 | 6026 | 7338 | 5008 | 1368 | 134 | 30,738 |
|  |  |  |  |  |  |  |  |  |  |  | **100,791** |
| Anxiety & stress related disorders | F | 528 | 1076 | 2040 | 3661 | 6536 | 10074 | 8941 | 3260 | 490 | 36,606 |
|  | M | 523 | 893 | 1310 | 1692 | 2156 | 2567 | 1762 | 500 | 53 | 11,454 |
|  |  |  |  |  |  |  |  |  |  |  | **48,060** |
|  |  | Age | | | | | | | | |  |
| Musculoskeletal and soft tissue |  | 60 | 65 | 70 | 75 | 80 | 85 | 90 | 95 | 100+ |  |
| Other musculoskeletal or soft tissue disorder | F | 49 | 125 | 289 | 612 | 1262 | 2200 | 2170 | 867 | 141 | 7,715 |
|  | M | 92 | 191 | 330 | 491 | 700 | 906 | 656 | 190 | 20 | 3,576 |
|  |  |  |  |  |  |  |  |  |  |  | **11,292** |
| Osteoporosis | F | 233 | 657 | 1624 | 3624 | 7783 | 14093 | 14465 | 6044 | 1038 | 49,561 |
|  | M | 152 | 350 | 671 | 1105 | 1748 | 2518 | 2040 | 666 | 79 | 9,330 |
|  |  |  |  |  |  |  |  |  |  |  | **58,890** |
| Arthritis | F | 422 | 1148 | 2728 | 5859 | 12148 | 21344 | 21390 | 8784 | 1493 | 75,315 |
|  | M | 457 | 1128 | 2251 | 3757 | 5915 | 8368 | 6600 | 2088 | 239 | 30,802 |
|  |  |  |  |  |  |  |  |  |  |  | **106,116** |
| Rheumatoid arthritis | F | 30 | 87 | 216 | 459 | 891 | 1379 | 1130 | 348 | 40 | 4,579 |
|  | M | 7 | 28 | 76 | 153 | 260 | 357 | 245 | 60 | 5 | 1,192 |
|  |  |  |  |  |  |  |  |  |  |  | **5,771** |
| Gout | F | 30 | 105 | 301 | 735 | 1639 | 2928 | 2815 | 1042 | 149 | 9,744 |
|  | M | 127 | 331 | 690 | 1191 | 1915 | 2729 | 2136 | 659 | 72 | 9,851 |
|  |  |  |  |  |  |  |  |  |  |  | **19,594** |
|  |  | Age | | | | | | | | |  |
| Pain |  | 60 | 65 | 70 | 75 | 80 | 85 | 90 | 95 | 100+ |  |
| Pain & pain syndromes | F | 460 | 1064 | 2240 | 4387 | 8443 | 13894 | 13068 | 5017 | 790 | 49,362 |
|  | M | 583 | 1145 | 1910 | 2769 | 3902 | 5059 | 3722 | 1112 | 121 | 20,324 |
|  |  |  |  |  |  |  |  |  |  |  | **69,687** |
|  |  | Age | | | | | | | | |  |
| Visual |  | 60 | 65 | 70 | 75 | 80 | 85 | 90 | 95 | 100+ |  |
| Visual impairment | F | 245 | 392 | 653 | 1162 | 2337 | 4574 | 5719 | 3177 | 763 | 19,022 |
|  | M | 220 | 375 | 585 | 858 | 1321 | 2005 | 1832 | 712 | 104 | 8,012 |
|  |  |  |  |  |  |  |  |  |  |  | **27,035** |
| Cataracts | F | 51 | 173 | 491 | 1212 | 2784 | 5228 | 5407 | 2212 | 361 | 17,921 |
|  | M | 30 | 113 | 305 | 628 | 1130 | 1710 | 1357 | 407 | 41 | 5,721 |
|  |  |  |  |  |  |  |  |  |  |  | **23,642** |
| Retinopathy | F | 38 | 90 | 213 | 508 | 1274 | 2893 | 3926 | 2246 | 538 | 11,725 |
|  | M | 31 | 62 | 117 | 214 | 424 | 834 | 986 | 486 | 87 | 3,241 |
|  |  |  |  |  |  |  |  |  |  |  | **14,966** |
| Dry eyes and other eye issues | F | 307 | 649 | 1332 | 2689 | 5613 | 10461 | 11571 | 5395 | 1061 | 39,078 |
|  | M | 291 | 556 | 951 | 1485 | 2359 | 3579 | 3171 | 1163 | 157 | 13,711 |
|  |  |  |  |  |  |  |  |  |  |  | **52,789** |
| Glaucoma* | F | 331 | 291 | 1004 | 2247 | 4742 | 9994 | 12494 | 5093 | 1100 | 37,296 |
|  | M | 0 | 428 | 718 | 1450 | 2308 | 4748 | 4312 | 1262 | 101 | 15,327 |
|  |  |  |  |  |  |  |  |  |  |  | **52,622** |
|  |  | Age | | | | | | | | |  |
| Ear |  | 60 | 65 | 70 | 75 | 80 | 85 | 90 | 95 | 100+ |  |
| Hearing impairment | F | 91 | 210 | 485 | 1133 | 2783 | 6166 | 8096 | 4427 | 997 | 24,390 |
|  | M | 88 | 230 | 515 | 1015 | 1950 | 3428 | 3373 | 1320 | 183 | 12,102 |
|  |  |  |  |  |  |  |  |  |  |  | **36,491** |
|  |  | Age | | | | | | | | |  |
| Skin |  | 60 | 65 | 70 | 75 | 80 | 85 | 90 | 95 | 100+ |  |
| Other skin condition | F | 50 | 88 | 154 | 273 | 520 | 920 | 1008 | 487 | 104 | 3,602 |
|  | M | 50 | 96 | 161 | 237 | 345 | 471 | 370 | 120 | 14 | 1,864 |
|  |  |  |  |  |  |  |  |  |  |  | **5,466** |
| Wound | F | 51 | 103 | 205 | 408 | 850 | 1612 | 1851 | 916 | 196 | 6,191 |
|  | M | 82 | 154 | 249 | 357 | 507 | 673 | 516 | 163 | 19 | 2,720 |
|  |  |  |  |  |  |  |  |  |  |  | **8,911** |
| Skin allergies | F | 536 | 1043 | 1934 | 3472 | 6360 | 10322 | 9913 | 4026 | 696 | 38,303 |
|  | M | 602 | 1168 | 1915 | 2722 | 3751 | 4740 | 3386 | 979 | 103 | 19,367 |
|  |  |  |  |  |  |  |  |  |  |  | **57,670** |
| Skin infection | F | 78 | 144 | 260 | 470 | 892 | 1542 | 1620 | 738 | 146 | 5,891 |
|  | M | 100 | 186 | 296 | 415 | 575 | 742 | 552 | 169 | 19 | 3,053 |
|  |  |  |  |  |  |  |  |  |  |  | **8,943** |

Model-based estimates of condition cases generated using mixed effects probit regression, except for breast cancer and prostate cancer which were tabulated in a frequency table (could not generate estimates from the regression models). Condition prevalence rates were applied to the Australian RACF age distribution in 2016-17 reported by the Australian Institute of Health and Welfare. *Indicates that model-based estimates could not be calculated and simple descriptive statistics by age group were instead reported.

Supplemental Table 6. Agreement between ACFI data and EHR data (n=9436)

| **Condition Category** |  |  | **EHR: Resident notes & medications** | |  | Overall prevalence |
| --- | --- | --- | --- | --- | --- | --- |
|  | **ACFI** |  | Without | With | Kappa (95%CI) |  |
| *Circulatory diseases* | Any circulatory condition | Without | 644 | 3780 | 0.14 (0.12, 0.15) | 93.2% |
|  |  | With | 82 | 4930 |  |  |
|  |  |  |  |  |  |  |
|  |  |  | Without | With | Kappa (95%CI) |  |
|  | Heart disease | Without | 6193 | 1770 | 0.36 (0.34, 0.38) | 34.4% |
|  |  | With | 423 | 1050 |  |  |
|  |  |  |  |  |  |  |
|  |  |  | Without | With | Kappa (95%CI) |  |
|  | Hypertension | Without | 3631 | 3185 | 0.31 (0.29, 0.32) | 61.5% |
|  |  | With | 315 | 2305 |  |  |
|  |  |  |  |  |  |  |
|  |  |  | Without | With | Kappa (95%CI) |  |
|  | Heart failure | Without | 8271 | 642 | 0.47 (0.44, 0.50) | 12.3% |
|  |  | With | 128 | 395 |  |  |
|  |  |  |  |  |  |  |
|  |  |  | Without | With | Kappa (95%CI) |  |
|  | MI | Without | 8908 | 372 | 0.26 (0.21, 0.30) | 5.6% |
|  |  | With | 72 | 84 |  |  |
|  |  |  |  |  |  |  |
|  |  |  | Without | With | Kappa (95%CI) |  |
|  | Dyslipidaemia | Without | 5271 | 3665 | 0.11 (0.10, 0.12) | 44.1% |
|  |  | With | 53 | 447 |  |  |
|  |  |  |  |  |  |  |
|  |  |  | Without | With | Kappa (95%CI) |  |
|  | Cerebrovascular disease (including stroke) | Without | 6927 | 1060 | 0.58 (0.56, 0.60) | 26.6% |
|  |  | With | 226 | 1223 |  |  |
|  |  |  |  |  |  |  |
|  |  |  |  |  |  |  |
| *Neoplasms* |  |  | Without | With | Kappa (95%CI) |  |
|  | Any neoplasm | Without | 7047 | 1443 | 0.40 (0.38, 0.43) | 25.3% |
|  |  | With | 177 | 769 |  |  |
|  |  |  |  |  |  |  |
|  |  |  | Without | With | Kappa (95%CI) |  |
|  | Breast cancer | Without | 8964 | 313 | 0.42 (0.37, 0.47) | 5.0% |
|  |  | With | 28 | 131 |  |  |
|  |  |  |  |  |  |  |
|  |  |  | Without | With | Kappa (95%CI) |  |
|  | Prostate cancer | Without | 9109 | 167 | 0.55 (0.49, 0.60) | 3.5% |
|  |  | With | 34 | 126 |  |  |
|  |  |  |  |  |  |  |
|  |  |  | Without | With | Kappa (95%CI) |  |
|  | Lung cancer | Without | 9333 | 35 | 0.59 (0.49, 0.68) | 1.1% |
|  |  | With | 25 | 43 |  |  |
|  |  |  |  |  |  |  |
|  |  |  | Without | With | Kappa (95%CI) |  |
|  | Colorectal cancer | Without | 9087 | 161 | 0.50 (0.44, 0.55) | 3.7% |
|  |  | With | 69 | 119 |  |  |
|  |  |  |  |  |  |  |
|  |  |  | Without | With | Kappa (95%CI) |  |
|  | Skin cancer | Without | 8532 | 709 | 0.26 (0.22, 0.29) | 9.6% |
|  |  | With | 47 | 148 |  |  |
|  |  |  |  |  |  |  |
|  |  |  | Without | With | Kappa (95%CI) |  |
|  | Blood & lymph cancers | Without | 9287 | 107 | 0.37 (0.28, 0.46) | 1.6% |
|  |  | With | 8 | 34 |  |  |
|  |  |  |  |  |  |  |
|  |  |  | Without | With | Kappa (95%CI) |  |
|  | Chronic lower respiratory disease | Without | 6145 | 2196 | 0.37 (0.35, 0.39) | 34.9% |
|  |  | With | 64 | 1031 |  |  |
|  |  |  |  |  |  |  |
|  |  |  |  |  |  |  |
| *Endocrine* |  |  | Without | With | Kappa (95%CI) |  |
|  | Any endocrine disorder | Without | 5878 | 1870 | 0.50 (0.49, 0.52) | 37.7% |
|  |  | With | 75 | 1613 |  |  |
|  |  |  |  |  |  |  |
|  |  |  | Without | With | Kappa (95%CI) |  |
|  | Diabetes mellitus | Without | 7100 | 970 | 0.67 (0.65, 0.69) | 24.8% |
|  |  | With | 23 | 1343 |  |  |
|  |  |  |  |  |  |  |
|  |  |  | Without | With | Kappa (95%CI) |  |
|  | Disorders of the thyroid | Without | 7899 | 1209 | 0.28 (0.25, 0.30) | 16.3% |
|  |  | With | 37 | 291 |  |  |
|  |  |  |  |  |  |  |
|  |  |  |  |  |  |  |
| *Diseases of the nervous system* |  |  | Without | With | Kappa (95%CI) |  |
|  | Any disease of the nervous system | Without | 7506 | 953 | 0.48 (0.45, 0.50) | 20.5% |
|  |  | With | 253 | 724 |  |  |
|  |  |  |  |  |  |  |
|  |  |  | Without | With | Kappa (95%CI) |  |
|  | Parkinson's disease | Without | 8679 | 332 | 0.68 (0.65, 0.72) | 8.0% |
|  |  | With | 15 | 410 |  |  |
|  |  |  |  |  |  |  |
|  |  |  | Without | With | Kappa (95%CI) |  |
|  | Motor neurone disease | Without | 9418 | 10 | 0.43 (0.18, 0.69) | 0.2% |
|  |  | With | 3 | 5 |  |  |
|  |  |  |  |  |  |  |
|  |  |  | Without | With | Kappa (95%CI) |  |
|  | Multiple sclerosis | Without | 9408 | 3 | 0.81 (0.69, 0.93) | 0.3% |
|  |  | With | 6 | 19 |  |  |
|  |  |  |  |  |  |  |
|  |  |  | Without | With | Kappa (95%CI) |  |
|  | Epilepsy | Without | 9020 | 251 | 0.52 (0.47, 0.57) | 4.4% |
|  |  | With | 14 | 151 |  |  |
|  |  |  |  |  |  |  |
|  |  |  | Without | With | Kappa (95%CI) |  |
|  | Huntington's disease | Without | 9424 | 3 | 0.86 (0.70, 1.00) | 0.1% |
|  |  | With | 0 | 9 |  |  |
|  |  |  |  |  |  |  |
|  |  |  |  |  |  |  |
| *Digestive* |  |  | Without | With | Kappa (95%CI) |  |
|  | Other digestive condition (incl. inflammatory conditions) | Without | 7314 | 1003 | 0.20 (0.17, 0.23) | 22.5% |
|  |  | With | 737 | 382 |  |  |
|  |  |  |  |  |  |  |
|  |  |  | Without | With | Kappa (95%CI) |  |
|  | Liver disease | Without | 9237 | 146 | 0.30 (0.22, 0.38) | 2.1% |
|  |  | With | 17 | 36 |  |  |
|  |  |  |  |  |  |  |
|  |  |  | Without | With | Kappa (95%CI) |  |
|  | Diarrhoea | Without | 9286 | 137 | 0.02 (-0.01, 0.06) | 1.6% |
|  |  | With | 11 | 2 |  |  |
|  |  |  |  |  |  |  |
|  |  |  | Without | With | Kappa (95%CI) |  |
|  | Nausea or vomiting | Without | 7531 | 1885 | 0.01 (0.01, 0.02) | 20.2% |
|  |  | With | 5 | 15 |  |  |
|  |  |  |  |  |  |  |
|  |  |  | Without | With | Kappa (95%CI) |  |
|  | Faecal incontinence | Without | 7207 | 810 | 0.32 (0.30, 0.35) | 23.6% |
|  |  | With | 816 | 603 |  |  |
|  |  |  |  |  |  |  |
|  |  |  |  |  |  |  |
| *Diseases of the urinary system* |  |  | Without | With | Kappa (95%CI) |  |
|  | Renal disease | Without | 8002 | 830 | 0.35 (0.32, 0.38) | 15.2% |
|  |  | With | 243 | 361 |  |  |
|  |  |  |  |  |  |  |
|  |  |  | Without | With | Kappa (95%CI) |  |
|  | Urinary incontinence | Without | 4602 | 1173 | 0.33 (0.31, 0.35) | 51.2% |
|  |  | With | 1760 | 1901 |  |  |
|  |  |  |  |  |  |  |
|  |  |  | Without | With | Kappa (95%CI) |  |
|  | UTI | Without | 8025 | 1016 | 0.27 (0.24, 0.29) | 15.0% |
|  |  | With | 134 | 261 |  |  |
|  |  |  |  |  |  |  |
|  |  |  |  |  |  |  |
| *Mental and behavioural* |  |  | Without | With | Kappa (95%CI) |  |
|  | Any mental or behavioural disorder | Without | 678 | 637 | 0.51 (0.48, 0.54) | 92.8% |
|  |  | With | 385 | 7736 |  |  |
|  |  |  |  |  |  |  |
|  |  |  | Without | With | Kappa (95%CI) |  |
|  | Other mental or behavioural disorder | Without | 8956 | 235 | 0.42 (0.37, 0.47) | 5.1% |
|  |  | With | 111 | 134 |  |  |
|  |  |  |  |  |  |  |
|  |  |  | Without | With | Kappa (95%CI) |  |
|  | Dementia | Without | 3980 | 249 | 0.77 (0.76, 0.78) | 57.8% |
|  |  | With | 840 | 4367 |  |  |
|  |  |  |  |  |  |  |
|  |  |  | Without | With | Kappa (95%CI) |  |
|  | Cognitive impairment, memory loss | Without | 6933 | 1920 | 0.20 (0.18, 0.22) | 26.5% |
|  |  | With | 184 | 399 |  |  |
|  |  |  |  |  |  |  |
|  |  |  | Without | With | Kappa (95%CI) |  |
|  | Schizophrenia, paranoid or psychotic states | Without | 8563 | 302 | 0.63 (0.60, 0.66) | 9.3% |
|  |  | With | 147 | 424 |  |  |
|  |  |  |  |  |  |  |
|  |  |  | Without | With | Kappa (95%CI) |  |
|  | Delirium | Without | 8856 | 260 | 0.41 (0.37, 0.46) | 6.1% |
|  |  | With | 159 | 161 |  |  |
|  |  |  |  |  |  |  |
|  |  |  | Without | With | Kappa (95%CI) |  |
|  | Depression | Without | 4352 | 598 | 0.60 (0.59, 0.62) | 53.9% |
|  |  | With | 1262 | 3224 |  |  |
|  |  |  |  |  |  |  |
|  |  |  | Without | With | Kappa (95%CI) |  |
|  | Anxiety or stress-related disorders | Without | 7023 | 566 | 0.58 (0.56, 0.60) | 25.6% |
|  |  | With | 656 | 1191 |  |  |
|  |  |  |  |  |  |  |
|  |  |  |  |  |  |  |
| *Musculoskeletal and soft tissue* |  |  | Without | With | Kappa (95%CI) |  |
|  | Other musculoskeletal or soft tissue disorder | Without | 8816 | 322 | 0.15 (0.11, 0.19) | 6.6% |
|  |  | With | 238 | 60 |  |  |
|  |  |  |  |  |  |  |
|  |  |  | Without | With | Kappa (95%CI) |  |
|  | Osteoporosis | Without | 6252 | 1825 | 0.42 (0.40, 0.44) | 33.7% |
|  |  | With | 197 | 1162 |  |  |
|  |  |  |  |  |  |  |
|  |  |  | Without | With | Kappa (95%CI) |  |
|  | Arthritis | Without | 3711 | 1498 | 0.53 (0.52, 0.55) | 60.7% |
|  |  | With | 723 | 3504 |  |  |
|  |  |  |  |  |  |  |
|  |  |  | Without | With | Kappa (95%CI) |  |
|  | Rheumatoid arthritis | Without | 9149 | 70 | 0.64 (0.58, 0.69) | 3.0% |
|  |  | With | 80 | 137 |  |  |
|  |  |  |  |  |  |  |
|  |  |  | Without | With | Kappa (95%CI) |  |
|  | Fracture | Without | 6870 | 1719 | 0.30 (0.28, 0.32) | 27.2% |
|  |  | With | 223 | 624 |  |  |
|  |  |  |  |  |  |  |
|  |  |  | Without | With | Kappa (95%CI) |  |
|  | Amputation | Without | 9285 | 78 | 0.52 (0.44, 0.61) | 1.6% |
|  |  | With | 19 | 54 |  |  |
|  |  |  |  |  |  |  |
|  |  |  | Without | With | Kappa (95%CI) |  |
|  | Back & spine conditions | Without | 8554 | 557 | 0.31 (0.27, 0.34) | 9.3% |
|  |  | With | 145 | 180 |  |  |
|  |  |  |  |  |  |  |
|  |  |  |  |  |  |  |
| *Pain & pain syndromes* |  |  | Without | With | Kappa (95%CI) |  |
|  | Pain & pain syndromes | Without | 5793 | 1639 | 0.37 (0.35, 0.39) | 38.6% |
|  |  | With | 698 | 1306 |  |  |
|  |  |  |  |  |  |  |
|  |  |  |  |  |  |  |
| *Diseases of the visual system* |  |  | Without | With | Kappa (95%CI) |  |
|  | Visual impairment | Without | 7792 | 606 | 0.49 (0.46, 0.52) | 17.4% |
|  |  | With | 411 | 627 |  |  |
|  |  |  |  |  |  |  |
|  |  |  | Without | With | Kappa (95%CI) |  |
|  | Cataracts | Without | 8163 | 894 | 0.30 (0.27, 0.33) | 13.5% |
|  |  | With | 118 | 261 |  |  |
|  |  |  |  |  |  |  |
|  |  |  | Without | With | Kappa (95%CI) |  |
|  | Glaucoma | Without | 7980 | 954 | 0.45 (0.42, 0.48) | 15.4% |
|  |  | With | 27 | 475 |  |  |
|  |  |  |  |  |  |  |
|  |  |  | Without | With | Kappa (95%CI) |  |
|  | Dry eyes and other eye issues | Without | 6414 | 2771 | 0.03 (0.02, 0.05) | 32.0% |
|  |  | With | 122 | 129 |  |  |
|  |  |  |  |  |  |  |
|  |  |  |  |  |  |  |
| *Hearing* |  |  | Without | With | Kappa (95%CI) |  |
|  | Hearing impairment | Without | 7144 | 1519 | 0.37 (0.35, 0.40) | 24.3% |
|  |  | With | 107 | 666 |  |  |
|  |  |  |  |  |  |  |
| *Skin* |  |  | Without | With | Kappa (95%CI) |  |
|  | Other skin condition | Without | 9105 | 50 | 0.04 (0.01, 0.07) | 3.5% |
|  |  | With | 273 | 8 |  |  |
|  |  |  |  |  |  |  |
|  |  |  | Without | With | Kappa (95%CI) |  |
|  | Skin allergies | Without | 6457 | 2850 | 0.06 (0.05, 0.07) | 31.6% |
|  |  | With | 0 | 129 |  |  |
|  |  |  |  |  |  |  |
|  |  |  | Without | With | Kappa (95%CI) |  |
|  | Skin infection | Without | 8910 | 302 | 0.27 (0.22, 0.32) | 5.6% |
|  |  | With | 134 | 90 |  |  |

Overall prevalence is calculated as the sum of those ‘with’ a condition according to either or both data sources divided by the sample denominator of 9436.

Supplemental Table 7. Cluster Analysis – Comorbidity Cluster Characteristics

| **Estimated proportion (95% CI)** | **Cluster 1** | **Cluster 2** | **Cluster 3** | **Cluster 4** | **Cluster 5** | **Cluster 6** | **Cluster 7** |
| --- | --- | --- | --- | --- | --- | --- | --- |
| Probability of being in cluster | 0.15 (0.13, 0.16) | 0.17 (0.15, 0.20) | 0.09 (0.07, 0.11) | 0.19 (0.16, 0.22) | 0.21 (0.18, 0.24) | 0.11 (0.08, 0.14) | 0.09 (0.08, 0.10) |
| Males | 1.00 (0.00, 1.00) | 0.12 (0.10, 0.16) | 0.60 (0.54, 0.67) | 0.11 (0.08, 0.15) | 0.00 (0.00, 1.00) | 0.40 (0.34, 0.47) | 0.50 (0.46, 0.54) |
| Constipation | 0.74 (0.71, 0.76) | 0.89 (0.86, 0.91) | 0.75 (0.70, 0.80) | 0.77 (0.74, 0.80) | 0.72 (0.69, 0.74) | 0.69 (0.64, 0.74) | 0.73 (0.69, 0.76) |
| Hypertension | 0.59 (0.56, 0.63) | 0.72 (0.68, 0.75) | 0.80 (0.73, 0.85) | 0.52 (0.47, 0.56) | 0.72 (0.69, 0.75) | 0.55 (0.49, 0.62) | 0.31 (0.26, 0.36) |
| Arthritis | 0.62 (0.59, 0.65) | 0.81 (0.77, 0.84) | 0.54 (0.49, 0.59) | 0.65 (0.62, 0.69) | 0.65 (0.62, 0.68) | 0.36 (0.30, 0.43) | 0.36 (0.31, 0.40) |
| Dementia | 0.53 (0.49, 0.57) | 0.42 (0.38, 0.47) | 0.43 (0.34, 0.51) | 0.77 (0.72, 0.81) | 0.53 (0.49, 0.57) | 0.97 (0.87, 0.99) | 0.36 (0.32, 0.41) |
| PUD or GORD | 0.62 (0.59, 0.65) | 0.87 (0.83, 0.91) | 0.72 (0.66, 0.78) | 0.45 (0.41, 0.50) | 0.60 (0.55, 0.64) | 0.19 (0.13, 0.26) | 0.49 (0.44, 0.54) |
| Depression | 0.41 (0.37, 0.44) | 0.85 (0.79, 0.89) | 0.69 (0.63, 0.75) | 0.54 (0.49, 0.59) | 0.35 (0.31, 0.39) | 0.40 (0.33, 0.46) | 0.61 (0.57, 0.65) |
| Urinary incontinence | 0.39 (0.36, 0.42) | 0.63 (0.59, 0.66) | 0.40 (0.34, 0.46) | 0.59 (0.56, 0.63) | 0.47 (0.43, 0.50) | 0.59 (0.54, 0.65) | 0.44 (0.40, 0.48) |
| Dyslipidaemia | 0.47 (0.42, 0.51) | 0.52 (0.49, 0.56) | 0.84 (0.77, 0.89) | 0.21 (0.17, 0.26) | 0.47 (0.43, 0.51) | 0.39 (0.32, 0.45) | 0.32 (0.26, 0.37) |
| Pain | 0.34 (0.31, 0.37) | 0.64 (0.60, 0.68) | 0.34 (0.29, 0.39) | 0.45 (0.41, 0.50) | 0.31 (0.27, 0.35) | 0.14 (0.10, 0.21) | 0.34 (0.30, 0.38) |
| Lower respiratory disease | 0.40 (0.37, 0.43) | 0.53 (0.48, 0.57) | 0.42 (0.36, 0.48) | 0.22 (0.18, 0.25) | 0.35 (0.31, 0.39) | 0.13 (0.10, 0.17) | 0.39 (0.35, 0.44) |
| Osteoporosis | 0.15 (0.12, 0.17) | 0.50 (0.47, 0.54) | 0.10 (0.07, 0.16) | 0.50 (0.46, 0.54) | 0.45 (0.41, 0.48) | 0.14 (0.09, 0.20) | 0.19 (0.16, 0.23) |
| Heart disease | 0.51 (0.48, 0.55) | 0.45 (0.41, 0.50) | 0.55 (0.48, 0.62) | 0.05 (0.02, 0.10) | 0.51 (0.47, 0.56) | 0.15 (0.10, 0.22) | 0.09 (0.06, 0.14) |
| Skin allergies | 0.29 (0.26, 0.32) | 0.43 (0.40, 0.47) | 0.39 (0.34, 0.45) | 0.30 (0.26, 0.33) | 0.25 (0.23, 0.28) | 0.23 (0.19, 0.28) | 0.35 (0.31, 0.39) |
| Eye issues including dry eyes | 0.28 (0.26, 0.32) | 0.57 (0.53, 0.61) | 0.23 (0.18, 0.28) | 0.32 (0.28, 0.36) | 0.34 (0.31, 0.37) | 0.09 (0.06, 0.14) | 0.20 (0.17, 0.24) |
| Diabetes | 0.22 (0.19, 0.27) | 0.24 (0.21, 0.28) | 0.61 (0.55, 0.66) | 0.10 (0.07, 0.13) | 0.25 (0.22, 0.28) | 0.26 (0.22, 0.31) | 0.24 (0.20, 0.29) |
| Anxiety | 0.12 (0.09, 0.14) | 0.51 (0.47, 0.56) | 0.25 (0.20, 0.30) | 0.25 (0.22, 0.29) | 0.17 (0.14, 0.20) | 0.16 (0.12, 0.21) | 0.30 (0.27, 0.34) |
| Cognitive impairment | 0.28 (0.25, 0.31) | 0.28 (0.24, 0.31) | 0.24 (0.19, 0.29) | 0.29 (0.26, 0.32) | 0.25 (0.22, 0.28) | 0.29 (0.24, 0.34) | 0.21 (0.18, 0.24) |
| Cerebrovascular disease & stroke | 0.32 (0.28, 0.35) | 0.27 (0.24, 0.30) | 0.46 (0.41, 0.51) | 0.13 (0.10, 0.16) | 0.30 (0.27, 0.33) | 0.25 (0.20, 0.29) | 0.23 (0.19, 0.27) |
| Arrhythmias | 0.43 (0.39, 0.46) | 0.31 (0.27, 0.35) | 0.30 (0.25, 0.36) | 0.04 (0.02, 0.08) | 0.45 (0.41, 0.49) | 0.12 (0.09, 0.17) | 0.08 (0.06, 0.11) |
| Neoplasms | 0.40 (0.37, 0.44) | 0.25 (0.22, 0.28) | 0.22 (0.18, 0.27) | 0.23 (0.20, 0.26) | 0.25 (0.23, 0.28) | 0.19 (0.15, 0.23) | 0.18 (0.15, 0.22) |
| Mean age at admission, years | 86.16 (85.62, 86.70) | 84.80 (84.22, 85.39) | 75.57 (73.34, 77.80) | 86.25 (85.60, 86.91) | 88.03 (87.61, 88.45) | 80.54 (79.21, 81.87) | 66.05 (65.31, 66.79) |

Supplemental Table 8. Resident Characteristics by Comorbidity Cluster

|  | Cluster 1 | Cluster 2 | Cluster 3 | Cluster 4 | Cluster 5 | Cluster 6 | Cluster 7 | Unassigned |
| --- | --- | --- | --- | --- | --- | --- | --- | --- |
|  | Median | | | | | | | |
| Age at discharge | 89 | 90 | 79 | 92 | 92 | 83 | 71 | 87 |
| Time stayed (all stays), years | 1.54 | 3.51 | 3.07 | 3.13 | 2.26 | 2.54 | 3.75 | 2.65 |
| Length of stay (completed stays only), years | 1.38 | 3.58 | 2.53 | 3.46 | 2.24 | 2.98 | 2.45 | 2.62 |
|  |  |  |  |  |  |  |  |  |
|  |  |  |  |  |  |  |  |  |
|  | % | | | | | | | |
| Died during follow up period | 57.70 | 42.47 | 37.58 | 49.19 | 54.90 | 48.20 | 29.23 | 46.33 |
| *Country of birth* |  |  |  |  |  |  |  |  |
| Australia | 67.41 | 70.63 | 64.9 | 68.6 | 71.88 | 59.92 | 70.00 | 69.39 |
| China | 1.58 | 2.64 | 1.49 | 3.19 | 1.39 | 2.00 | 1.15 | 2.72 |
| Italy | 2.37 | 1.28 | 0.50 | 1.76 | 0.46 | 2.00 | 0.90 | 1.51 |
| UK | 10.29 | 8.51 | 8.61 | 8.93 | 10.14 | 10.52 | 6.15 | 8.47 |
| English primary language | 87.05 | 87.35 | 85.1 | 86.32 | 90.43 | 84.02 | 88.97 | 88.44 |
| *Marital status* |  |  |  |  |  |  |  |  |
| Unknown | 5.40 | 4.97 | 6.79 | 5.34 | 5.86 | 6.66 | 9.49 | 6.80 |
| Single | 7.91 | 6.33 | 17.72 | 6.32 | 4.23 | 10.39 | 32.69 | 8.01 |
| Married | 43.96 | 15.44 | 28.31 | 17.07 | 10.9 | 35.95 | 20.00 | 26.46 |
| Widowed | 35.83 | 64.91 | 28.64 | 63.52 | 74.09 | 35.42 | 12.95 | 46.71 |
| Divorced | 5.40 | 7.38 | 14.4 | 7.04 | 4.46 | 9.45 | 20.38 | 10.58 |
| Separated | 1.51 | 0.98 | 4.14 | 0.72 | 0.46 | 2.13 | 4.49 | 1.44 |
| *Facility remoteness* |  |  |  |  |  |  |  |  |
| Major city | 68.71 | 72.29 | 71.19 | 72.83 | 69.68 | 73.5 | 76.03 | 73.09 |
| Inner regional | 27.99 | 26.20 | 25.66 | 25.73 | 28.23 | 24.5 | 21.67 | 24.64 |
| Outer regional | 3.31 | 1.51 | 3.15 | 1.43 | 2.09 | 2.00 | 2.31 | 2.27 |
| *Health conditions not included in clustering* |  | | | | | | |  |
| Heart failure | 17.48 | 17.47 | 14.74 | 4.89 | 20.46 | 3.60 | 3.46 | 8.99 |
| MI | 10.43 | 6.02 | 9.44 | 1.37 | 7.30 | 2.93 | 2.31 | 4.46 |
| Breast cancer (among females) | 0 | 6.01 | 3.43 | 5.40 | 7.59 | 5.18 | 5.77 | 6.87 |
| Prostate cancer (among males) | 14.46 | 7.96 | 5.50 | 7.69 | 0 | 8.90 | 4.51 | 10.34 |
| Lung cancer | 1.65 | 1.28 | 1.49 | 0.46 | 0.64 | 0.80 | 2.05 | 1.06 |
| Colorectal cancer (CRC) | 6.26 | 3.31 | 2.48 | 3.97 | 4.12 | 1.46 | 1.54 | 3.63 |
| Skin cancer | 15.68 | 9.04 | 9.11 | 9.45 | 9.45 | 5.59 | 5.51 | 8.92 |
| Leukemia or lymphoma | 2.73 | 1.43 | 2.15 | 1.04 | 1.80 | 0.93 | 1.03 | 1.28 |
| Metastatic cancer | 4.03 | 0.83 | 1.49 | 0.91 | 0.70 | 0.80 | 3.08 | 1.59 |
| Thyroid disorder | 9.14 | 23.34 | 12.58 | 17.00 | 21.51 | 11.05 | 12.05 | 16.25 |
| Any CNS disorder | 20.94 | 20.48 | 28.15 | 15.18 | 12.46 | 21.04 | 38.59 | 21.92 |
| Parkinson's disease | 10.14 | 8.58 | 7.45 | 7.10 | 3.71 | 10.39 | 8.85 | 10.36 |
| Motor Neuron Disease (MND) | 0.22 | 0.08 | 0 | 0.26 | 0 | 0 | 0.77 | 0.30 |
| Multiple Sclerosis (MS) | 0 | 0.23 | 0 | 0 | 0.17 | 0.27 | 2.31 | 0.15 |
| Epilepsy | 3.24 | 2.71 | 6.13 | 2.21 | 3.25 | 5.86 | 13.85 | 4.23 |
| Huntington's disease | 0.07 | 0 | 0.33 | 0.20 | 0 | 0.13 | 0.64 | 0 |
| Brain injury | 0.22 | 0.15 | 0.66 | 0.33 | 0.12 | 0.53 | 3.85 | 0.68 |
| Digestive, other | 25.83 | 30.95 | 20.53 | 20.07 | 20.93 | 16.25 | 17.56 | 22.68 |
| Liver disease | 2.01 | 1.88 | 4.30 | 1.37 | 0.93 | 1.07 | 5.77 | 2.27 |
| Diarrhoea | 1.87 | 3.01 | 0.83 | 1.43 | 1.39 | 0.40 | 1.28 | 1.51 |
| Nausea | 15.76 | 37.12 | 21.52 | 17.13 | 21.33 | 6.26 | 17.69 | 18.67 |
| Faecal incontinence | 20.58 | 24.47 | 20.03 | 27.49 | 16.06 | 34.75 | 22.18 | 27.51 |
| Double incontinence | 13.24 | 19.13 | 13.41 | 22.21 | 11.07 | 29.03 | 15.26 | 20.71 |
| Renal disease | 22.81 | 15.06 | 21.69 | 10.23 | 16.99 | 11.32 | 10.00 | 13.08 |
| Urinary tract infection (UTI) | 7.84 | 20.03 | 9.93 | 17.92 | 19.07 | 12.38 | 8.46 | 16.10 |
| Other mental/behavioural condition | 5.25 | 2.48 | 9.11 | 1.95 | 0.87 | 6.66 | 18.46 | 6.05 |
| Schizophrenia or bi-polar disorder | 4.17 | 7.45 | 13.58 | 7.49 | 3.83 | 11.45 | 31.03 | 9.45 |
| Delirium | 7.41 | 5.57 | 5.13 | 6.71 | 8.23 | 4.13 | 2.44 | 5.82 |
| Rheumatoid arthritis (RA) | 2.16 | 5.27 | 2.65 | 3.39 | 3.59 | 3.59 | 1.79 | 2.49 |
| Gout | 21.87 | 14.16 | 17.05 | 4.95 | 10.72 | 5.19 | 5.90 | 8.24 |
| Fracture | 20.36 | 35.54 | 14.9 | 34.92 | 31.07 | 18.91 | 18.59 | 27.36 |
| Amputation | 2.01 | 1.13 | 4.97 | 0.98 | 1.39 | 1.07 | 2.18 | 1.06 |
| Back pain | 7.91 | 15.36 | 6.13 | 10.75 | 10.26 | 5.06 | 6.03 | 7.86 |
| Visual impairment | 17.27 | 21.46 | 11.75 | 19.09 | 21.16 | 12.12 | 11.79 | 15.65 |
| Cataracts | 12.01 | 18.22 | 8.94 | 15.96 | 14.55 | 11.19 | 7.44 | 13.00 |
| Retinopathy | 9.50 | 15.66 | 6.29 | 12.96 | 15.07 | 5.06 | 2.18 | 8.39 |
| Glaucoma | 16.98 | 17.09 | 10.26 | 16.22 | 19.3 | 10.65 | 7.05 | 16.18 |
| Hearing impairment | 30.07 | 27.41 | 13.58 | 27.10 | 29.16 | 19.97 | 9.10 | 21.77 |
| Skin, other condition | 3.24 | 4.22 | 2.65 | 4.04 | 2.90 | 2.93 | 3.08 | 4.23 |
| Wound | 5.47 | 7.68 | 4.97 | 3.45 | 7.65 | 3.20 | 3.33 | 5.74 |
| Skin infection | 5.83 | 8.28 | 6.95 | 4.36 | 5.97 | 3.06 | 5.00 | 4.61 |

Supplemental Table 9. ACFI domains: Functional and cognitive ratings by cluster

|  | **Cluster 1** | **Cluster 2** | **Cluster 3** | **Cluster 4** | **Cluster 5** | **Cluster 6** | **Cluster 7** |
| --- | --- | --- | --- | --- | --- | --- | --- |
| ADL score, median (IQR) | 83.42 (64.65, 90.31) | 83.61 (70.03, 90.31) | 83.42 (64.33, 90.31) | 90.31 (76.72, 93.29) | 90.12 (66.11, 90.31) | 90.12 (76.54, 97.01) | 83.42 (64.33, 90.31) |
| Nutrition |  |  |  |  |  |  |  |
| 0 | 2.92% | 3.00% | 3.06% | 1.67% | 2.81% | 0.68% | 1.97% |
| 1 | 22.63% | 17.88% | 23.90% | 13.78% | 20.56% | 16.21% | 21.63% |
| 2 | 57.12% | 67.73% | 60.80% | 55.81% | 59.38% | 46.42% | 55.82% |
| 3 | 17.33% | 11.39% | 12.24% | 28.74% | 17.24% | 36.69% | 20.57% |
| Mobility |  |  |  |  |  |  |  |
| 0 | 1.41% | 0.50% | 1.92% | 0.84% | 0.65% | 1.71% | 3.18% |
| 1 | 2.83% | 1.10% | 4.21% | 2.26% | 2.53% | 3.93% | 5.45% |
| 2 | 45.00% | 45.21% | 46.17% | 38.93% | 44.66% | 45.81% | 46.14% |
| 3 | 50.75% | 53.19% | 47.70% | 57.98% | 52.16% | 48.55% | 45.23% |
| Hygiene |  |  |  |  |  |  |  |
| 0 | 0.09% | 0.10% | 0.38% | 0.08% | 0.07% | 0.00% | 0.15% |
| 1 | 11.69% | 5.50% | 11.47% | 4.86% | 8.59% | 7.01% | 12.71% |
| 2 | 12.67% | 15.50% | 15.87% | 8.88% | 14.29% | 6.67% | 10.14% |
| 3 | 75.55% | 78.90% | 72.28% | 86.18% | 77.06% | 86.32% | 77.00% |
| Toileting |  |  |  |  |  |  |  |
| 0 | 3.27% | 1.50% | 3.06% | 0.92% | 2.60% | 1.54% | 4.39% |
| 1 | 17.42% | 13.37% | 20.46% | 10.53% | 14.36% | 10.41% | 20.00% |
| 2 | 16.45% | 20.06% | 15.49% | 13.70% | 17.60% | 16.89% | 16.52% |
| 3 | 62.86% | 65.07% | 60.99% | 74.85% | 65.44% | 71.16% | 59.09% |
| Continence |  |  |  |  |  |  |  |
| 0 | 20.25% | 14.27% | 21.22% | 9.19% | 14.94% | 9.73% | 21.48% |
| 1 | 3.80% | 2.10% | 2.87% | 1.59% | 2.45% | 0.85% | 2.42% |
| 2 | 6.01% | 5.39% | 5.16% | 3.76% | 5.19% | 3.58% | 5.30% |
| 3 | 69.94% | 78.24% | 70.75% | 85.46% | 77.42% | 85.84% | 70.80% |
| Cognition |  |  |  |  |  |  |  |
| 0 | 10.96% | 13.67% | 14.53% | 4.93% | 12.12% | 1.02% | 11.04% |
| 1 | 37.31% | 41.62% | 40.34% | 20.72% | 34.20% | 12.29% | 34.19% |
| 2 | 26.35% | 30.24% | 24.47% | 30.33% | 29.37% | 29.35% | 26.93% |
| 3 | 25.38% | 14.47% | 20.65% | 44.03% | 24.31% | 57.34% | 27.84% |
| Wandering |  |  |  |  |  |  |  |
| 0 | 77.52% | 83.82% | 76.67% | 72.77% | 80.38% | 56.14% | 62.33% |
| 1 | 6.46% | 5.29% | 7.84% | 6.77% | 5.70% | 7.68% | 9.53% |
| 2 | 4.87% | 3.80% | 3.63% | 5.10% | 4.62% | 6.48% | 7.72% |
| 3 | 11.15% | 7.09% | 11.85% | 15.37% | 9.31% | 29.69% | 20.42% |
| Verbal behaviour |  |  |  |  |  |  |  |
| 0 | 11.58% | 8.89% | 8.80% | 8.10% | 12.05% | 10.24% | 9.08% |
| 1 | 15.74% | 13.19% | 12.43% | 12.20% | 15.44% | 11.60% | 9.98% |
| 2 | 19.27% | 22.68% | 19.31% | 19.05% | 21.14% | 13.31% | 14.67% |
| 3 | 53.40% | 55.24% | 59.46% | 60.65% | 51.37% | 64.85% | 66.26% |
| Physical behaviour |  |  |  |  |  |  |  |
| 0 | 25.93% | 23.45% | 24.28% | 16.79% | 30.23% | 11.77% | 12.56% |
| 1 | 18.32% | 15.87% | 17.21% | 14.70% | 18.25% | 11.43% | 12.56% |
| 2 | 14.87% | 20.06% | 14.91% | 19.05% | 16.52% | 15.36% | 16.79% |
| 3 | 40.88% | 40.62% | 43.59% | 49.46% | 34.99% | 61.43% | 58.09% |
| Depression |  |  |  |  |  |  |  |
| 0 | 28.74% | 15.87% | 20.84% | 29.41% | 34.05% | 34.47% | 26.48% |
| 1 | 44.47% | 31.84% | 34.23% | 41.10% | 47.04% | 47.95% | 39.49% |
| 2 | 11.49% | 22.26% | 21.03% | 14.62% | 9.67% | 8.02% | 13.92% |
| 3 | 15.30% | 30.04% | 23.90% | 14.87% | 9.24% | 9.56% | 20.12% |
| Medication |  |  |  |  |  |  |  |
| 0 | 2.48% | 1.20% | 2.68% | 2.34% | 2.38% | 1.03% | 2.12% |
| 1 | 23.87% | 13.87% | 12.43% | 24.23% | 21.50% | 32.14% | 20.42% |
| 2 | 31.12% | 20.46% | 24.86% | 28.65% | 31.67% | 30.26% | 26.78% |
| 3 | 42.53% | 64.47% | 60.04% | 44.78% | 44.44% | 36.58% | 50.68% |
| Complex healthcare |  |  |  |  |  |  |  |
| 0 | 9.99% | 5.69% | 7.84% | 10.53% | 9.24% | 18.12% | 15.89% |
| 1 | 19.10% | 14.97% | 19.12% | 19.05% | 17.60% | 21.03% | 22.69% |
| 2 | 39.70% | 48.40% | 43.21% | 43.11% | 43.94% | 38.46% | 37.52% |
| 3 | 31.21% | 30.94% | 29.83% | 27.32% | 29.22% | 22.39% | 23.90% |

All ratings are from the first aged care funding instrument (ACFI) assessment recorded. ADL = activities of daily living. For each domain, a higher score indicates a higher degree of impairment. For more detail on the scales used please see the ACFI user guide available at: https://agedcare.health.gov.au/funding/aged-care-subsidies-and-supplements/residential-care-subsidy/basic-subsidy-amount-aged-care-funding-instrument/aged-care-funding-instrument-acfi-user-guide.


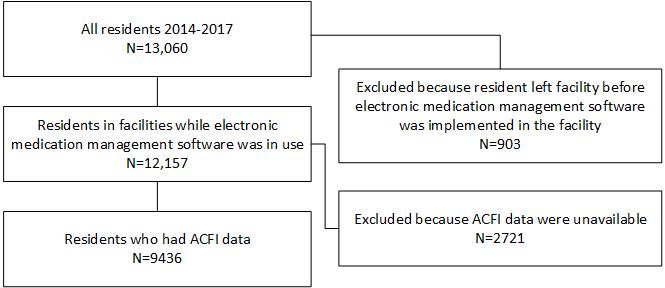


**Supplemental Figure 1. Sample size and exclusions**

**Supplemental Figure 3. Kappa estimates for agreement between conditions recorded in the aged care funding instrument (ACFI) and the electronic health record (EHR)**

Supplemental Figure 6 for data.


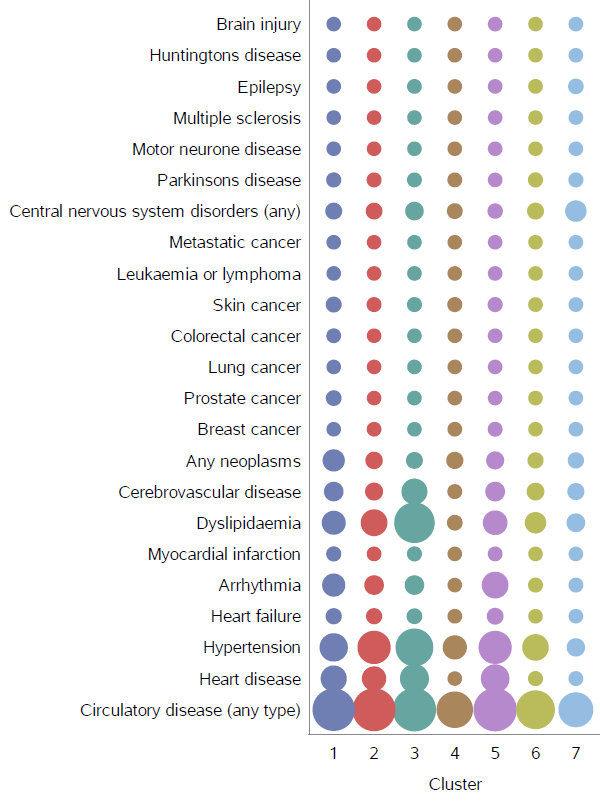

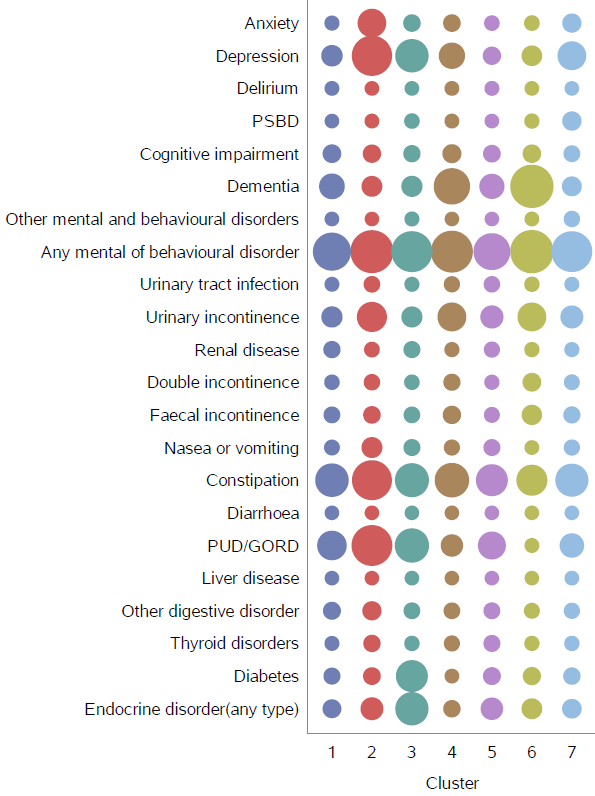

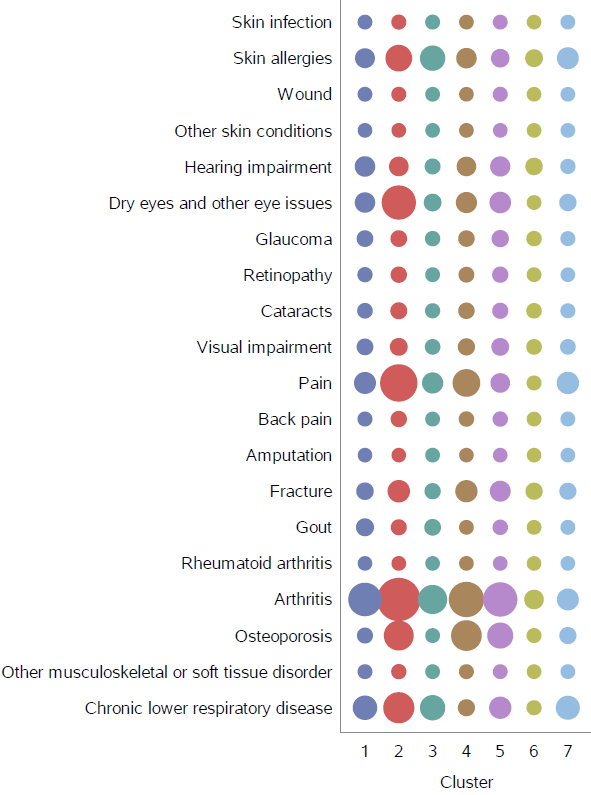


**Supplemental Figure 4. Condition Prevalence by Comorbidity Cluster**
