# Supplementary material for: Measuring the prevalence of 60 health conditions in older Australians in residential aged care with electronic health records: a retrospective dynamic cohort study
Source: Popul Health Metr. 2020 Oct 8;18:25. doi: 10.1186/s12963-020-00234-z (PMC7545887; doi:10.1186/s12963-020-00234-z)
Supplement: Supplementary file 2 — Additional file 2: Supplemental Figure 1. Sample size and exclusions. Supplemental Figure 2. Age and sex specific prevalence estimates, Supplemental Figure 3. Kappa estimates for agreement between conditions recorded in the aged care funding instrument (ACFI) and the electronic health record (EHR). Supplemental Figure 4. Condition Prevalence by Comorbidity Cluster [file 12963_2020_234_MOESM2_ESM.docx]

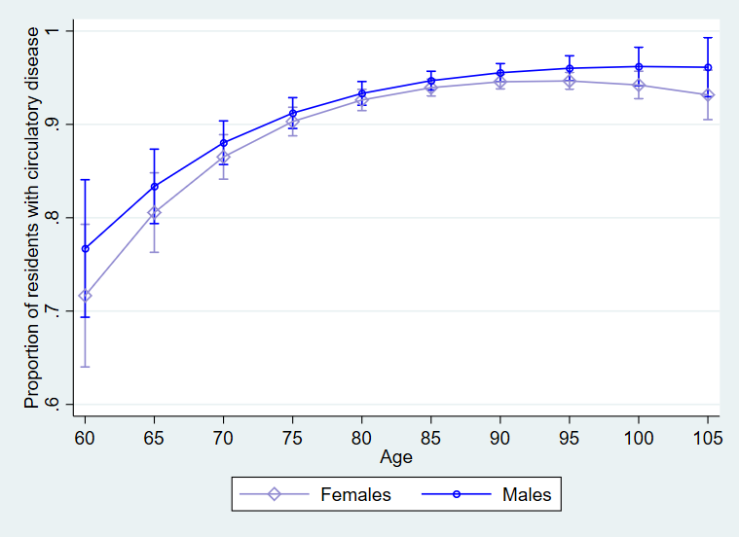

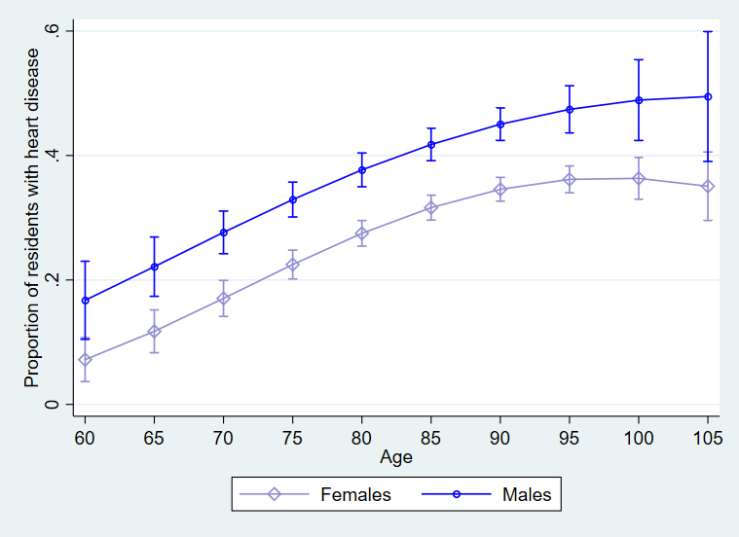


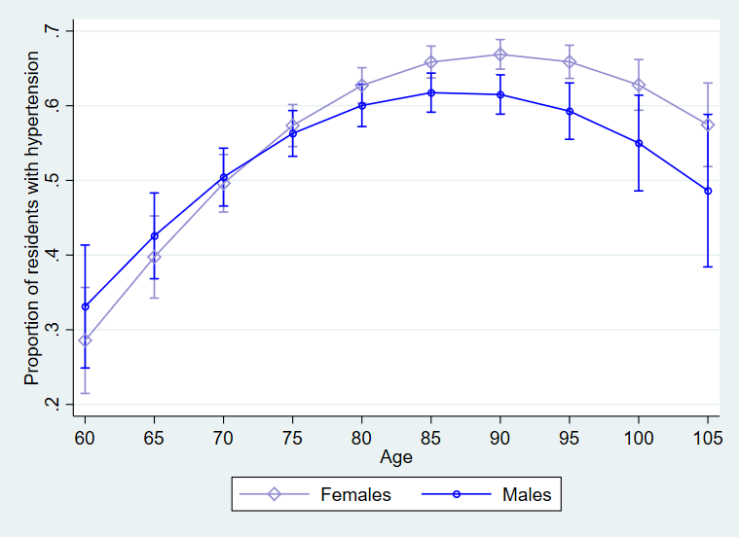

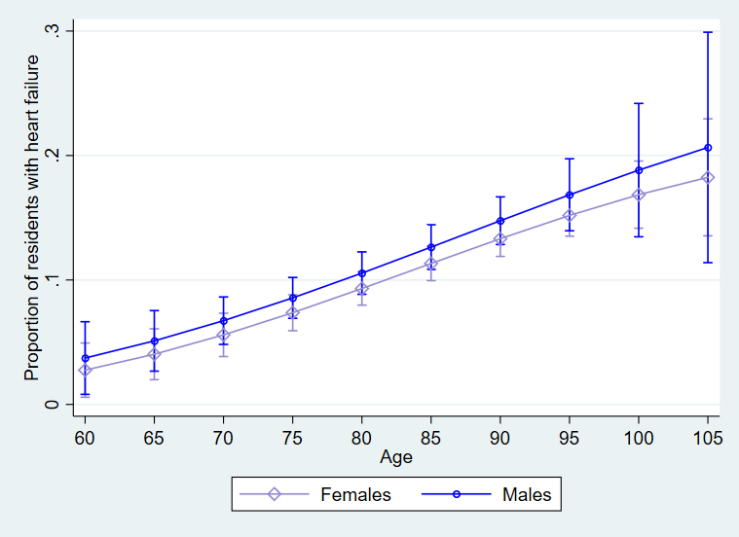


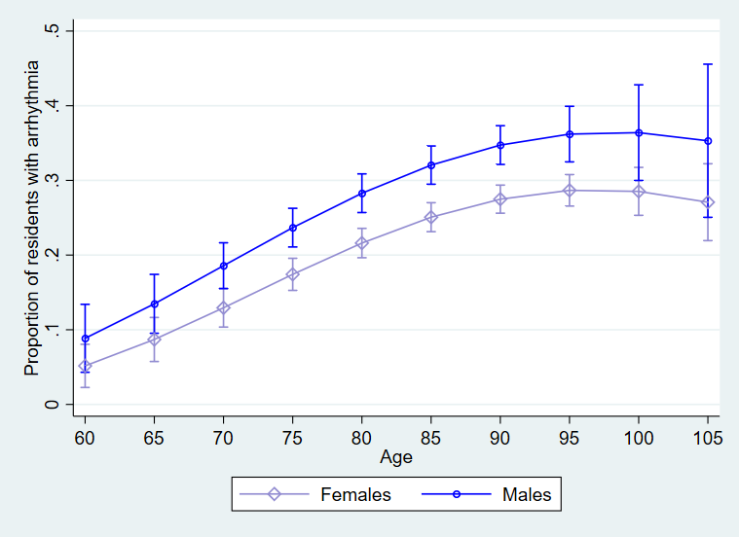

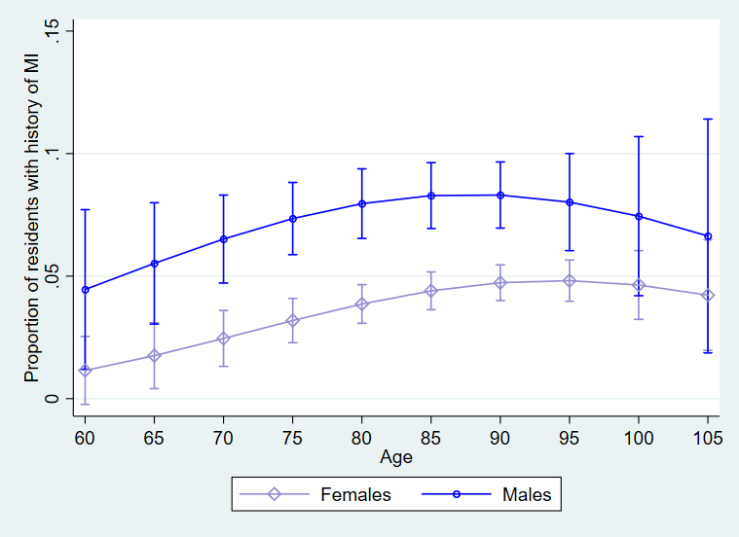


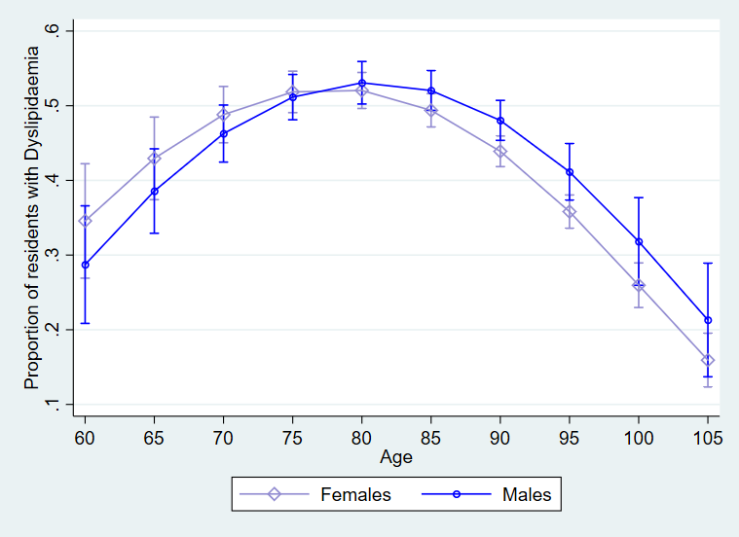

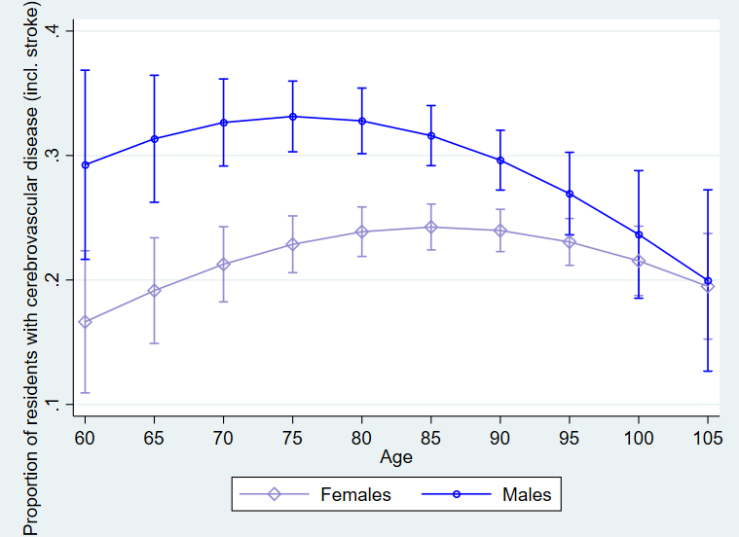


Supplemental Figure 2A. Proportion of Residents with Circulatory Diseases by Age and Sex


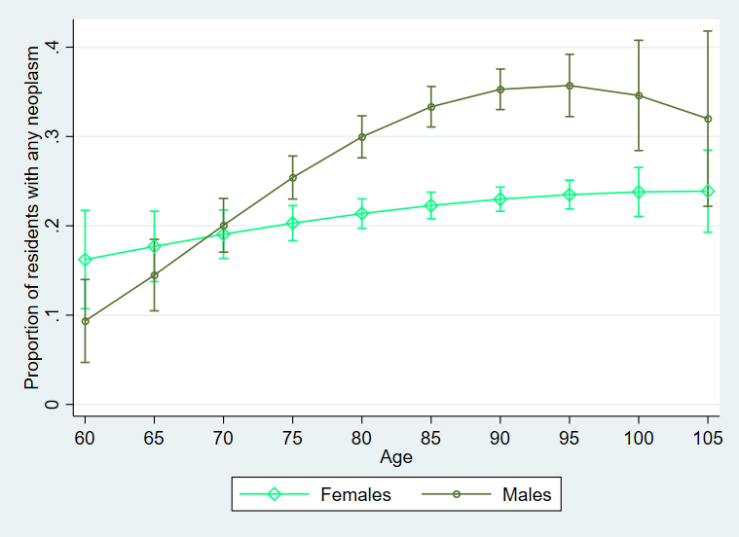

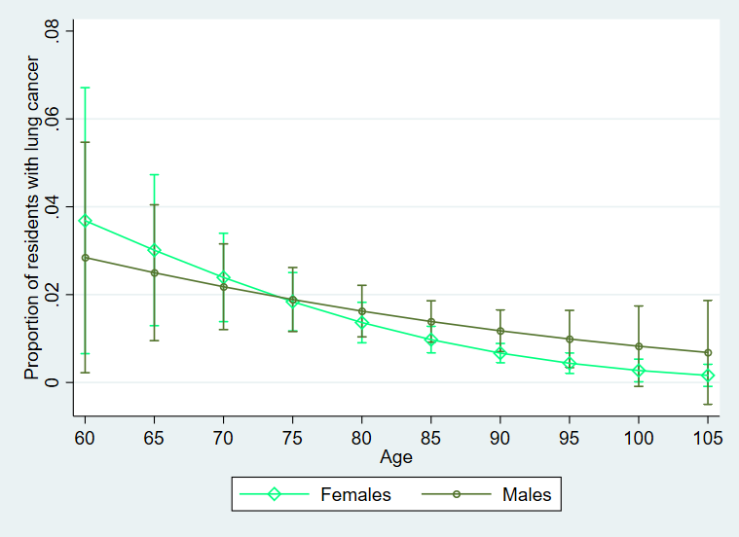


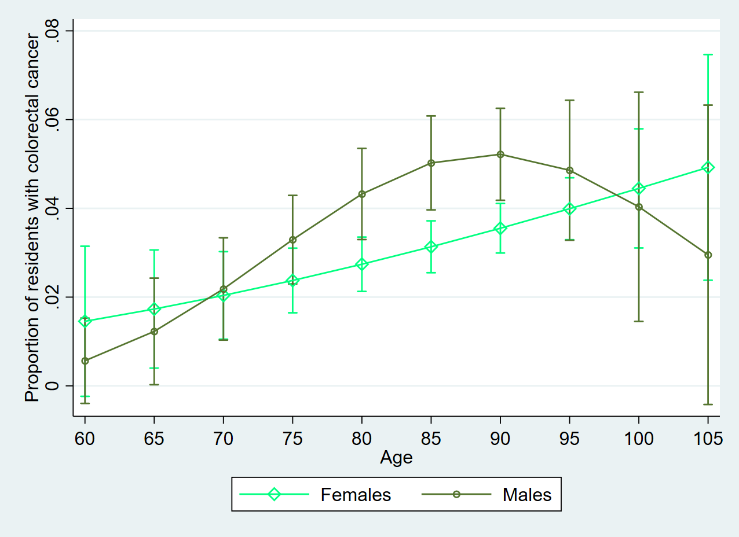

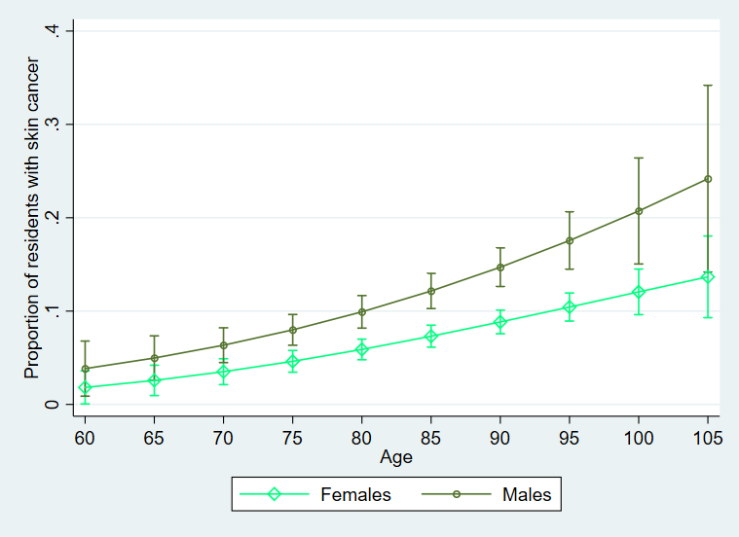


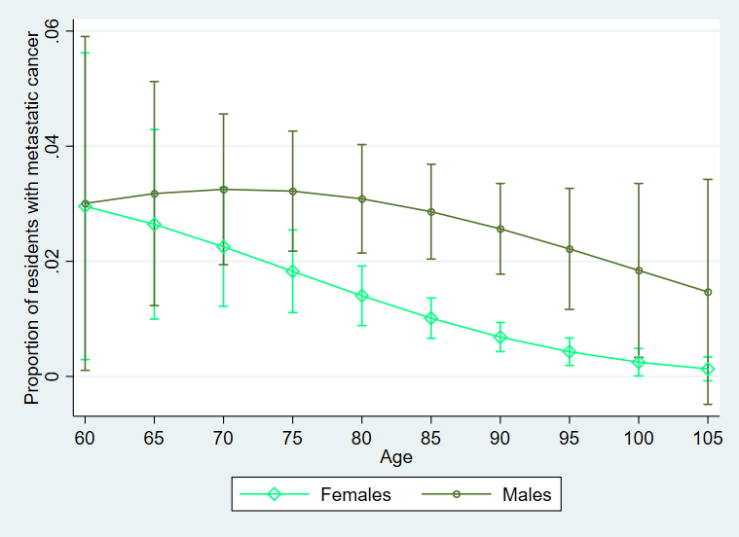


Supplemental Figure 2B. Proportion of Residents with Neoplasms by Age and Sex


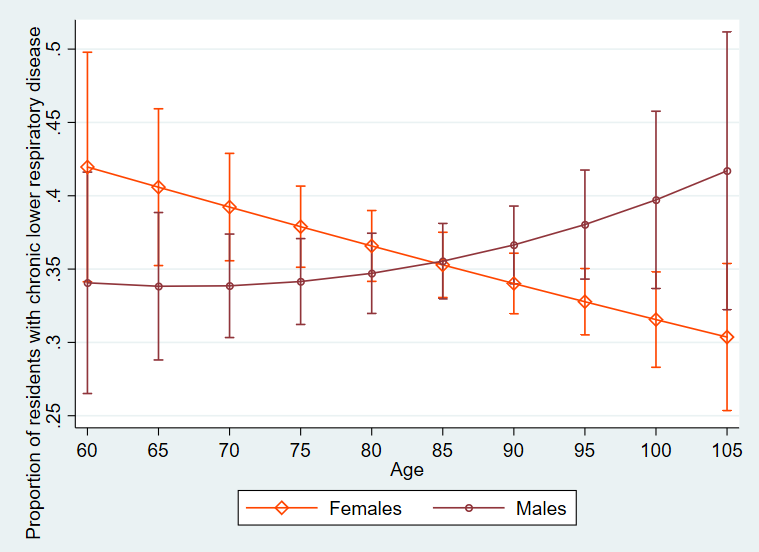


Supplemental Figure 2C. Proportion of Residents with Chronic Lower Respiratory Disease by Age and Sex


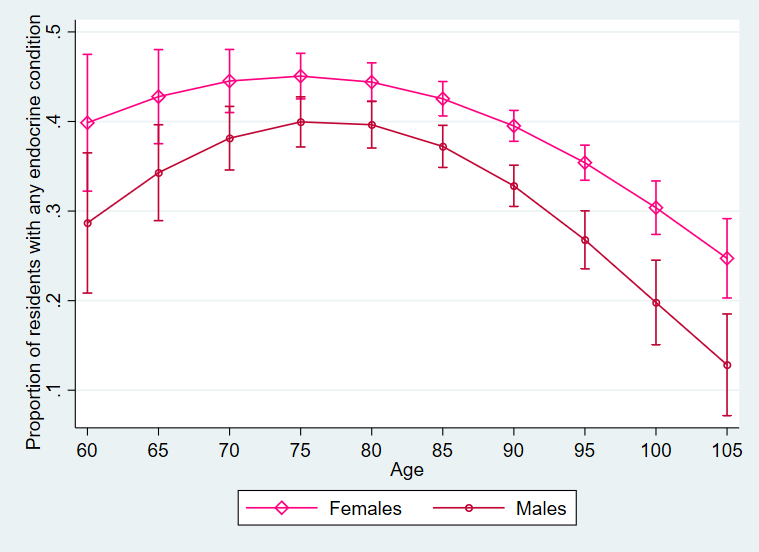


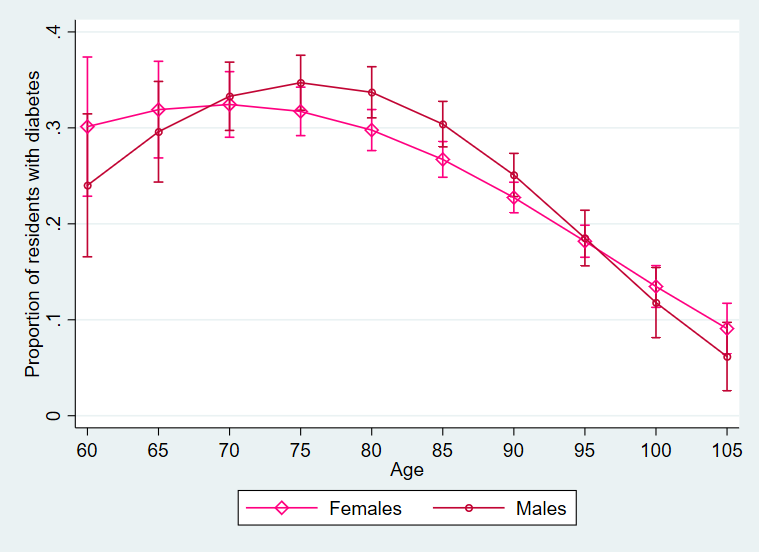


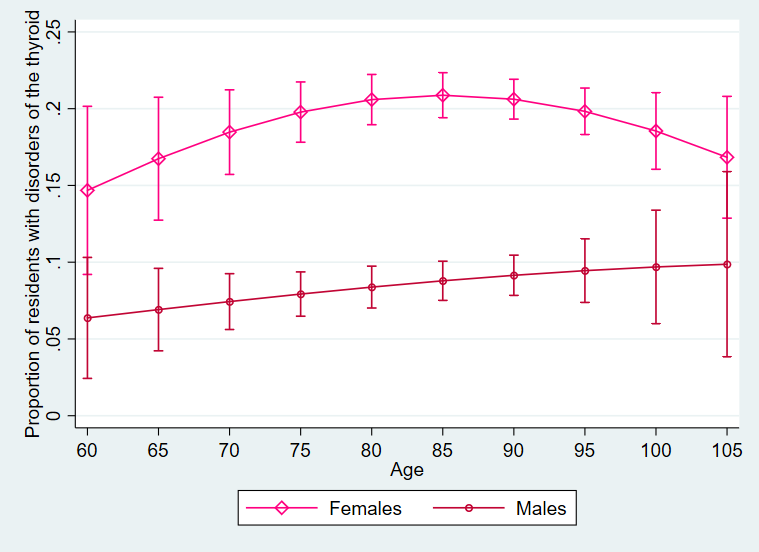


Supplemental Figure 2D. Proportion of Residents with Endocrine Disorders by Age and Sex


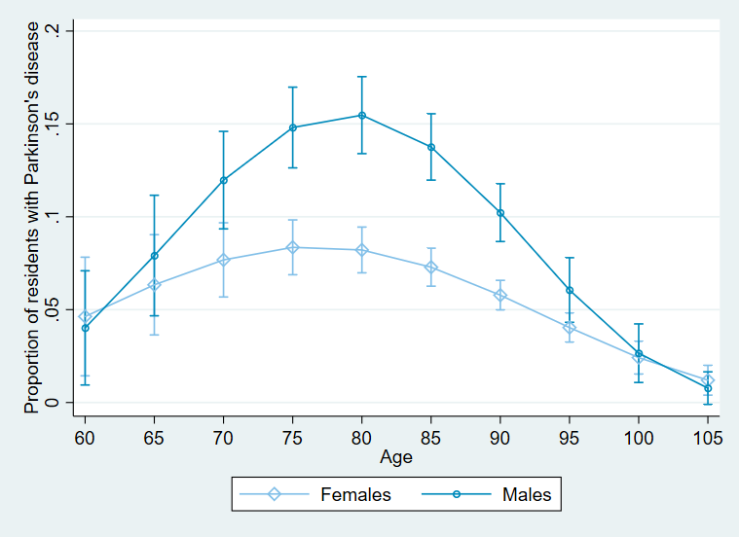

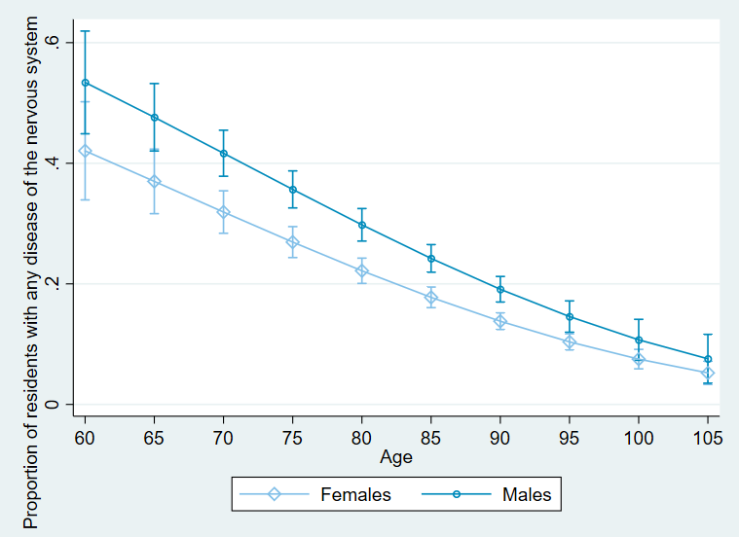


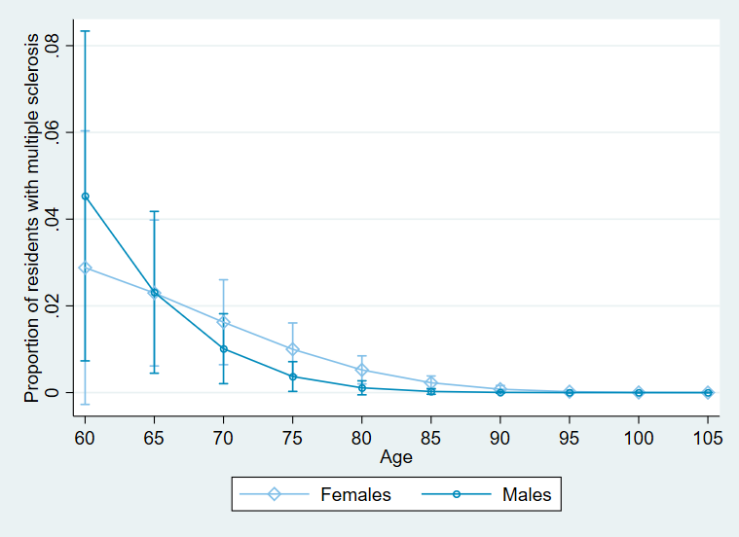

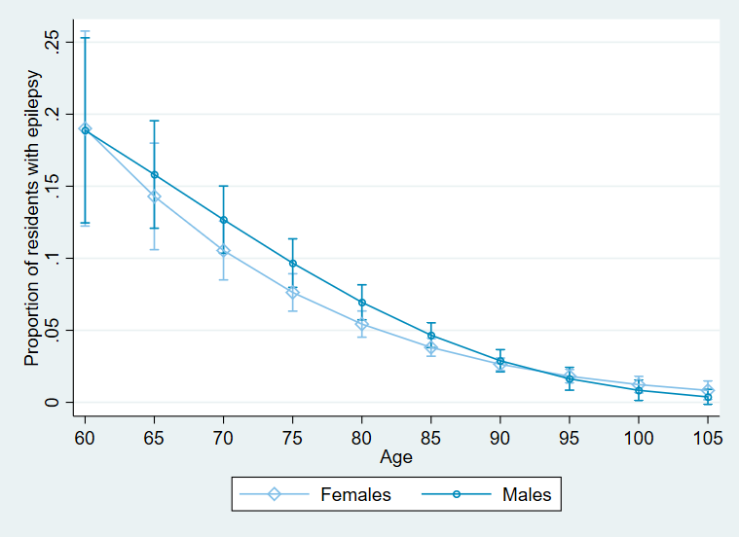


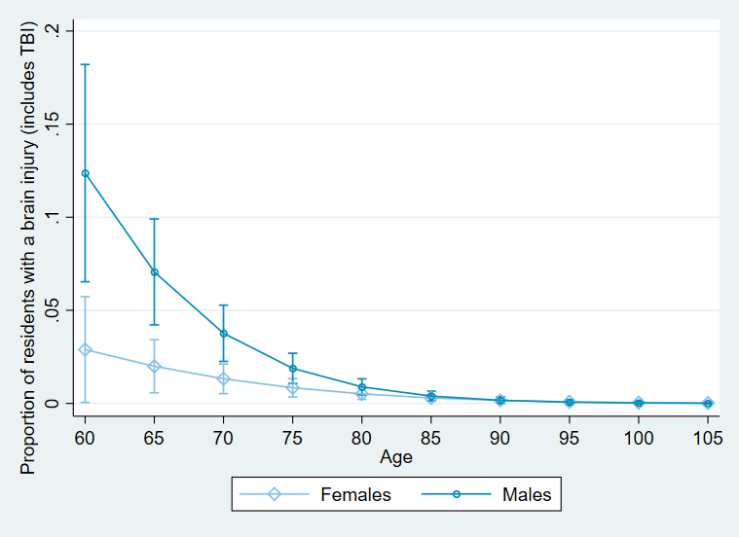

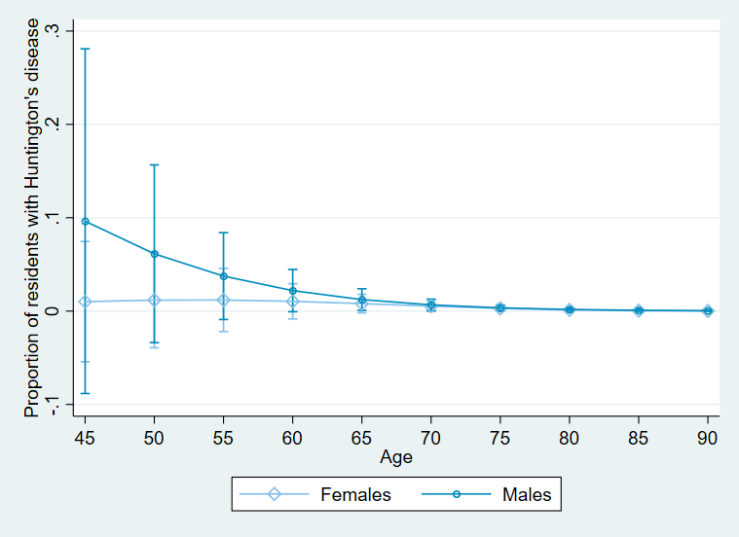


Supplemental Figure 2E. Proportion of Residents with Nervous System Disorders by Age and Sex


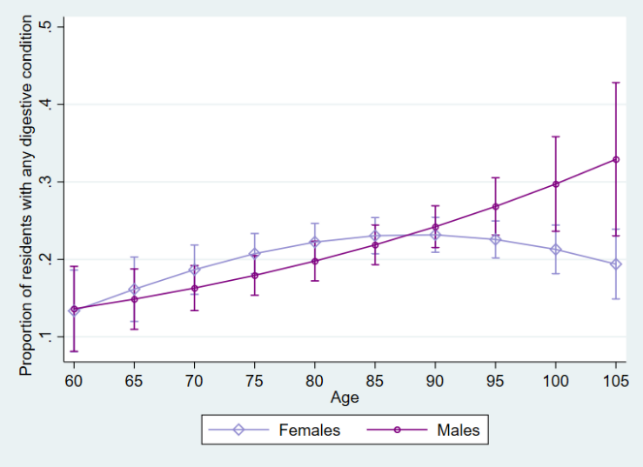

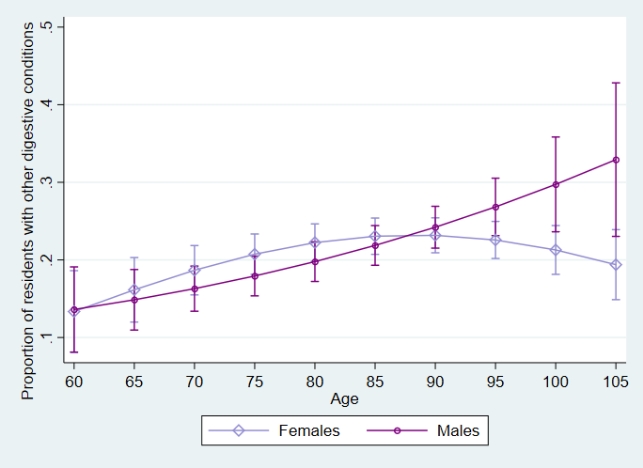


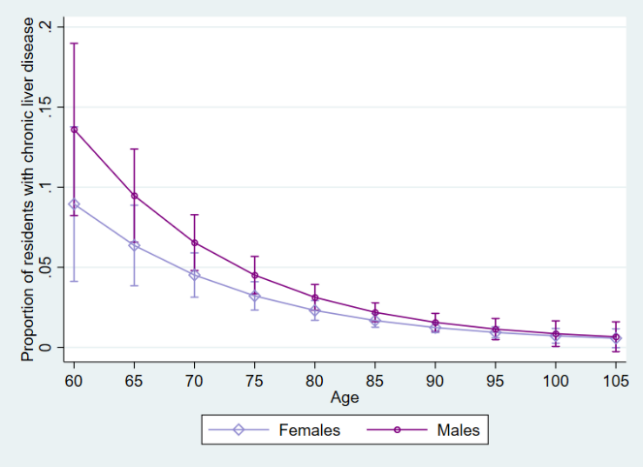

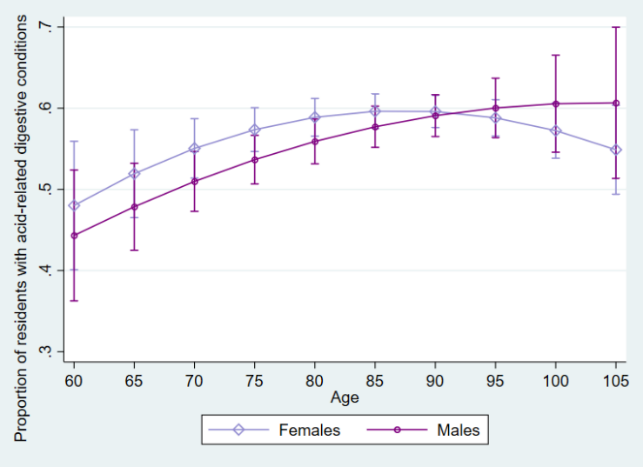


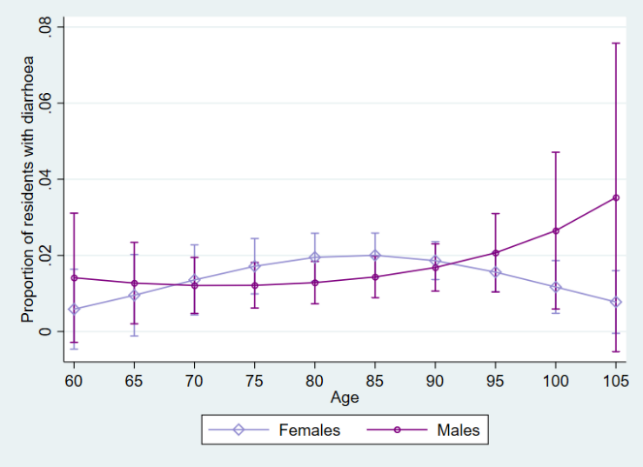

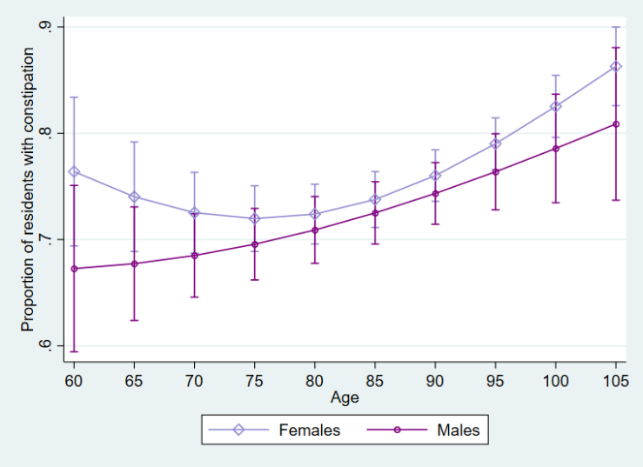


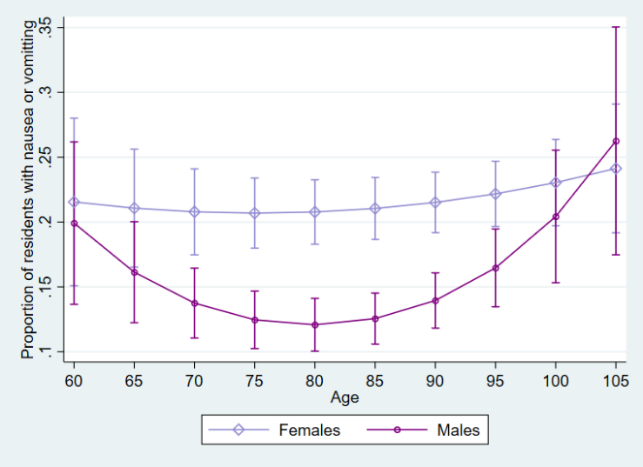

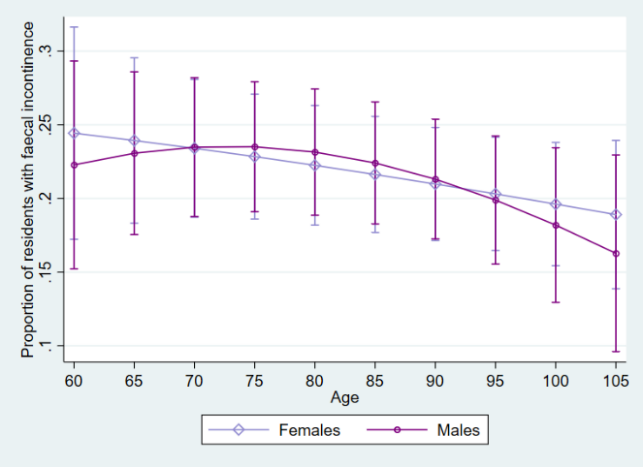


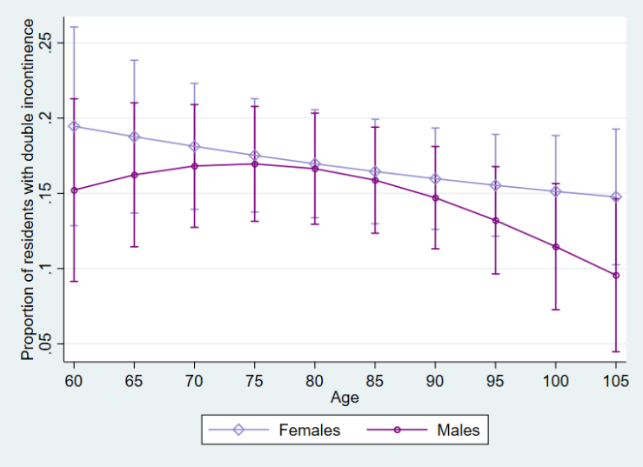


Supplemental Figure 2F. Proportion of Residents with Digestive Disorders by Age and Sex


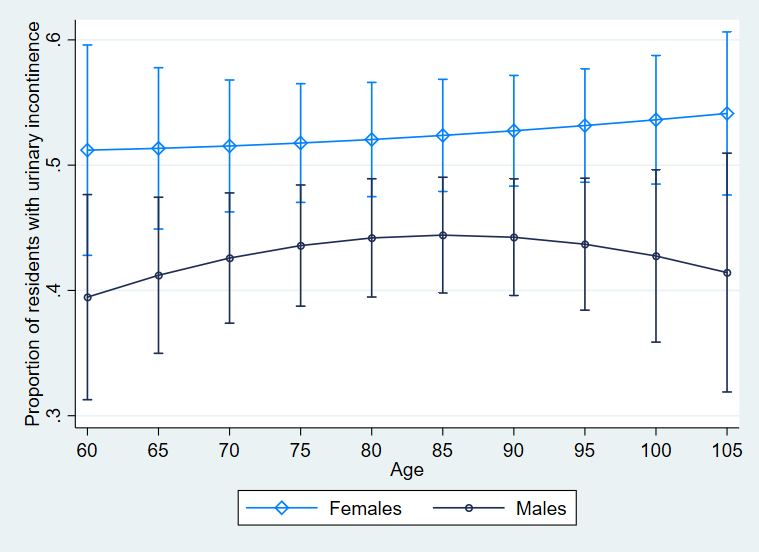


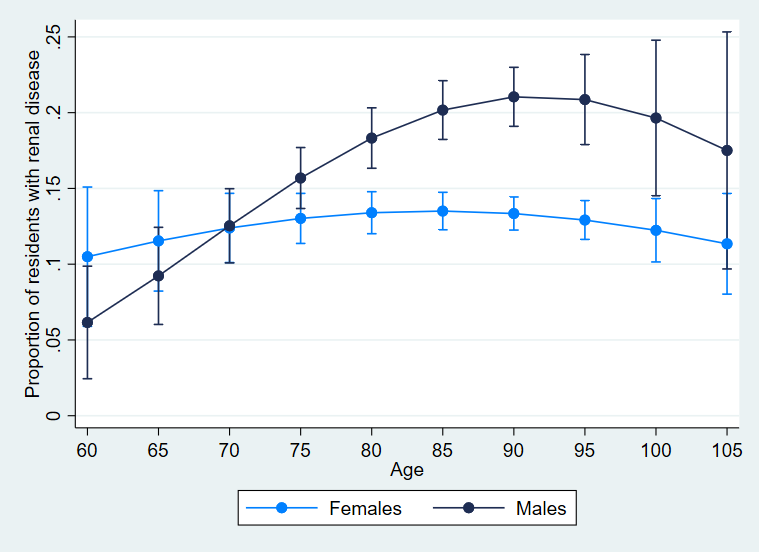


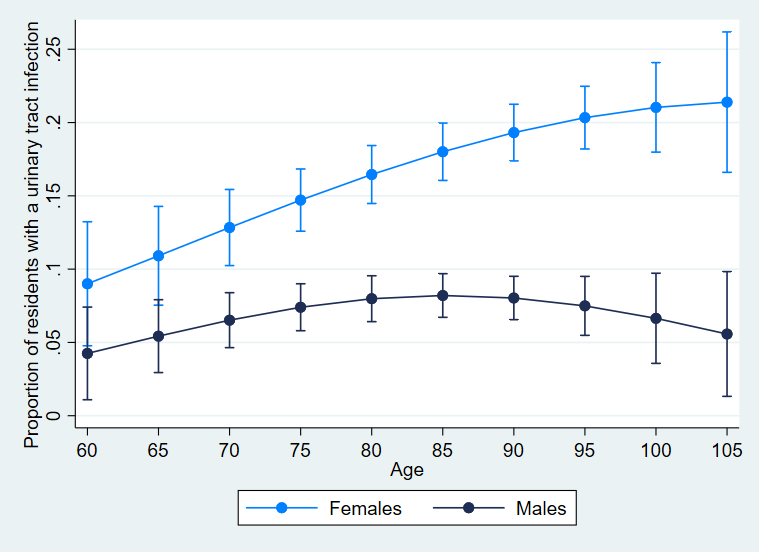


Supplemental Figure 2G. Proportion of Residents with Urinary Disorders by Age and Sex


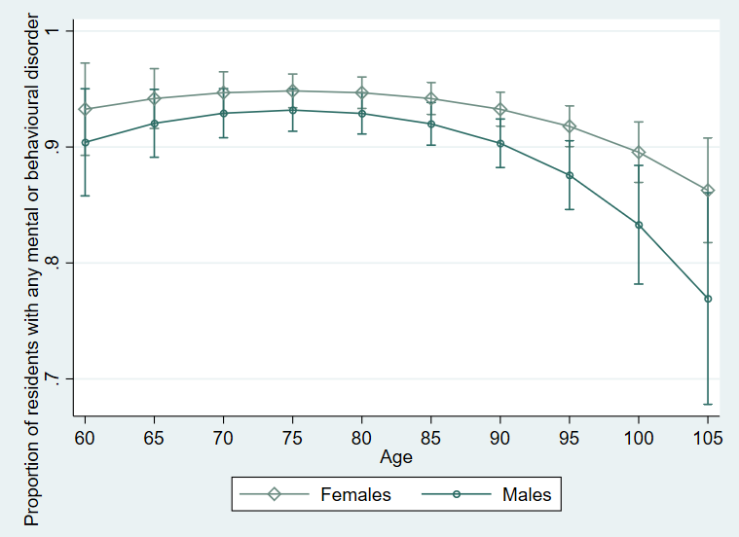

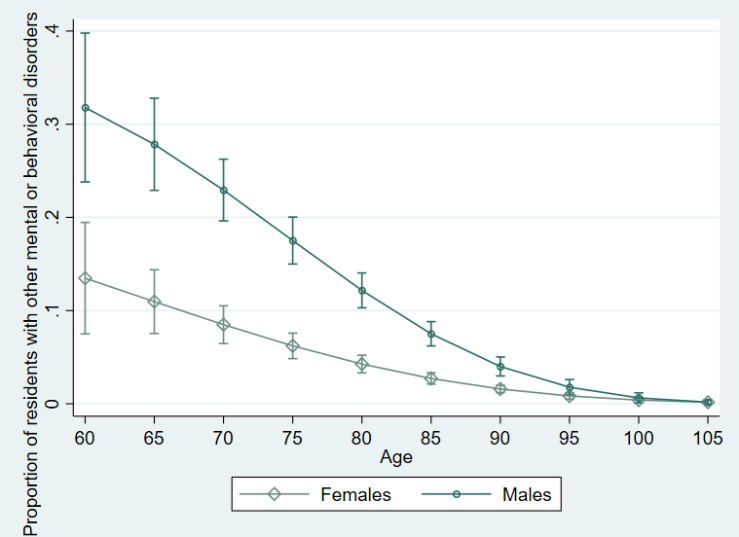


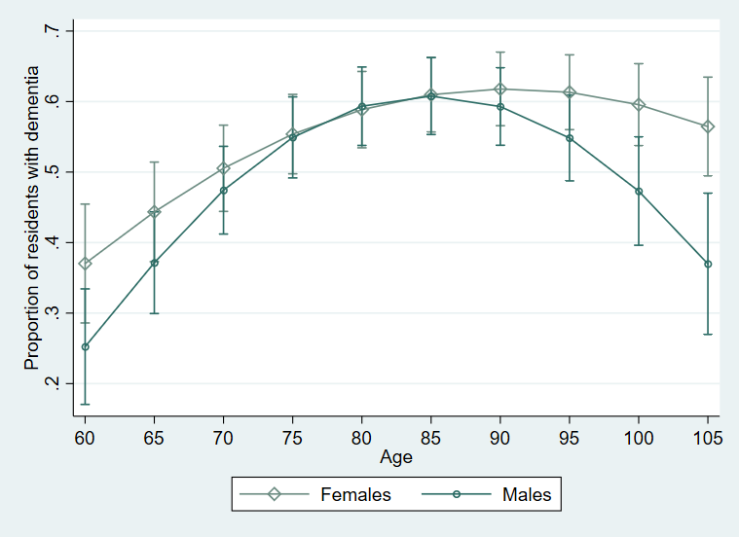

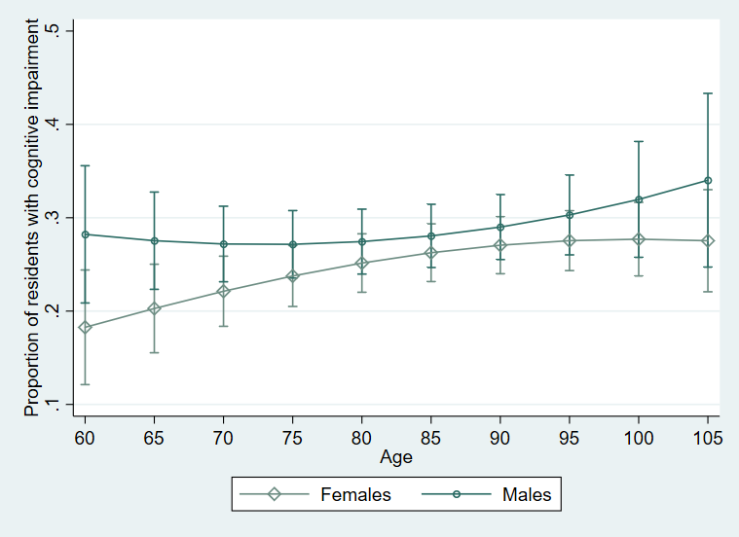


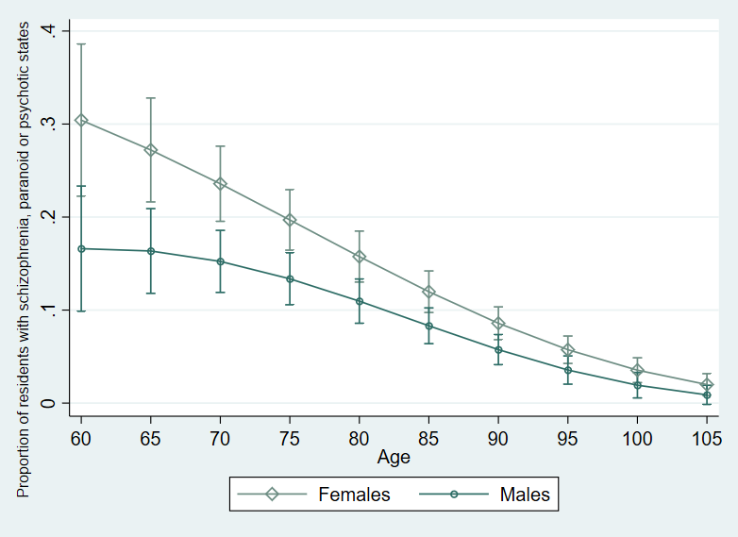

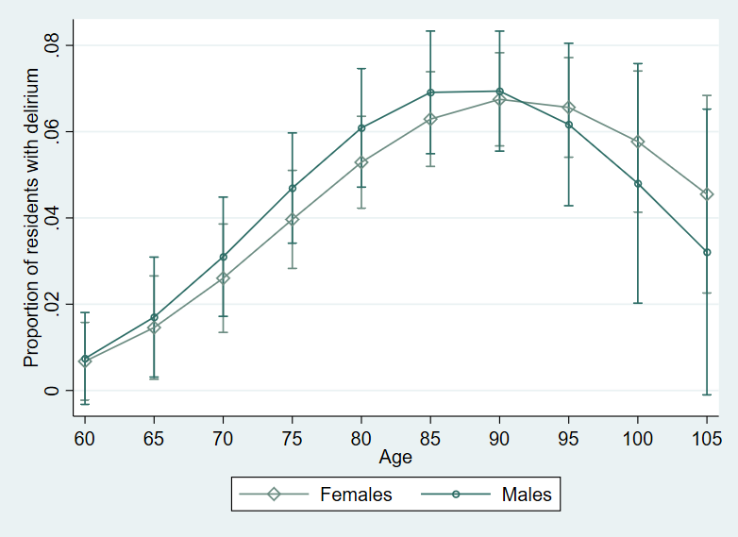


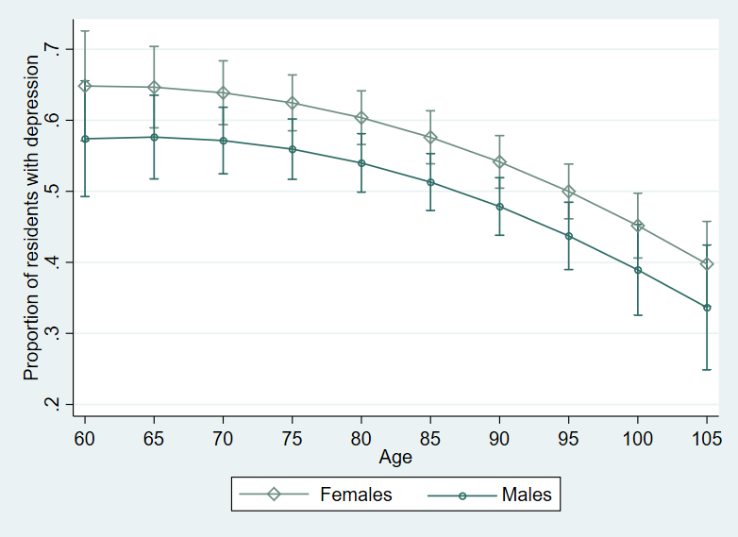

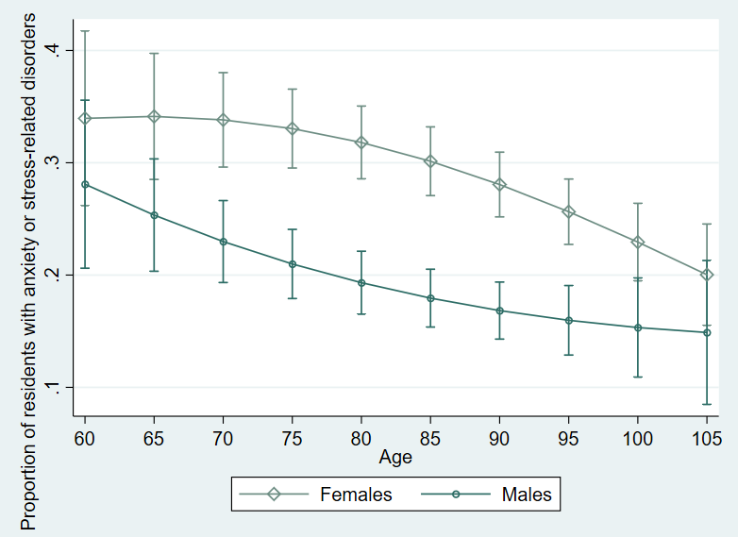


Supplemental Figure 2H. Proportion of Residents with Mental and Behavioural Disorders by Age and Sex


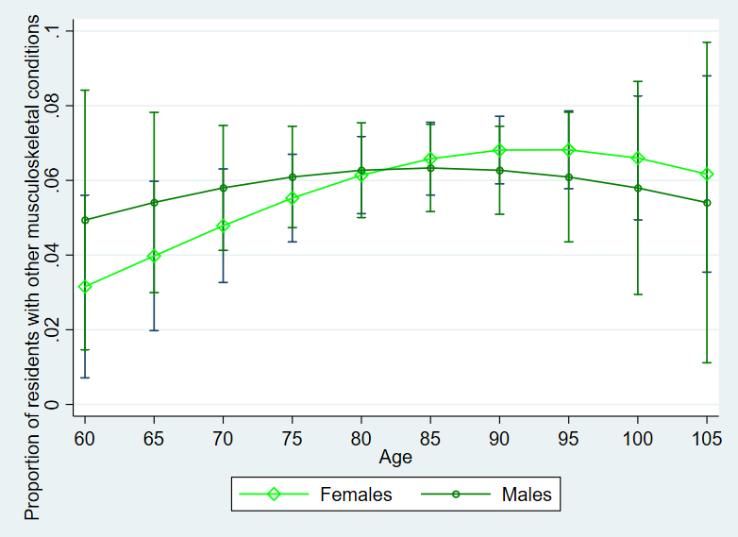

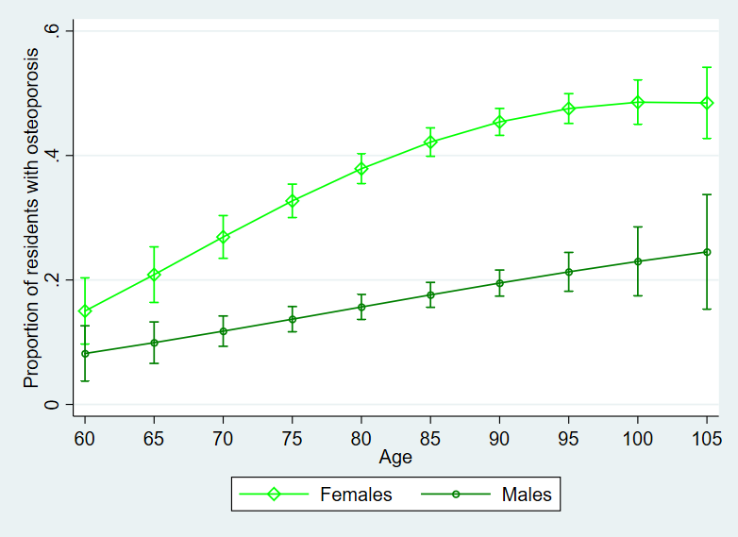


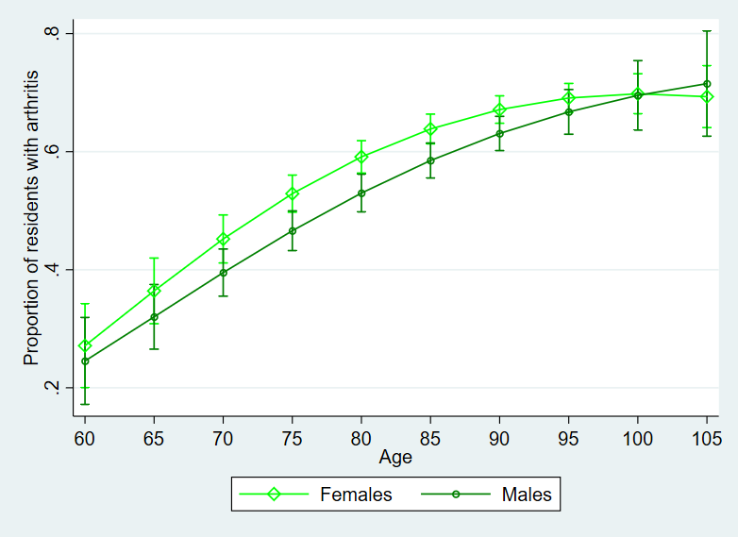

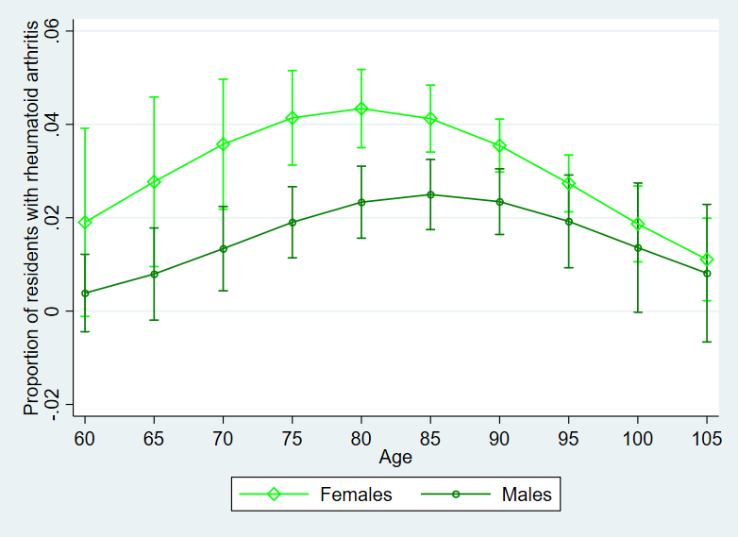


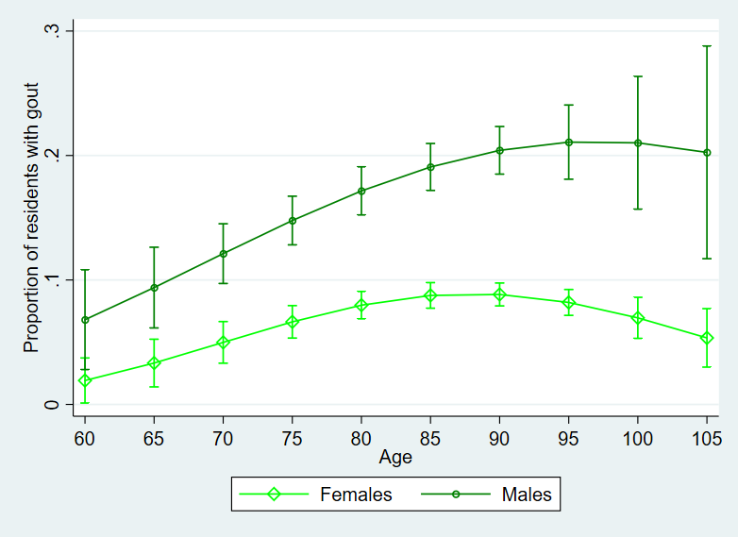

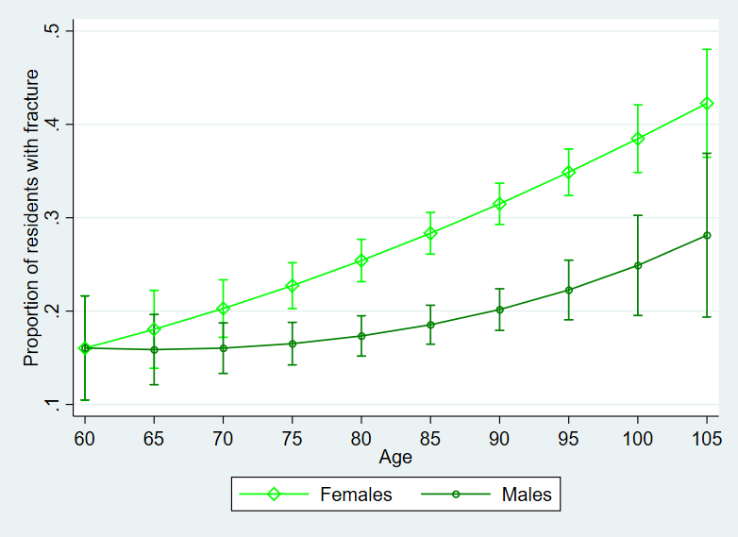


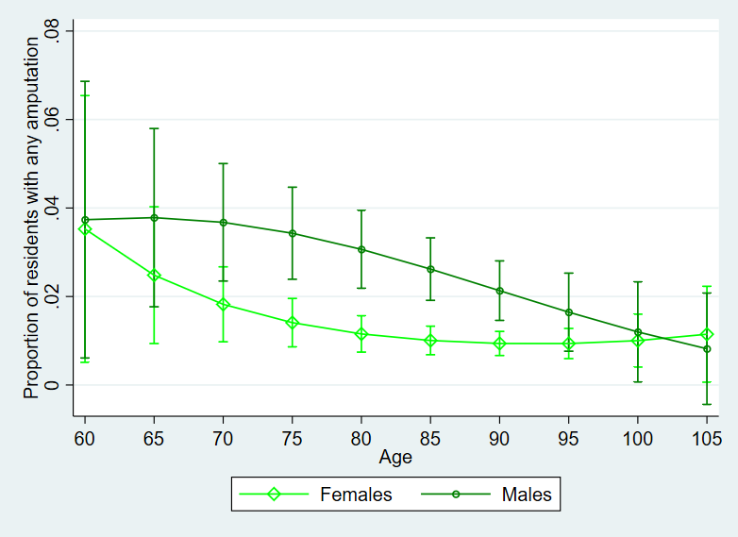

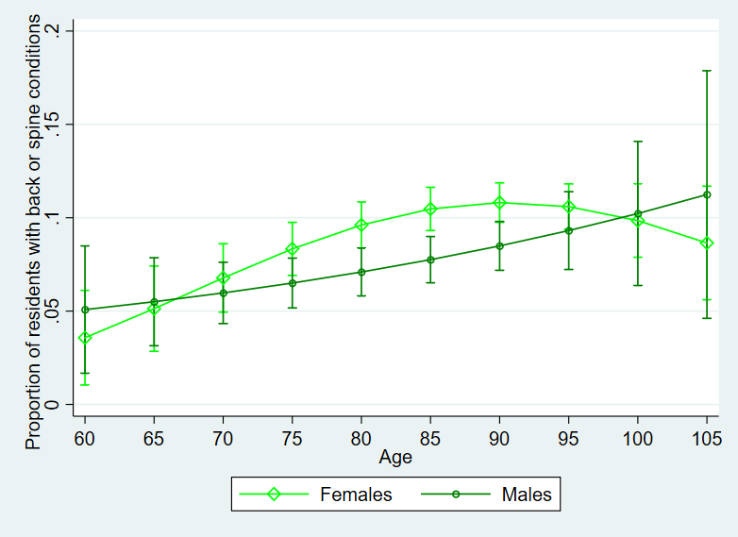


Supplemental Figure 2I. Proportion of Residents with Musculoskeletal Disorders by Age and Sex


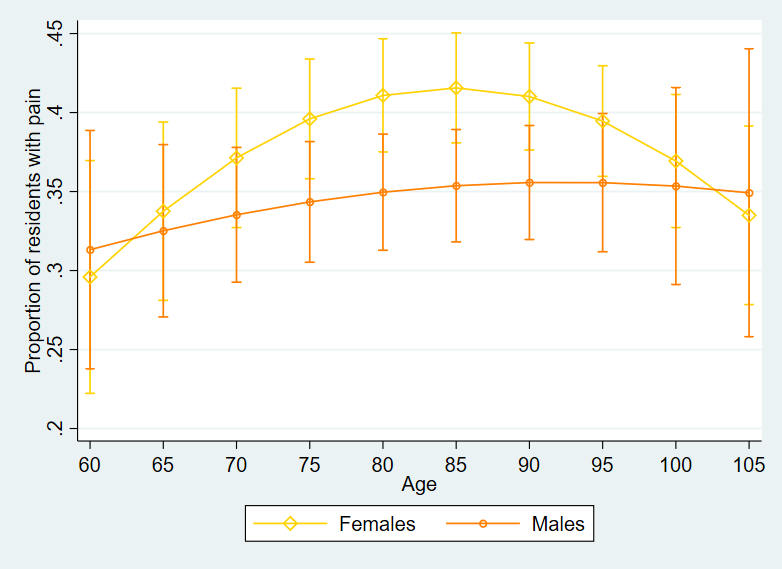


Supplemental Figure 2J. Proportion of Residents with Pain Syndromes or Chronic Pain NOS by Age and Sex


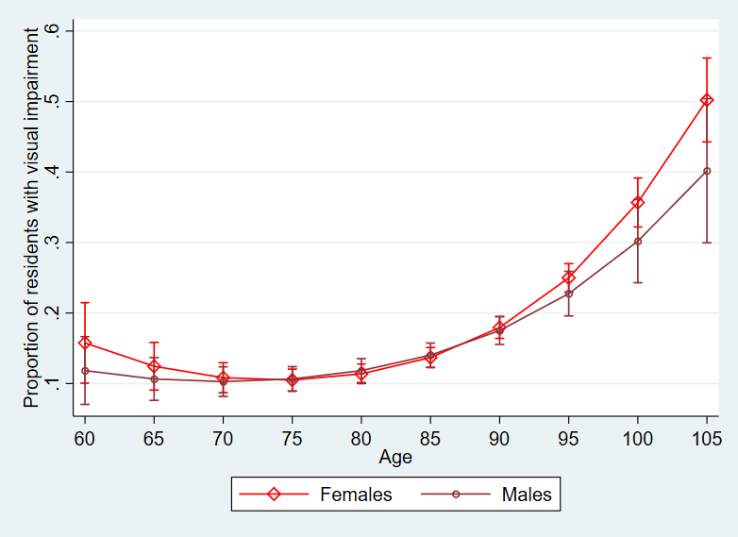

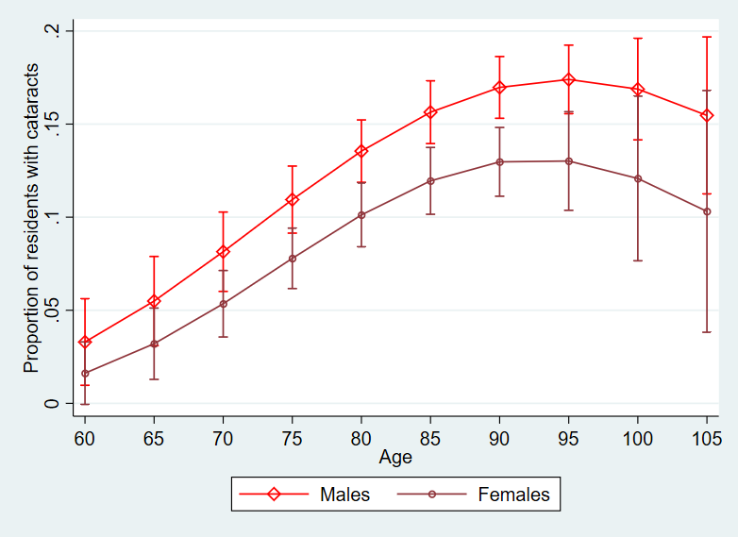


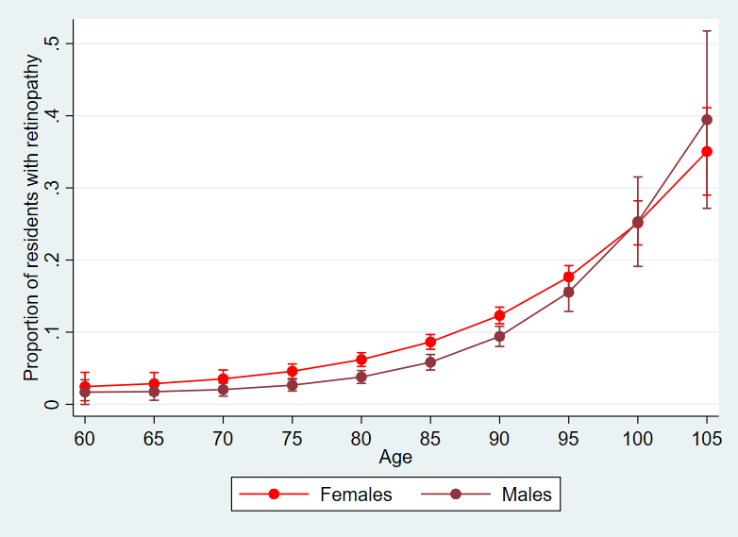

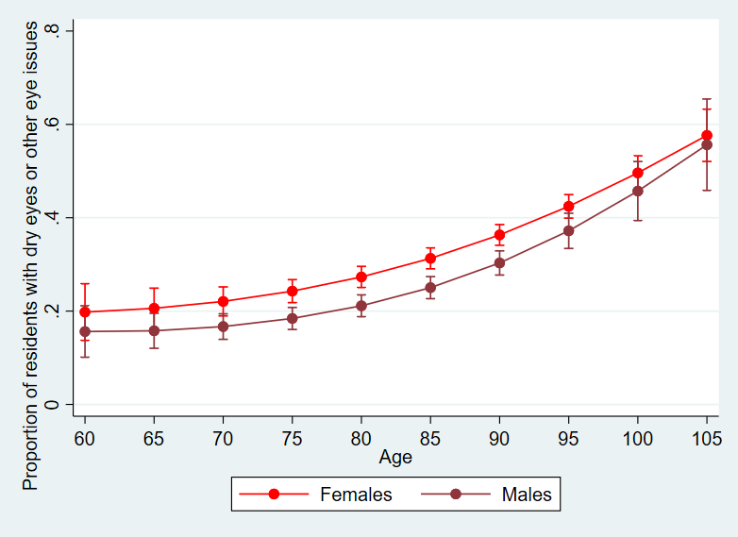


Supplemental Figure 2K. Proportion of Residents with Disorders of the Visual System by Age and Sex


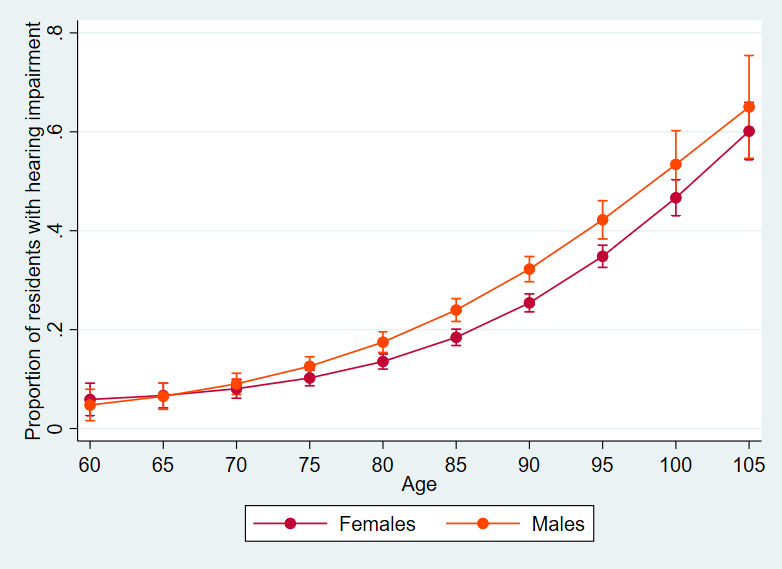


Supplemental Figure 2L. Proportion of Residents with Hearing Impairment by Age and Sex


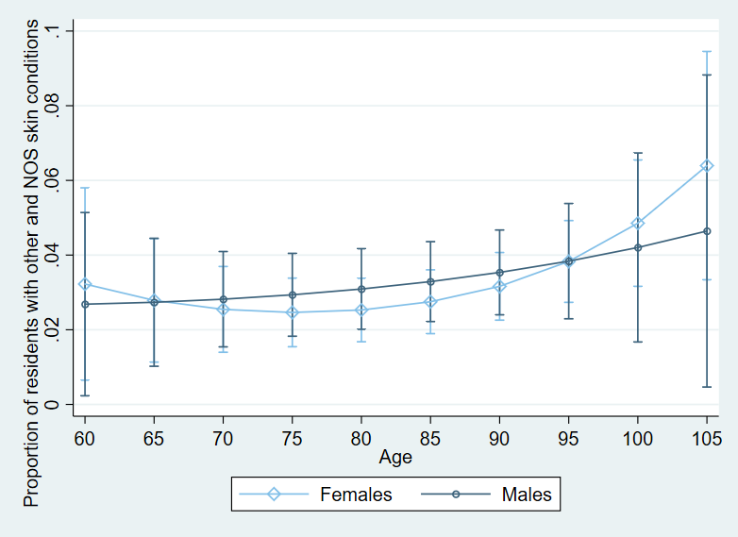

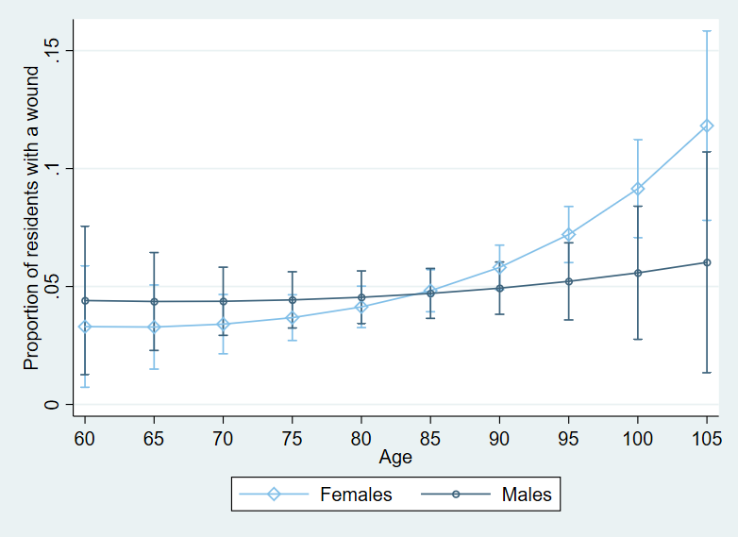


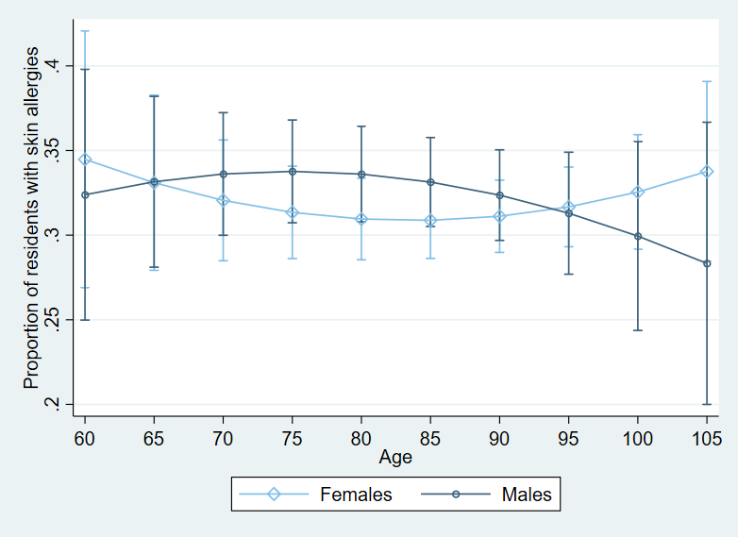

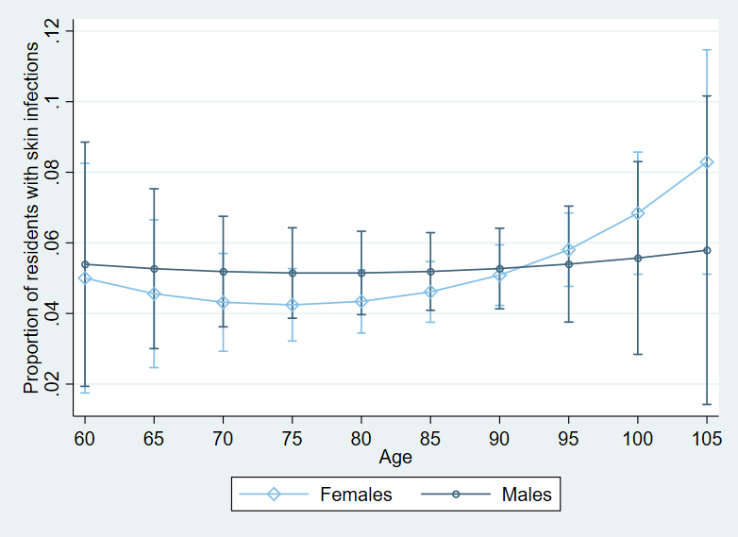


Supplemental Figure 2M. Proportion of Residents with Skin Conditions by Age and Sex
